# Pharmacological targeting of the protein synthesis mTOR/4E-BP1 pathway in cancer-associated fibroblasts abrogates pancreatic tumour chemoresistance

**DOI:** 10.15252/emmm.201404346

**Published:** 2015-04-01

**Authors:** Camille Duluc, Siham Moatassim-Billah, Mounira Chalabi-Dchar, Aurélie Perraud, Rémi Samain, Florence Breibach, Marion Gayral, Pierre Cordelier, Marie-Bernadette Delisle, Marie-Pierre Bousquet-Dubouch, Richard Tomasini, Herbert Schmid, Muriel Mathonnet, Stéphane Pyronnet, Yvan Martineau, Corinne Bousquet

**Affiliations:** 1 INSERM UMR-1037, Cancer Research Center of Toulouse (CRCT), Equipe labellisée Ligue Contre le Cancer and Laboratoire d'Excellence Toulouse Cancer (TOUCAN), Université de ToulouseToulouse, France; 2 Biochemistry-Immunology Laboratory, Faculty of Sciences Rabat, University Mohammed V – AgdalAgdal, Morocco; 3 EA 3842 Laboratory, Medicine and Pharmacy Faculties, Limoges UniversityLimoges, France; 4 Pathology Department, Hôpitaux de ToulouseToulouse, France; 5 CNRS UMR-5089, Institut de Pharmacologie et de Biologie structurale (IPBS), Université de ToulouseToulouse, France; 6 CRCM, INSERM, U1068; Paoli-Calmettes Institute; Aix-Marseille University, UM105; CNRS, UMR7258Marseille, France; 7 Novartis PharmaceuticalsBales, Switzerland

**Keywords:** cancer-associated fibroblasts, chemoresistance, pancreatic cancer, pharmacotherapy, protein synthesis and secretion

## Abstract

Pancreatic ductal adenocarcinoma (PDAC) is extremely stroma-rich. Cancer-associated fibroblasts (CAFs) secrete proteins that activate survival and promote chemoresistance of cancer cells. Our results demonstrate that CAF secretome-triggered chemoresistance is abolished upon inhibition of the protein synthesis mTOR/4E-BP1 regulatory pathway which we found highly activated in primary cultures of α-SMA-positive CAFs, isolated from human PDAC resections. CAFs selectively express the sst1 somatostatin receptor. The SOM230 analogue (Pasireotide) activates the sst1 receptor and inhibits the mTOR/4E-BP1 pathway and the resultant synthesis of secreted proteins including IL-6. Consequently, tumour growth and chemoresistance in nude mice xenografted with pancreatic cancer cells and CAFs, or with pieces of resected human PDACs, are reduced when chemotherapy (gemcitabine) is combined with SOM230 treatment. While gemcitabine alone has marginal effects, SOM230 is permissive to gemcitabine-induced cancer cell apoptosis and acts as an antifibrotic agent. We propose that selective inhibition of CAF protein synthesis with sst1-directed pharmacological compounds represents an anti-stromal-targeted therapy with promising chemosensitization potential.

## Introduction

Pancreatic ductal adenocarcinoma (PDAC) is one of the most intractable solid malignancies in humans. The survival rate at 5 years is < 5%. Due to a silent evolution for several years and the lack of biomarkers, patients usually have late-stage cancer, with metastasis at the time of diagnosis. Surgery, which is the only available strategy which may increase survival rate, is feasible in very few cases (< 15%), and patient survival rarely extends beyond 5 years. Standard chemotherapy using gemcitabine or targeted therapies directed at molecular alterations in cancer cells has provided almost no survival benefit in clinical trials, despite cytostatic results in *in vitro* and *in vivo* preclinical PDAC models (Hidalgo & Von Hoff, 2012). Therapeutic inadequacy may be attributed, in part, to the under-estimation of the influences exerted by the microenvironment on cancer cells, and the use of preclinical models that do not mimic this critical feature (Singh *et al*, 2010; Feig *et al*, 2012; Perez-Mancera *et al*, 2012).

PDAC is one of the most stroma-rich cancers, with the stroma forming more than 80% of the tumour mass. The most abundant cells present in PDAC stroma are α-SMA (alpha-smooth muscle actin)-expressing cancer-associated fibroblasts (CAFs), which in the pancreas are also called activated pancreatic stellate cells. PDAC stroma also contains immune, inflammatory and nerve cells and blood vessels, surrounded by acellular components which form the extracellular matrix (ECM) (Erkan *et al*, 2012b; Feig *et al*, 2012). These features have been observed in other advanced stage carcinomas (e.g. breast cancer) (De Palma & Hanahan, 2012).

In the pancreas, fibroblasts are involved in the deposition of ECM and the secretion of soluble factors (e.g. growth factors), which regulate normal epithelial differentiation and homeostasis (Apte *et al*, 2012). Upon ‘activation’ during inflammation, fibroblasts are the principal source of ECM constituents and are considered to be the main mediators of scar formation and tissue fibrosis and also secrete large amounts of growth and inflammatory factors. Once the wound is repaired, the resting phenotype is thought to be restored. Conversely, as with organ fibrosis, CAFs at the site of a tumour remain perpetually activated.

In PDACs, the abundant fibrotic stroma produced by CAFs constitutes a mechanical scaffold and a physical barrier against the effective delivery of therapeutic agents (Olive *et al*, 2009). Antifibrotic therapy therefore appears promising for the treatment of PDAC, although it is a ‘symptomatic’ and non-selective strategy (Erkan, 2013). Besides secreting fibrillar ECM components, CAFs secrete soluble growth, angiogenic and inflammatory factors, that engage in cancer and other stromal cell survival and metastatic and angiogenic signalling that promotes tumour growth and invasion (Hwang *et al*, 2008; Vonlaufen *et al*, 2008). Importantly, the signals stimulated by CAFs in cancer cells are redundant to those targeted by therapies, conferring innate resistance (Apte *et al*, 2013; Erkan, 2013). Since CAFs are master ‘secretors’ of soluble and insoluble factors which form these specific stromal features, we hypothesized that targeting CAF secretion would represent a specific therapeutic option for PDAC. Further understanding of the mechanisms governing CAF secretion may assist in the development of novel therapies to overcome CAF-triggered drug resistance. In this manuscript, the critical role of mTOR/4E-BP1 signalling pathway activation in promoting protein synthesis and secretion in CAFs has been elucidated. Additionally, a specific pharmacological strategy to stop protein synthesis and secretion through inhibition of this pathway in CAFs is proposed as a novel promising strategy, which has to be used in combination with chemotherapy in the treatment of PDAC.

## Results

**Figure 1:**
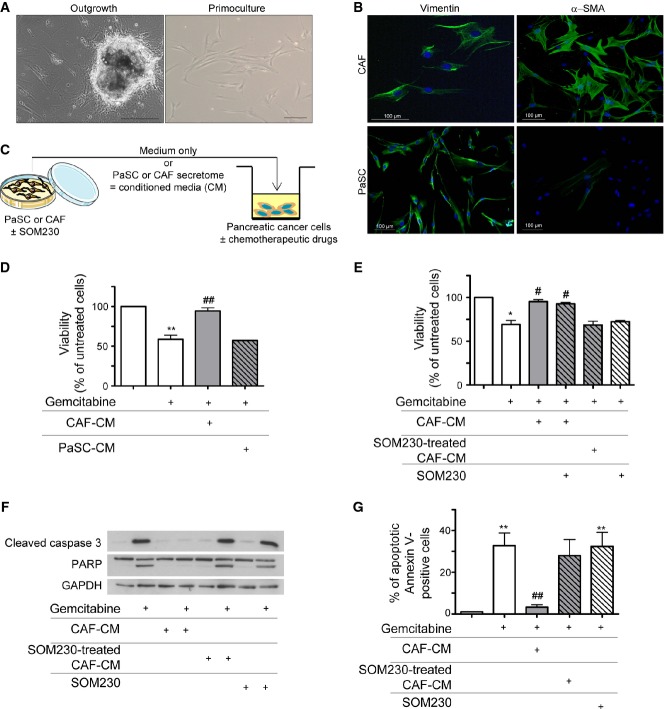
CAFs, but not PaSCs, secrete soluble proteins providing pancreatic cancer cell resistance to gemcitabine that is inhibited by CAF pre-treatment with SOM230

The normal human exocrine pancreas is essentially composed of acinar and ductal cells whose network is supported by a discrete extracellular matrix, present mostly at the interlobular spaces and around the tubular ductal structures (Supplementary Fig S1A, H&E and Masson's trichrome stainings). In contrast, PDAC is rich in stroma produced by α-SMA-expressing CAFs that reside both inside the tumour and at the boundaries between the invasive cancer and the host pancreatic tissue. In the normal human pancreas, these cells are present in an inactive α-SMA-negative state (Supplementary Fig S1A). It has been shown that CAFs confer chemoprotective features on pancreatic cancer cells (Hwang *et al*, 2008), though the underlying mechanisms and, consequently, targets for chemoprotection inhibition remain unclear. To explore this, primary CAF cultures were established by the outgrowth method (Fig1A) (Erkan *et al*, 2012a) from fifteen surgically obtained human resected PDAC tumours of different disease stages (Supplementary Table S1). After a few days, cells that migrated from the tumour tissues exhibited a fibroblast-like phenotype as confirmed by the expression of vimentin and were considered ‘activated’ since nearly 100% also expressed α-SMA (Fig1B). This phenotype was maintained throughout 10 passages before senescence occurred (not shown). By contrast, vimentin-positive pancreatic stellate cells (PaSCs), obtained from normal human pancreas samples, did not express α-SMA and were considered to be non-activated (Fig1B). The doubling-time period was longer in CAFs (6 days) than in PaSCs (2-days) (Supplementary Fig S1B), and most α-SMA-positive cells in PDAC were Ki67 negative, whereas a significant number of cancer cells were positive for Ki67 (Supplementary Fig S1C). Interestingly, CAFs secreted two-fold more proteins than PaSCs, as measured in their respective secretomes (hereafter referred to as conditioned media, CM) (Supplementary Fig S1D). We hypothesized that activation of PaSCs into CAFs correlated with an increased secretion of factors that mediate *de novo* pancreatic cancer cell resistance to chemotherapy (Meads *et al*, 2009).

A CAFs isolation from human pancreatic tumour resections (left panel) and *in vitro* primo-culture (right panel).

B Isolated CAFs and PaSC characterization *in vitro* by immunofluorescence using anti-vimentin (left panel) or anti-α-SMA (right panel) antibody (one representative field of *n* = 3).

C Experimental method schematic representation. CAFs or PaSC was treated or not with SOM230 (10^−7 ^M) for 48 h. Conditioned media (CM) were collected. Pancreatic cancer cells were incubated for 48 h with the indicated CM, in the presence or not of gemcitabine (100 μg/ml).

D, E Panc-1 cell viability was assessed by MTT. Results (mean ± SD) are presented for each treatment (CM from PaSC, or from CAF, or from CAF ± SOM230, SOM230) as a percentage of the respective gemcitabine-untreated cells (= 100%) (*n* = 4; from left to right: **P = 0.007, ^##^*P* = 0.006 in D; *P = 0.032, ^#^*P* = 0.041, ^#^*P* = 0.047 in E).

F Apoptosis induced by gemcitabine was evaluated by Western blot using an anti-cleaved caspase-3 or anti-PARP antibody (representative of *n* = 3).

G Panc-1 was analysed by flow cytometry. Percentages (mean ± SD) of annexin V-positive cells are indicated (*n* = 3; from left to right: **P = 0.005, ^##^*P* = 0.004, **P = 0.006).

Data information: * effect of treatment (gemcitabine or SOM230) versus untreated cells; ^#^CM-incubated versus non-incubated gemcitabine-treated cells. Source data are available online for this figure.

To verify this possibility, the chemosensitivity of different pancreatic cancer cell lines was tested in the presence of CM from CAFs or PaSCs that had been grown for 48 h without foetal calf serum (passages 3 to 8) (Fig1C). An MTT viability assay demonstrated that the cytotoxic action of gemcitabine (Fig1D), 5-fluorouracil (5FU) or oxaliplatin (Supplementary Fig S2A–B) on pancreatic Panc-1 cancer cells was completely reversed upon Panc-1 cell co-treatment with CM from CAFs, whereas CM from PaSCs did not provide any chemoprotection. In addition, heating CM to 95°C significantly diminished the chemoprotective capacity, suggesting a significant role of proteins in the process (Supplementary Fig S2C).

We hypothesized that the chemoprotective features of the CAF secretome are derived from soluble paracrine peptides or proteins that could be targeted using pharmacological drugs currently used as medical treatments to inhibit excessive hormone/peptide secretions from neuroendocrine tumours, that is somatostatin analogues. To test this hypothesis, Panc-1 cell response to gemcitabine, 5FU or oxaliplatin was assessed after 48 h of treatment with CM from CAFs, which had previously been incubated with or without a multi-receptor somatostatin analogue SOM230 (Pasireotide® Novartis) (Fig1C). SOM230 did not directly inhibit CAF proliferation or survival, nor did it reduce expression of the CAF marker α-SMA (not shown). Importantly, the chemoprotective property of CM from CAFs was dose-dependently reversed once CAFs had first been treated with SOM230 (Fig1E and Supplementary Fig S2D–F). When applied directly to Panc-1 cells, SOM230 alone had no direct cytotoxic effect in addition to the chemotherapeutic drugs, and nor did it inhibit the chemoprotection induced by CAF-CM (Fig1E). Various apoptosis assays (caspase-3 and PARP cleavage, Fig1F and S2G–H; apoptotic annexin V-positive cells, Fig1G; executioner caspase activity, Supplementary Fig S3A) confirmed the cytotoxic action of chemotherapies on pancreatic cancer Panc-1 cells, which was abolished upon Panc-1 co-treatment with CM from CAFs, but restored when CM from CAFs treated with SOM230 was used. These results were also confirmed in two additional pancreatic cancer cell lines, Capan-1 and BxPC-3 cells (Supplementary Fig S3B–D). The calculated IC50 values for gemcitabine cytotoxicity on Panc-1 and BxPC-3 cells incubated with or without the CM from CAFs, previously treated with or without SOM230, are shown in Supplementary Fig S3E–G. These data demonstrate a large potential therapeutic benefit of treating CAFs with SOM230 to enhance pancreatic cancer cell sensitivity to different chemotherapeutic drugs.

### High protein synthesis in CAFs through mTORC1 activation is responsible for the secretion of chemoprotective factors—phenotypic reversion with the somatostatin analogue SOM230

**Figure 2:**
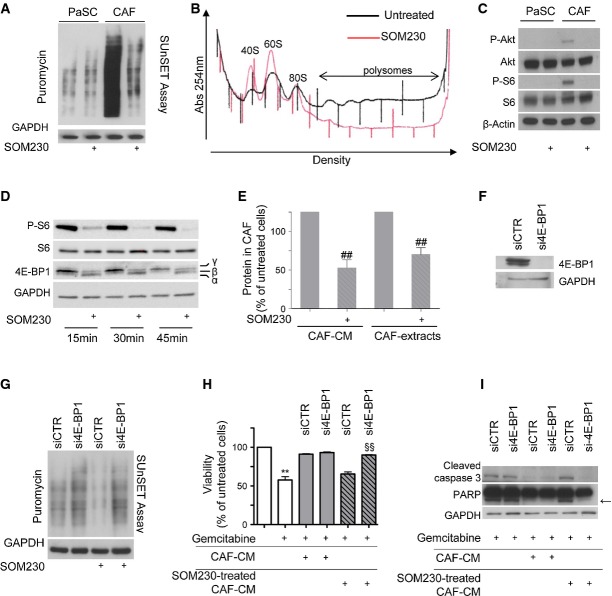
High protein synthesis in CAFs mediates chemoprotective effect on pancreatic cancer cells—reversion through inhibition of protein synthesis with SOM230

We hypothesized that the chemoprotective property of CAFs relies on elevated synthesis and secretion of soluble growth factors, cytokines and/or chemokines. To test this hypothesis, protein synthesis rates were first monitored by SUnSET (a non-radioactive equivalent of the ^35^S-Met assay based on puromycin incorporation into nascent polypeptides) (Schmidt *et al*, 2009) in human CAFs and PaSCs which were previously serum-starved for a period or 48 h. CAFs incorporated much more puromycin into nascent polypeptides than PaSCs (Fig2A, compare lanes 1 and 3). This high rate of protein synthesis was totally suppressed upon SOM230 treatment, while no effect was detected for PaSCs (Fig2A). The inhibitory effect of SOM230 on CAF protein synthesis was further confirmed by a polysomal fractionation assay, which demonstrated that much fewer polysomes (containing translated mRNAs) were formed upon treatment with the somatostatin analogue (Fig2B). The high rate of protein synthesis might be due to activation of the mTORC1 pathway, a strong regulator of mRNA translation. This is further supported by our previous data showing that somatostatin analogues inhibit PI3K activity (Bousquet *et al*, 2006). Consistently, the PI3K/mTOR targets Akt and S6 appeared to be intrinsically phosphorylated (i.e. activated) and fully sensitive to SOM230 treatment in CAFs while not constitutively activated and hence not inhibited by SOM230 in PaSCs (Fig2C). Similarly, 4E-BP1, which is considered to be the major mTORC1 target regulating mRNA translation (Martineau *et al*, 2013), was severely hypophosphorylated (i.e. active in inhibiting translation) upon CAF treatment with SOM230 (Fig2D). Consistently, global protein concentrations in CAF extracts and CM were dramatically decreased by SOM230 treatment (Fig2E) although SOM230 did not affect total RNA concentration (Supplementary Fig S4A), indicating that SOM230-triggered inhibition of protein synthesis may be correlated to an inhibition of the mTORC1/4E-BP1 axis. Silencing of 4E-BP1 using specific siRNA (si4E-BP1) (Fig2F) circumvented the inhibitory effects of SOM230 on CAF protein synthesis (Fig2G) and on CAF-CM-dependent protection against gemcitabine-triggered cancer cell viability (Fig2H) and apoptosis (Fig2I). Furthermore, SOM230 was shown to be at least as potent as the mTORC1 (RAD001) and mTOR (PP242) inhibitors at suppressing protein synthesis and re-sensitizing pancreatic cancer cells to gemcitabine cytotoxicity (Supplementary Fig S4B–C).

Immunoblotting of protein extracts from PaSCs or CAFs treated (+) or not with SOM230 (10^−7^ M) for 48 h, using the anti-puromycin antibody (representative of *n* = 3).

Polysomes profiles of CAFs treated or not with SOM230 for 48 h (representative of *n* = 3).

Immunoblotting using an anti-P-Akt, anti-P-S6 or anti-β-actin (loading control) antibody of protein extracts from PaSCs or CAFs treated (+) with SOM230 for 30 min (representative of *n* = 3).

Immunoblotting of protein extracts from CAFs treated (+) or not with SOM230 (10^−7 ^M) for the indicated times, using anti-P-Akt, anti-P-S6 or anti-4E-BP1 antibody (representative of *n* = 3).

Protein concentration in untreated or SOM230-treated CAF-CM or extracts normalized per 1 × 10^6^ cells. Results (mean ± SD) are presented as a percentage of the untreated CAFs (= 100%) (*n* = 3; from left to right: ^##^*P* = 0.008, ^##^*P* = 0.007).

Immunoblotting using an anti-4E-BP1 or anti-GAPDH (loading control) antibody of protein extracts from siCTR- or si4E-BP1-transfected CAFs (representative of *n* = 3).

Immunoblotting of equal amounts of protein from siCTR- or si4E-BP1-transfected CAFs treated (+) or not with SOM230 for 48 h, using the anti-puromycin antibody (representative of *n* = 3).

Panc-1 cell viability was assessed by MTT. Panc-1 cells were incubated with gemcitabine in the presence of CM from untreated or SOM230-treated CAFs transfected with the siCTR or si4E-BP-1. Results (mean ± SD) are presented as a percentage of the untreated CAFs (= 100%) (*n* = 3; **P = 0.003, ^§§^*P* = 0.002).

Caspase-3 and PARP cleavage induced by gemcitabine in Panc-1 was evaluated by Western blot using the respective antibodies (representative of *n* = 3). Arrow indicates cleaved forms of PARP.

Data information: * gemcitabine-treated versus gemcitabine-untreated cells; ^#^ SOM230-treated versus SOM230-untreated cells; ^§^ si4E-BP1-transfected versus siCTR-transfected cells. Source data are available online for this figure.

These results demonstrate that through constitutive neutralization of the translational repressor 4E-BP1 by intrinsic activation of the mTORC1 pathway, the rate of protein synthesis is permanently higher in CAFs than in PaSCs. The data also reveal that abrogation of the chemoprotective property of CAF-CM by SOM230 results from its ability to efficiently inhibit 4E-BP1 phosphorylation in CAFs.

### The somatostatin receptor sst1 mediates SOM230 effects on CAFs

**Figure 3:**
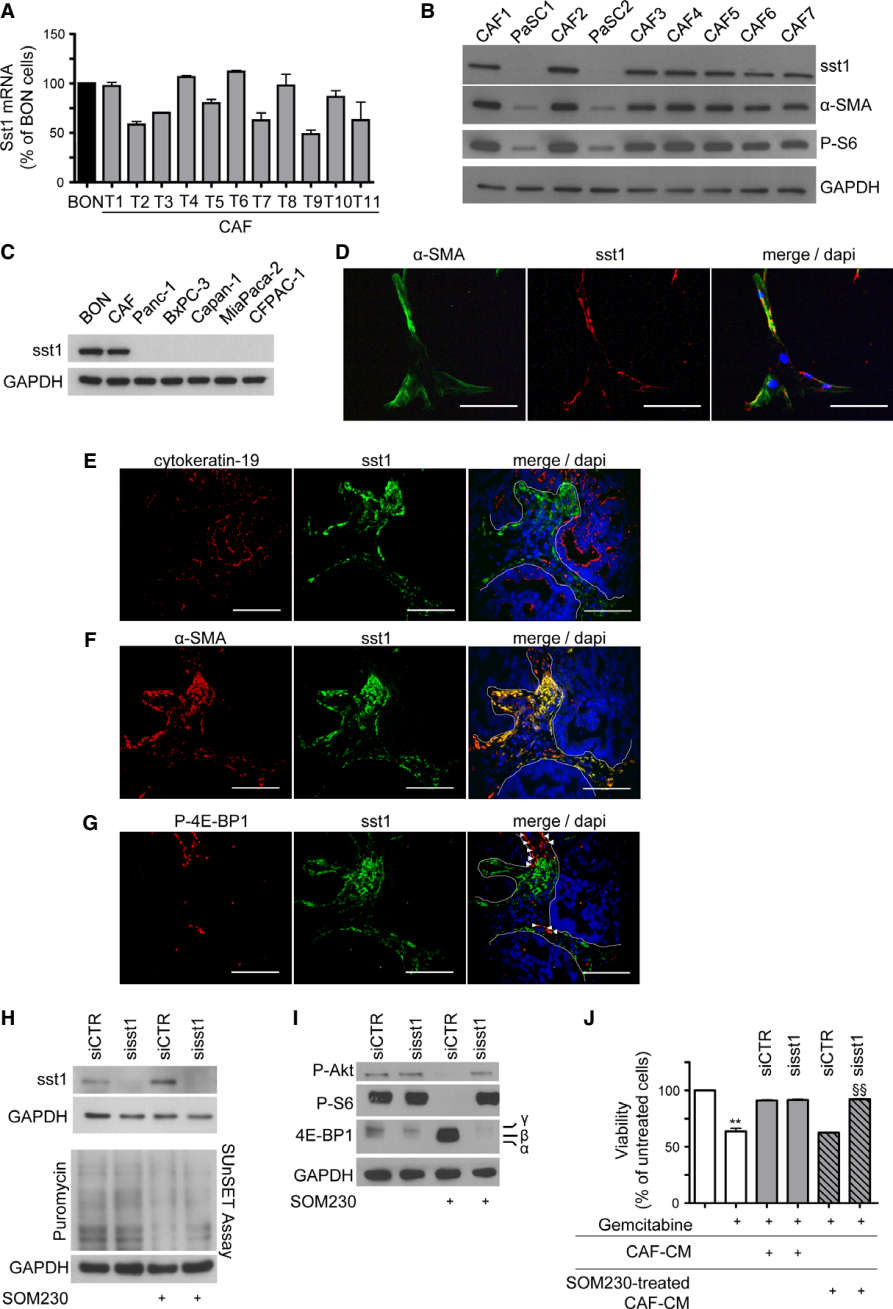
CAFs express the somatostatin receptor subtype 1 sst1 that mediates SOM230 inhibitory effect on CAF-induced chemoprotection

Somatostatin mediates its effects through five different G protein-coupled receptors named sst1–sst5. However, as the low affinity of SOM230 for sst4 (Schmid, 2008) is not compatible with the effects observed in CAFs, we suspected the involvement of one of the other four receptors. Consistently, qRT–PCR experiments performed on CAFs isolated from fifteen different patients revealed that sst1 was the only somatostatin receptor expressed in CAFs (not shown). Furthermore, it was shown that CAFs expressed sst1 as efficiently as neuroendocrine pancreatic tumour BON cells, which are known to contain high levels of sst1 (Xiao *et al*, 2012) (Fig3A). Western blot analyses showed that neither PaSCs (Fig3B) nor other pancreatic cancer cell lines (Fig3C) expressed sst1. Immunofluorescence analyses localized sst1 to α-SMA-positive CAF primary cultures (Fig3D). Knock-down of sst1 by RNA interference (siRNA targeting sst1, sisst1) in CAFs demonstrated the specificity of the signal detected by the anti-sst1 antibody (Western blot and immunofluorescence, Supplementary Fig S5A–B). Immunofluorescence confocal microscopy analyses of serial sections of 42 human PDACs demonstrated that sst1 and cytokeratin-19 staining did not co-localize, demonstrating that sst1 was not expressed in epithelial cancer cells (Fig3E). In contrast, sst1 staining was present in 69% ± 19 of α-SMA-positive stromal cells (quantified in *n* = 42 PDACs) (Fig3F, Supplementary Table S2A). In PDAC stroma, all sst1-positive cells expressed α-SMA (Fig3F). A significant proportion of sst1-positive (40% ± 16) and α-SMA-positive (39% ± 17) cells (quantified in *n* = 15 PDACs) also yielded positive and correlated staining for the phosphorylated (inhibited) form of 4E-BP1 (*r* = 0.96), indicating PI3K/mTOR pathway activation in sst1-expressing α-SMA-positive cells (Fig3G, Supplementary Fig S5C, Supplementary Tables S2B–C). Knock-down of sst1 in CAFs (sisst1) demonstrated the importance of this G protein-coupled receptor (GPCR) in SOM230-triggered inhibition of the PI3K/mTOR pathway (dephosphorylation of Akt, S6 and 4E-BP1) and protein synthesis (Fig3H–I), and restoration of chemosensitivity (Fig3J). The data also indicated that treatment of CAFs with SOM230 did not alter sst1 expression (Fig3H, top). These results demonstrate that SOM230 affects protein translation and the secretion of soluble chemoprotective factors in CAFs via the sst1 receptor. Accordingly, CAF treatment with another somatostatin analogue octreotide, which does not activate sst1 (Schmid, 2008), was not able to reverse the chemoprotective effect conferred by the corresponding CAF-CM, nor directly regulate apoptosis, on gemcitabine-treated pancreatic cancer cells (Panc-1 or BxPC-3 cells), as measured by various apoptosis and survival assays (executioner caspase activity, Supplementary Fig S6A–B; MTT, Supplementary Fig S6C–D; caspase-3 and PARP cleavage, Supplementary Fig S6E).

A Expression of the somatostatin receptor subtype 1 (sst1) analysed by RT–qPCR in the human pancreatic endocrine cell line BON and in CAFs isolated from 11 different patients (mean ± SD).

B Immunoblotting of protein extracts from PaSCs and CAFs, using anti-sst1, anti-α-SMA, anti-P-S6 or anti-GAPDH (loading control) antibody (representative of *n* = 3).

C Immunoblotting of protein extracts from BON, CAFs and indicated pancreatic cancer cell lines, using anti-sst1 or anti-GAPDH (loading control) antibody (representative of *n* = 3).

D Immunofluorescence confocal microscopy analyses of α-SMA and sst1 in CAFs. Co-localization of α-SMA and sst1 is shown (merge) (one representative field of *n* = 3) (scale bar = 100 μm).

E–G Representative immunofluorescence confocal microscopy analyses of sst1 co-localization with cytokeratin-19 (*n* = 42 PDAC samples) (E), α-SMA (*n* = 42 PDAC samples) (F) or with P-4E-BP1 (*n* = 25 PDAC samples) (G) in serial slides (scale bar = 100 μm). Dashed white lines represent the limits between stroma and tumour epithelial glands.

H, I Immunoblotting of protein extracts from siCTR- or sisst1-transfected CAFs treated (+) or not with SOM230 for 48 h (sst1, puromycin and GAPDH as loading control, H) or for 30 min (P-Akt, P-S6, 4E-BP1 and GAPDH as loading control, I) (representative of *n* = 3).

J Panc-1 cell viability was assessed by MTT. Panc-1 cells were incubated with gemcitabine in the presence of CM from untreated or SOM230-treated CAFs transfected with siCTR or si-sst1. Results (mean ± SD) are presented as the percentage of the untreated cells (= 100%) (*n* = 3; **P = 0.003, ^§§^*P* = 0.002).

Data information: * gemcitabine-treated versus gemcitabine-untreated cells; ^§^ si-sst1-transfected versus siCTR-transfected cells. Source data are available online for this figure.

### Mechanisms underlying sst1 inhibitory effect on the PI3K/mTOR pathway in CAFs

**Figure 4:**
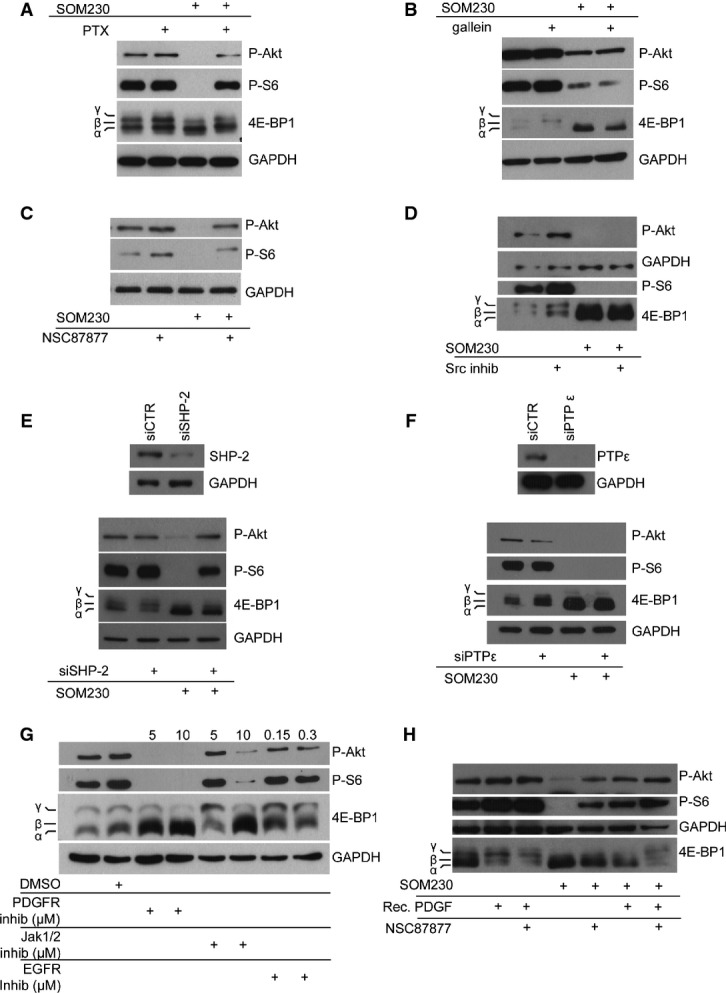
Molecular mechanisms of SOM230 action through sst1 for inhibition of Akt-4E-BP1 signalling pathways in CAF

Because sst1 is a GPCR, we treated CAFs with an inhibitor of either G_α_i (PTX, pertussis toxin) or G_βγ_ (gallein) (Fig4A–B). PTX, but not gallein, reversed the SOM230 inhibitory effect on PI3K/mTOR pathway activation, demonstrating that G_α_i and not G_βγ_ mediates this signal downstream of sst1. It has been previously demonstrated that sst1 induces the activation of phosphotyrosine phosphatases, including the SH2-containing SHP-2 and PTPε. This successive activation also involves the activity of Src (Arena *et al*, 2007). To test the involvement of this protein complex in the SOM230-induced inhibition of the PI3K/mTOR pathway, CAFs were treated with the phosphotyrosine phosphatase inhibitor NSC87877 (which inhibits SHP-1, SHP-2 and PTPε) (Fig4C). The effects of SOM230 were potently reversed. However, a Src inhibitor had no effect on the mTOR signalling pathway (Fig4D). In CAFs, SHP-2 knock-down by specific siRNA (siSHP-2) also reversed the SOM230 inhibitory effect on the PI3K/mTOR pathway (Fig4E), in contrast to PTPε knock-down (Fig4F). We then investigated which phosphoprotein in the PI3K/mTOR pathway could be the target of SOM230-induced phosphotyrosine phosphatase activity. We searched for upstream signals that could induce basal activation of the PI3K/mTOR pathway in CAFs. Because platelet-derived growth factor (PDGF) is a well-known signal that activates PaSCs (Bachem *et al*, 2005) and also induces PaSC migration through PI3K activation (McCarroll *et al*, 2004), these cells were treated with a PDGF receptor inhibitor which efficiently inhibited basal activation of the PI3K/mTOR pathway, in contrast to the EGFR inhibitor (Fig4G). CAF treatment with a JAK1/2 inhibitor also suppressed the activation of this pathway. These results indicate that, in CAFs, the PI3K/mTOR pathway is activated by the autocrine secretion of PDGF and JAK2-dependent cytokines. Interestingly, addition of recombinant PDGF to CAF further enhanced activation of this pathway (Fig4H). SOM230 inhibited PDGF-induced activation of the PI3K/mTOR pathway by dephosphorylation of Akt, S6 and 4E-BP1, which was reversed by the phosphotyrosine phosphatase inhibitor NSC87877. These results demonstrate that SOM230 inhibits basal and PDGF-induced activation of the PI3K/mTOR pathway in CAFs through activation of phosphotyrosine phosphatases including SHP-2.

A–D Immunoblotting using an anti-P-Akt, anti-P-S6, anti-4EB-P1 or anti-GAPDH (loading control) antibody of protein extracts from CAFs pre-treated (+) or not overnight with PTX (100 ng/ml) (A), gallein (10 μM) (B), NSC87877 (10 μM) (C), Src inhibitor (5 μM) (D), treated (+) or not with SOM230 (10^−7 ^M) for 30 min (representative of *n* = 3).

E, F Immunoblotting using anti-P-Akt, anti-P-S6, anti-4EB-P1 or anti-GAPDH (loading control) antibody of protein extracts from CAF siCTR- or siSHP2- (E) or siPTPε- (F) transfected CAF treated or not with SOM230 for 30 min (representative of *n* = 3).

G, H Immunoblotting using an anti-P-Akt, anti-P-S6, anti-4EB-P1 or anti-GAPDH (loading control) antibody of protein extracts from CAFs treated with the indicated molecules (PDGF receptor inhibitor, 5–10 μM; Jak1/2 inhibitor ruxolitinib, 5 or 10 μM; EGFR inhibitor, 150 or 300 nM and recombinant PDGF, 5 μg/ml) (representative of *n* = 3).

Source data are available online for this figure.

### SOM230 sensitizes pancreatic cancer cells to gemcitabine cytotoxicity *in vivo*

**Figure 5:**
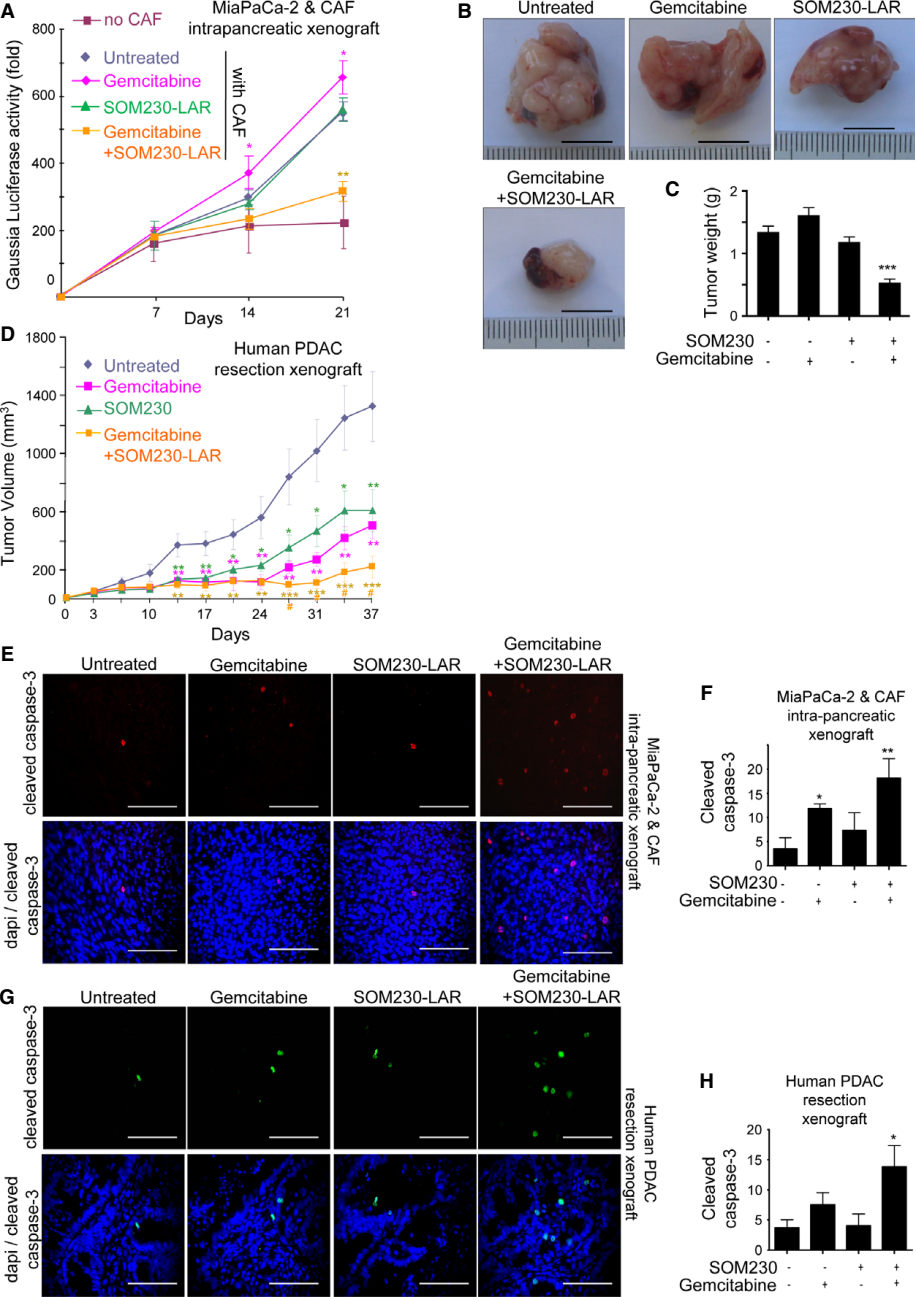
SOM230 increases *in vivo* sensitivity to gemcitabine of tumour xenograft (MIA PaCa-2-Luc cells and CAFs or human PDAC resection)

To verify that SOM230 is capable of inhibiting the chemoprotective features of CAFs, SOM230 efficacy was tested in combination with gemcitabine *in vivo*, in athymic mice that had been xenografted either with human pancreatic cancer cells (MIA PaCa-2 or Panc-1 cells, orthotopically or sub-cutaneously, respectively) with or without CAFs, or with pieces of human pancreatic tumour resections (sub-cutaneous PDX, patient-derived tumour xenograft) (Fig5, Supplementary Figs S7–S8). To dynamically estimate the growth of intra-pancreatic tumours, xenografted MIA PaCa-2 cells were first transduced with a lentivector expressing secreted Gaussia Luciferase, whose activity, when measured in mouse plasma, reflects tumour growth non-invasively. Mice were treated with or without SOM230-LAR, a long-acting release form of SOM230, and with gemcitabine. Our results show that cancer cells (MIA PaCa-2 or Panc-1) alone were unable to form tumours (Fig5A and Supplementary Fig S8A), even up to 9 weeks after sub-cutaneous cell grafting (Supplementary Fig S8A). In contrast, tumours resulting from the cancer cell and CAF co-xenografts showed exponential growth (Fig5A and Supplementary Fig S8A). Interestingly, SOM230-LAR or gemcitabine treatment did not affect the growth of these combined cancer cell and CAF tumours (Fig5A and Supplementary Fig S8B), which was confirmed at sacrifice (tumour weight) (Fig5B–C and Supplementary Fig S8C). Importantly, the gemcitabine + SOM230-LAR bi-therapy decreased tumour growth of cancer cell and CAF co-xenograft tumours (Fig5A and Supplementary Fig S8B). Strikingly, the synergistic inhibition of pancreatic tumour growth induced by the association between SOM230-LAR and gemcitabine has been further demonstrated in a mouse model which mimics human pancreatic tumour biology and involves the subcutaneous xenografting of pieces of human PDAC resections (PDX) (Fig5D). Consistently, the gemcitabine + SOM230-LAR bi-therapy-induced tumour growth reduction was associated with a dramatic increase in cell apoptosis (cleaved caspase-3 and TUNEL), and an inhibition of cell proliferation (Ki67) (Fig5E–H, Supplementary Figs S7A–B, D and S8D–E) in the three tumour mouse models was tested. A decrease in collagen deposition (Masson's trichrome stain) and α-SMA staining was also consistently observed with the gemcitabine + SOM-LAR bi-therapy in these models (Supplementary Figs S7A–C, E and S8D, F–G). Individual gemcitabine or SOM230-LAR therapy decreased the growth of human PDAC resections xenografted in mice (Fig5D), which was consistent with the faint pro-apoptotic effect of gemcitabine (Fig5H) and with the antifibrotic action of SOM230-LAR (Supplementary Fig S7B and E). Consistently, *in vitro* collagen type I synthesis by CAFs was increased when compared to PaSCs, and was decreased upon treatment with SOM230, as evidenced by reduced production and deposition of soluble and insoluble collagens in both CAF-CM and cell extracts (Supplementary Fig S9A–E). These results demonstrate that *in vivo* gemcitabine treatment of pancreatic tumours containing abundant ECM bundles is inefficient (cancer cell and CAF co-xenografted models) or only partially effective (human PDAC resection xenografted model) at reducing tumour growth. In contrast, gemcitabine + SOM230-LAR bi-therapy yielded potent therapeutic benefits in all tested models, demonstrating that SOM230 co-treatment facilitates gemcitabine cytotoxicity *in vivo*. The expression of sst1 in α-SMA-positive fibroblasts, present in tumours (Supplementary Fig S7F–G), is compatible with a direct inhibitory effect of SOM230 on CAFs *in vivo* (matrix deposition).

A–C MIA PaCa-2-GLuc cells were injected with or without CAFs into the pancreas of nude mice. Mice were treated with each indicated treatment (SOM230-LAR at day 7 and gemcitabine at days 7, 10, 14 and 17), and the plasmatic luciferase activity was measured (mean ± SD) at days 7, 14 and 21 (*n* = 5) (*P* = 0.003 for gemcitabine + SOM230LAR versus untreated) (A). Tumours were excised, photographed (scale bars are 1 cm) (B), weighted (*P* = 0.0008) (C) and paraffin-embedded for immunohistofluorescence analyses using an anti-cleaved caspase-3 antibody (E).

D Human tumours were subcutaneously xenografted in nude mice and tumour volumes (mean ± SD) measured (at day 37, *P* = 0.0009 for gemcitabine + SOM230LAR versus untreated and *P* = 0.021 for gemcitabine + SOM230LAR versus gemcitabine). Mice were treated with the indicated treatments (SOM230-LAR at days 3 and 31, and gemcitabine at day 3 and twice a week thereafter).

E, F Tumours excised from mice as in (A-C) were paraffin-embedded for immunohistofluorescence analyses using an anti-cleaved caspase-3 antibody (scale bar = 100 μm) (E). Cleaved caspase-3 quantification was performed by counting the mean number of positive cells per field in five independent tumours (mean ± SD) (F) (*P = 0.036, **P = 0.009).

G, H Tumours excised from mice as in (D) were paraffin-embedded for immunohistofluorescence analyses using an anti-cleaved caspase-3 antibody (scale bar = 100 μm) (G). Cleaved caspase-3 quantification was performed by counting the mean number of positive cells per field in five independent tumours (mean ± SD) (H) (*P = 0.027).

Data information: * treated versus untreated xenograft. ^#^ SOM230-LAR + gemcitabine-treated versus gemcitabine-treated xenograft. *P < 0.05; **P < 0.01; ***P < 0.001.

### Mechanisms for CAF-mediated chemoprotection on pancreatic cancer cells—inhibition upon CAF co-treatment with SOM230

We reasoned that through secreted factors, CAFs may affect pancreatic cancer cell sensitivity to chemotherapeutic drugs and that SOM230 may inhibit this feature. IAPs (inhibitors of apoptosis) are a family of major anti-apoptotic factors that reduce cancer cell sensitivity to chemotherapies. Whereas XIAP is highly expressed in pancreatic cancer cells, survivin and other IAPs (cIAP1, cIAP2, livin) (not detected) are not (Supplementary Fig S10A). However, treatment with CAF-CM dramatically increased survivin, but not XIAP (or other IAPs, not detected), expression (Supplementary Fig S10A), suggesting a role for survivin (but not XIAP) in mediating CAF chemoprotection. Survivin expression was not further affected by gemcitabine treatment in the presence or absence of CAF-CM (Supplementary Fig S10B). Expression of survivin was not increased upon pancreatic cancer cell treatment with SOM230-treated CAF-CM, with or without gemcitabine. In CAFs, the ability of SOM230 to abrogate the stimulation of survivin expression induced by CAF-CM was abolished upon 4E-BP1 knock-down, indicating that this mechanism is dependent on the SOM230 inhibition of protein synthesis in CAFs (Supplementary Fig S10C). Decreasing survivin expression using an antisense oligonucleotide (Supplementary Fig S10D) partially reversed CAF-CM-induced chemoprotection in gemcitabine-treated pancreatic cancer cells (Supplementary Fig S10E–F), demonstrating that CAF-CM-induced expression of survivin represents one effector of CAF-promoted chemoresistance. Together, these results demonstrate that CAF-CM provides a *de novo* resistance of pancreatic cancer cells to chemotherapy, at least partially through diminished cancer cell sensitivity to the drug, which can be reversed upon CAF treatment with SOM230.

### IL-6 is a SOM230-druggable soluble factor, critical for the chemoprotective features of CAF secretions

**Figure 6:**
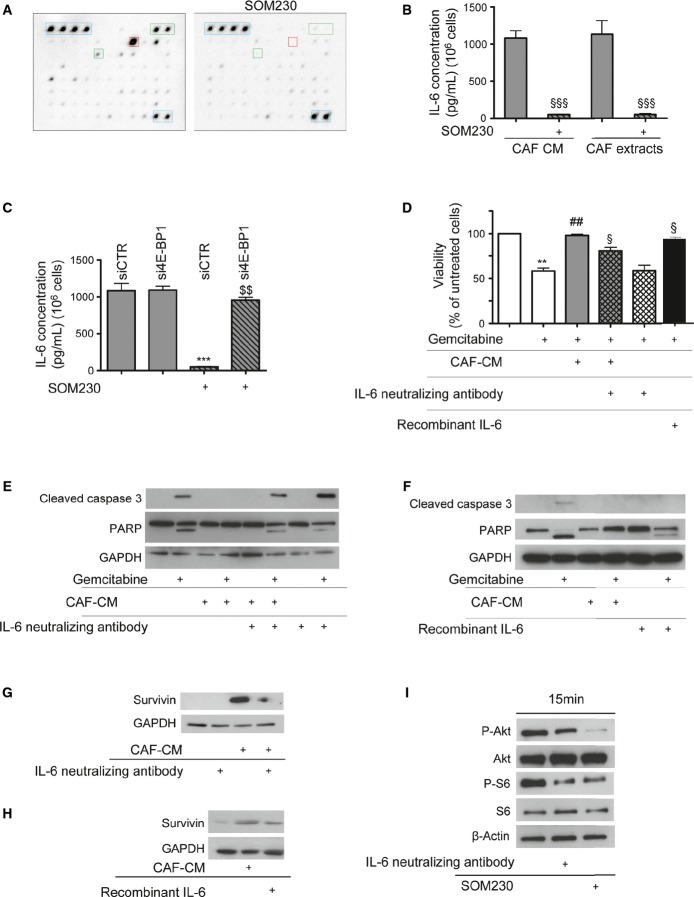
Identification of proteins differentially secreted by CAFs upon SOM230 treatment and involved in CAF-CM-induced chemoresistance on pancreatic cancer cells

Because protein synthesis is critical for CAF chemoprotection, we aimed to identify the chemoprotective factor(s) that are synthesized and secreted by CAFs and downregulated upon SOM230 treatment. We blotted a cytokine/chemokine antibody array membrane with the secretions from CAFs which had been previously treated with or without SOM230. Globally, among the 80 factors whose antibodies were present on the array, 60 were detectable in the CAF secretome (Supplementary Table S3). The expressions of 26 of these were significantly downregulated in CAF-CM upon CAF treatment with SOM230 (Supplementary Table S3, highlighted in grey). The PaSC secretome was found to be less rich than that of CAFs (Supplementary Fig S11A), which was consistent with results in Supplementary Fig S1D. Interleukin-6 (IL-6) was found to be the most abundant factor in the CAF secretome (Fig6A, red square), as confirmed by ELISA on either CAF extracts (intracellular proteins) or CAF-CM (secreted proteins) which quantified about 1 ng/ml of IL-6 produced per 10^6^ CAFs (Fig6B). Comparatively, PaSCs and pancreatic cancer Panc-1 and BxPC-3 cells expressed and secreted marginal amounts of IL-6 (< 0.1 ng/ml for 10^6^ cells) (Supplementary Fig S11B). Importantly, treatment with SOM230 abrogated IL-6 production by CAFs (Fig6B–C), whereas no effect was observed on IL-6 mRNA levels (Supplementary Fig S11C), suggesting an effect at the translational level. Consistently, knock-down of the translation inhibitor 4E-BP1 rendered CAFs resistant to the inhibitory effect of SOM230 upon intracellular expression and secretion of IL-6 (Fig6C). Similarly, two other soluble factors secreted by CAFs, but less abundantly by PaSCs, namely MCP-1 and Groα, presented a decreased protein expression in CM upon CAF treatment with SOM230 whereas expression of their mRNA was not affected (Supplementary Fig S11D–G).

A Membrane antibody array assay using CM from SOM230-treated or not CAFs (representative of *n* = 3). Controls are circled in blue dashed line and IL-6 in red square.

B, C Anti-IL-6 ELISA assay (mean ± SD) using CM or protein extracts from SOM230-treated or not CAF (from left to right: ^§§§^*P* = 0.0002, ^§§§^*P* = 0.0002) (B), or CM from siCTR- or si4E-BP1-transfected CAFs treated or not with SOM230 (from left to right: ***P = 0.0003, ^$$^*P* = 0.004) (C) (*n* = 3).

D Panc-1 cell viability was assessed by MTT. Results (mean ± SD) are presented for each treatment as the percentage of the respective untreated cells (= 100%) (*n* = 3; from left to right: **P = 0.004, ^##^*P* = 0.001, ^§^*P* = 0.023, ^§^*P* = 0.019).

E, F Immunoblotting using an anti-cleaved caspase-3, anti-PARP or anti-GAPDH (loading control) antibody of protein extracts from Panc-1 cells incubated with CAF-CM pre-incubated or not with an anti-IL-6 neutralizing antibody (E), or with human recombinant IL-6 (representative of *n* = 3) (F).

G, H Immunoblotting using an anti-survivin or anti-GAPDH (loading control) antibody of protein extracts from Panc-1 cells stimulated or not with CAF-CM pre-incubated or not with anti-IL-6 neutralizing antibody (G), or with human recombinant IL-6 (representative of *n* = 3) (H).

I Immunoblotting using an anti-P-Akt, anti-P-S6 or anti-β-actin (loading control) antibody of protein extracts from SOM230-treated or SOM230-untreated CAF incubated or not with IL-6 neutralizing antibody (representative of *n* = 3).

Data information: * gemcitabine-treated versus gemcitabine-untreated cells; ^#^ CM-incubated versus CM-non-incubated cells; ^§^ effect of treatment (SOM230 or anti-IL-6 neutralizing antibody, or recombinant IL-6) versus untreated cells; ^$^ si4E-BP1-transfected versus siCTR-transfected cells. Source data are available online for this figure.

The importance and functionality of CAF-secreted IL-6 on pancreatic cancer cell response to gemcitabine was evaluated. The IL-6 receptor was equally expressed in CAFs, PaSCs and pancreatic cancer cells (Supplementary Fig S11H). Blocking IL-6 activity in CAF-CM using an IL-6 neutralizing antibody significantly reversed the CAF-protective effects on cytotoxicity and apoptosis triggered by either gemcitabine (Fig6D–E), 5FU (Supplementary Fig S11I) or oxaliplatin (Supplementary Fig S11J). Conversely, treatment of cancer cells with 1 ng of recombinant IL-6 mimicked CAF chemoprotection, although less efficiently than whole CAF-CM, since a residual fraction of PARP remained cleaved (Fig6F). Consistently, neutralizing IL-6 activity decreased CAF-CM-induced survivin expression in pancreatic cancer cells, whereas treatment of cancer cells with recombinant IL-6 increased it, mimicking CAF-CM effects (Fig6G–H). Finally, we observed that activation of the mTORC1 pathway in CAFs, indicated by Akt and S6K phosphorylation, appeared to be partially dependent on the autocrine action of secreted IL-6 (Fig6I) and was consistent with the inhibition of this pathway by a JAK2 inhibitor (Fig4G), therefore highlighting a positive feedback of IL-6 on its own synthesis. Taken together, these results demonstrate the important role of CAF-secreted IL-6 in pancreatic cancer cell resistance to chemotherapies. They also identify a novel translation-dependent feed-forward loop sustaining IL-6 synthesis and secretion in CAFs, which is efficiently druggable by SOM230.

### IL-6 is a cytokine abundantly secreted by pancreatic lesions during tumorigenesis, in correlation with the abundance of CAFs

**Figure 7:**
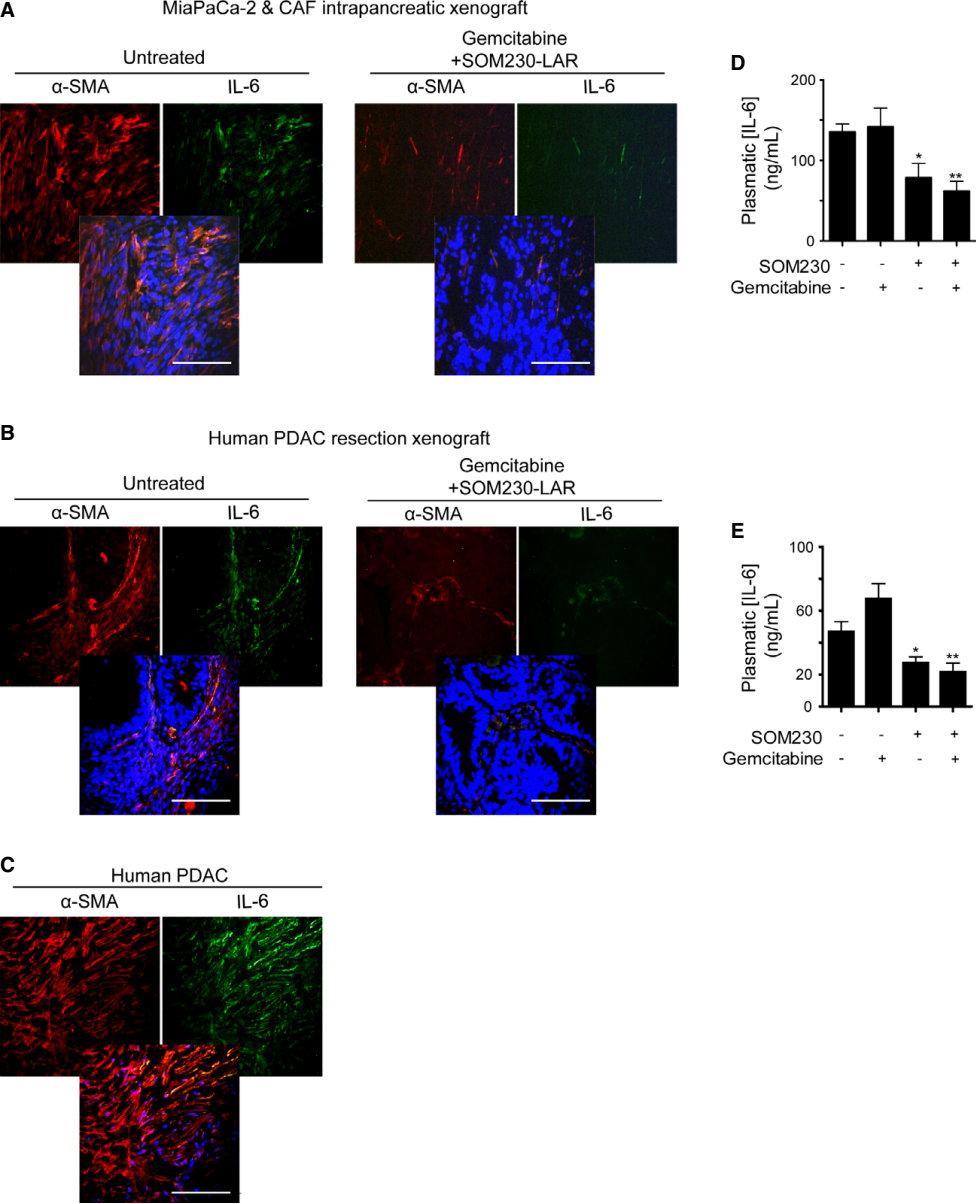
IL-6 concentration correlated with α-SMA-expressing CAFs abundance in human and mouse pancreatic tumours and plasma

We then assessed the status of IL-6 in pancreatic tumours in correlation with the presence of α-SMA-expressing CAFs. To monitor IL-6 expression in a dynamic model of spontaneous pancreatic tumorigenesis, we first used the mouse knock-in model where the mutated Kras was specifically expressed in the exocrine pancreas (Pdx1-Cre; Kras^G12D/+^, named KC) and recapitulated human pancreatic tumorigenesis, including the appearance of precancerous lesions (Hingorani *et al*, 2003). Consistently, the plasmatic IL-6 concentration was significantly increased in KC mice from 6 months of age (Supplementary Fig S12A), where a significant number of precancerous lesions were observed, which further increased at 7 months. This increase correlated with the presence of CAFs expressing α-SMA around precancerous acinar-to-ductal metaplasia and pancreatic intraepithelial neoplasia grade-1 (PanIN1) lesions (Supplementary Fig S12B). In mouse-xenografted experiments using cancer cells and CAF (Fig7A–B, and Supplementary Fig S12C–E), or patient-derived tumours (Fig7B), IL-6 was co-expressed in α-SMA-positive cells. Immunofluorescence analyses of human PDAC sections also showed IL-6 staining in 78% ± 10 of α-SMA-positive fibroblast-like cells (quantified in *n* = 15 PDACs) (Fig7C, Supplementary Table S2D). IL-6 expression was almost undetectable in tumours obtained from mice treated with gemcitabine + SOM230 bi-therapy, in correlation with the reduction in the α-SMA staining (Fig7A–B, Supplementary S12C–G). Importantly, plasmatic human IL-6 concentrations dramatically decreased upon mouse treatment with gemcitabine + SOM230 bi-therapy in both orthotopic xenografted models (cancer cell and CAF, Fig7D, and patient-derived tumours, Fig7E), whereas mouse IL-6 plasmatic concentrations were comparatively very low and not affected (not shown). SOM230 alone also efficiently decreased human IL-6 plasmatic concentrations in the mouse models (Fig7D–E). These data confirm that IL-6 is over-expressed and secreted by human intra-tumour CAFs during the process of pancreatic carcinogenesis, and that IL-6 secretion by CAFs is dramatically reduced by SOM230, thus providing a mechanism for SOM230 re-sensitization of pancreatic cancer cells towards gemcitabine cytotoxicity *in vivo*.

A–C Immunohistofluorescence using an anti-α-SMA or anti-IL-6 antibody in paraffin-embedded sections from MIA PaCa-2-Gluc and CAF cells orthotopic co-xenografted tumours from Fig5A (A) or from human tumour subcutaneously xenografted from Fig5D (B) in nude mice treated or not with gemcitabine + SOM230 (representative of five different tumours). Representative co-localization of α-SMA with IL-6 in human PDAC samples (C) (*n* = 15). Scale bar = 100 μm.

D, E Plasma was collected from MIA PaCa-2GLuc and CAFs (*P = 0.034, **P = 0.007) (D), or human pancreatic tumour (*P = 0.028, **P = 0.004) (E), xenografts in nude mice and human IL-6 plasmatic concentrations (mean ± SD) were quantified by ELISA (*n* = 5 mice / group). * treated versus untreated mice.

## Discussion

**Figure 8:**
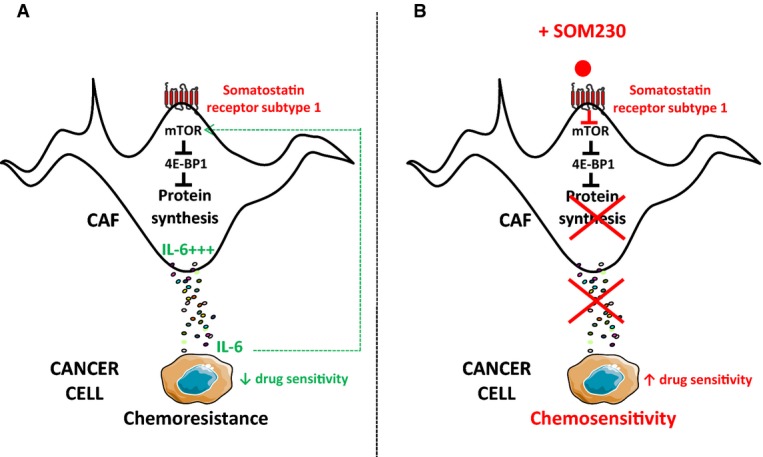
Model

Our data demonstrate that strategies aimed at targeting protein synthesis through inhibition of the mTORC1 pathway in CAFs abolish the chemoprotective effect provided by CAF secretome. Synthesis of both insoluble (e.g. collagen I) and soluble (e.g. IL-6) proteins secreted by CAFs is turned off upon inhibition of mRNA translation, providing a novel strategy to prevent CAF-mediated drug resistance in cancer cells. Interestingly, pharmacotherapy using the multi-receptor somatostatin analogue SOM230 (Pasireotide® Novartis) is introduced here as a promising approach to inhibit protein synthesis specifically in CAFs that express the somatostatin receptor subtype 1, in contrast to their inactive PaSC counterparts (Fig8).

Through the synthesis/secretion of soluble chemoprotective factors including IL-6, CAFs induce pancreatic cancer cell chemoresistance. High protein synthesis rate in CAFs is dependent on the robust intrinsic activation of the mTOR pathway resulting in inhibition of the protein translation inhibitor 4E-BP1. Secreted IL-6 participates in an autocrine feed-forward loop in the intrinsic activation of mTOR and subsequent high protein synthesis.

CAFs express the somatostatin receptor sst1. Upon activation with the novel SOM230 somatostatin analogue (Pasireotide® Novartis) presenting a high affinity for sst1, the mTOR pathway is inhibited, resulting in reduced synthesis/secretion of chemoprotective factors including IL-6 through inhibition of mRNA translation. As consequences, the autocrine feed-forward IL-6/mTOR/protein synthesis/IL-6 loop is abrogated, and pancreatic cancer cells are re-sensitized to the apoptotic action of chemotherapies.

Three different xenografting procedures, that is orthotopic (intrapancreatic) or subcutaneous co-xenografting of human pancreatic cancer cells and human CAF primary cell cultures, and subcutaneous xenografting of human pancreatic tumours (PDX, patient-derived tumour xenografting), were undertaken in immunodeficient mice. These three strategies reproducibly demonstrated that combining the SOM230 treatment with the chemotherapeutic drug gemcitabine provided a therapeutic benefit over each single treatment. Through its indirect action on CAFs, SOM230 enabled the re-sensitization of chemoresistant pancreatic cancer cells to gemcitabine cytotoxicity. In these three models, our results demonstrate that the fibrotic stroma, and subsequent pancreatic cancer cell chemoprotection, provided by the presence of CAFs, mimicked that which normally occurs in human tumours, in contrast to xenografted models which only have pancreatic cancer cells (Perez-Mancera *et al*, 2012). However, the therapeutic benefit provided by our drug association will have to be validated on immune-competent model(s) of PDAC, especially as CAFs also impact on immune cell function (Mace *et al*, 2013). Nonetheless, our results strongly support protein synthesis as a novel therapeutic target in human CAFs.

Somatostatin is the natural neuropeptide produced in the pancreas by the δ islet cells. We have previously reported that somatostatin inhibits the PI3K/mTOR pathway and thereby protein translation at two levels: firstly, it directly inhibits PI3K activity (Bousquet *et al*, 2006; Najib *et al*, 2012), and secondly, it upregulates the expression of the hypophosphorylated form of the translation inhibitor 4E-BP1 (Azar *et al*, 2008; Laval *et al*, 2014). Somatostatin analogues (e.g. octreotide) have been FDA-approved and safely used for decades in the diagnosis (octreoscan) and treatment of secretory syndrome and growth of neuroendocrine tumours including pancreatic neuroendocrine tumours that highly express one or several of the five G protein-coupled somatostatin receptors (sst1, 2, 3 & 5) (Bousquet *et al*, 2012; Chalabi *et al*, 2014). However, octreotide, which chiefly targets sst2, has shown no clinical benefit for PDAC patients, which is probably explained by the absence of expression of sst2 in pancreatic cancer cells (Friess *et al*, 1993; Buscail *et al*, 1996; Laklai *et al*, 2009), or in CAFs, as demonstrated here (Supplementary Fig S5). Conversely, the new generation of somatostatin analogues including SOM230 which also targets the sst1 receptor subtype is shown here to be a very promising drug for the inhibition of CAF secretory activity, thereby re-sensitizing pancreatic cancer cells to chemotherapeutic drugs by abolishing CAF-mediated drug resistance. SOM230 is an FDA-approved drug (in 2012, for the treatment of Cushing's pituitary tumours) that, despite inducing hyperglycaemia, does not generate any toxicity (Schmid, 2008; Chan *et al*, 2012; Henry *et al*, 2013), unlike translation mTOR inhibitors such as RAD001 (Everolimus® Novartis). SOM230 has a high affinity (nanomolar ranges) for all somatostatin receptors, except sst4 (Schmid, 2008). However, the SOM230 inhibitory effect in CAFs was shown to be specifically mediated through sst1, as demonstrated through sst1 expression knock-down (RNA interference) studies. The mechanisms underlying the high expression of sst1, and elevated PI3K/mTORC1 pathway activation in CAFs, particularly when compared to inactivated α-SMA-negative PaSCs, are currently under investigation. Importantly, CAF treatment with SOM230 does not affect sst1 expression levels, which is a prerequisite for the prospective use of this drug for the long-term treatment of PDAC patients. This result however indicates that SOM230 does not inhibit the translation of all mRNAs in CAFs.

Nothing is known about protein synthesis and its regulation in CAFs. Initiation of mRNA translation, the rate-limiting step of protein synthesis, is tightly regulated by signalling pathways that are involved in cancer development and progression, including the PI3K/mTORC1 and eIF2α pathways (Lasfargues *et al*, 2012; Martineau *et al*, 2013). mRNA translation is upregulated in cancer cells where increased cell division and growth require elevated protein synthesis coupled with more ribosomes. Half of human PDACs present pancreatic cancer cells which have lost expression of the negative regulator of translation 4E-BP1, rendering them insensitive to mTOR inhibitors (Martineau *et al*, 2014). Therefore, CAFs represent an additional promising target for therapeutic inhibition of protein synthesis. A significant proportion of CAFs in PDACs present with an activated mTOR pathway (detection of phospho-4E-BP1 and phospho-S6) suggesting a high protein synthesis rate. The translation initiation machinery, capable of mRNA unwinding, is necessary for the specific translation of a subset of mRNAs that have highly structured 5′ untranslated regions (5′UTRs). Those mRNAs encode proteins involved in cell cycle progression, angiogenesis, cell growth and survival functions. Among the soluble proteins that we found to be highly secreted by CAFs, IL-6 was identified as being a mediator of a significant proportion of CAF chemoprotective features in pancreatic cancer cells. Consistently, IL-6 emerges as a potential therapeutic target in PDACs (Lesina *et al*, 2014) and IL-6 serum concentrations in patients with advanced PDAC can predict the efficacy of gemcitabine treatment (Mitsunaga *et al*, 2013). Our findings, which demonstrate that IL-6 expression is regulated through increased protein translation in CAFs, provide an interesting approach to target the high production of this cytokine in PDAC. Consistently, high IL-6 synthesis by CAFs, which is also measurable in the plasma of our tumour-xenografted mouse models, is abrogated upon mouse treatment with SOM230, in correlation with enhanced tumour sensitivity to gemcitabine.

Therapeutic strategies aimed at symptomatically targeting the PDAC pro-tumoural fibrotic scaffold using non-selective enzymes that degrade it (Provenzano *et al*, 2012; Jacobetz *et al*, 2013), CD-40-educated macrophages that alter tumour stroma (Beatty *et al*, 2011), or nanoparticle albumin-bound chemotherapy (Von Hoff *et al*, 2011), showed promising results in pre-clinical models because they led to better chemotherapy delivery and uptake through stromal depletion and enhanced vascularization (Heinemann *et al*, 2014). Several clinical trials in which various forms of antifibrotic therapies are applied concomitantly with gemcitabine are now recruiting patients. Caution has however to be taken regarding these non-selective antifibrotic therapies since breaking down the stromal wall may increase dissemination of cancer cells (Erkan, 2013). Other options to target CAFs in PDAC consist of treatments which reduce CAF proliferation, including hedgehog pathway inhibitors, retinoic acid or pirfenidone which reduce stroma abundance in PDAC models (Olive *et al*, 2009; Froeling *et al*, 2011; Kozono *et al*, 2013), or vitamin D receptor activation which reprogrammes CAFs to a quiescent phenotype (Sherman *et al*, 2014). Although very promising in initial preclinical studies (Olive *et al*, 2009), targeting the proliferation of PDAC-associated fibroblasts using hedgehog pathway inhibitors has failed in phase II trials (Amakye *et al*, 2013). Shh genetic deletion, or chronic treatment with a hedgehog inhibitor, in mice presenting with an intra-pancreatic mutation of Kras and p53, accelerated pancreatic tumour progression and reduced survival, with tumours presenting a poorly differentiated histology and increased vascularity (Rhim *et al*, 2014). Recent findings report that depending on the dosage of hedgehog signalling in the tumour (high, low/absent or only decreased by inhibitors), tumour growth is accelerated, arrested or enhanced, respectively, through increased angiogenesis, probably explaining the unexpected non-efficacy of hedgehog inhibitors in patients (Mathew *et al*, 2014).

SOM230 does not have any direct inhibitory effect on pancreatic cancer cell features *in vitro* (proliferation, migration or invasion) (data not shown). No expression of sst1 was observed in any of the five pancreatic cancer cell lines (Panc-1, BxPC-3, Capan-1, MIA PaCa-2, CFPAC-1), or in pancreatic cancer cells from forty-two human PDACs, as assessed by Western blot or immunofluorescence analyses, respectively, using an anti-sst1 antibody, the specificity of which had been validated here. In orthotopic co-xenograft cancer cell + CAF and PDX (patient-derived tumour sub-cutaneous xenograft) experiments, SOM230 treatment alone diminished CAF activation but was not able to affect cancer cell proliferation or apoptosis. In PDX experiments, SOM230 significantly decreased tumour progression, probably due to a reduction in the rate of collagen deposition by CAFs (Masson's trichrome staining). Therefore, our results show that the inhibitory effect of SOM230 on CAF secretions is insufficient to affect tumour growth, but is sufficient to provide potent chemosensitization, bypassing pancreatic cancer cell resistance to gemcitabine. Our hypothesis for these differences is that pancreatic cancer cells may be able to adapt *in vivo* to the decrease in CAF-derived growth signals induced by SOM230 treatment, but not to the absence of CAF-derived chemoprotective factors. Therefore, these results emphasize the role of CAFs as critical chemoprotective cell partners to pancreatic cancer cells.

A recent paper has unexpectedly demonstrated, in a PDAC transgenic mouse model, that depletion of α-SMA-positive and proliferating cells accelerates cancer progression, instead of stopping it, by inducing immunosuppression (Ozdemir *et al*, 2014). However, the study method employed reportedly targeted all α-SMA-positive cells, including the smooth muscle cells lining vessels and whose function during tumour progression may have been impacted by this strategy. Additionally, only proliferating α-SMA-positive cells were targeted, which probably represent only a sub-population of CAFs, and not necessarily the sub-population that exhibits the highest rate of secretion. Our observations rather show that most human CAFs (present in human PDACs) proliferate slowly *in vitro* and *in vivo* (Supplementary Fig S1B–C). Whether there is a difference of proliferation between human and mouse CAFs present in human pancreatic tumours and in PDAC mouse models, respectively, will have to be explored.

Our results should pave the way for the development of novel drugs that specifically target protein synthesis, not only in cancer cells but also in CAFs. Rational clinical trials using these inhibitors should be designed with the aim of boosting the cytotoxic action of chemotherapy in PDAC. Such treatments would be of major benefit to large groups of both surgical and non-surgical PDAC patients, since part of the activated stroma can remain after tumour removal and may be involved in tumour recurrence (Erkan *et al*, 2012b). Results from phase II trials using mTOR inhibitors in advanced PDAC patients have been disappointing (Wolpin *et al*, 2009; Javle *et al*, 2010). However, inhibition of mTOR induces a positive feedback loop on PI3K and an associated PI3K inhibitor has been used to improve therapy efficacy (Javle *et al*, 2010). Our results indicate that CAFs can be efficiently targeted with inhibitors that target the mTOR pathway, including SOM230 pharmacotherapy which presents a safer toxicity profile than mTOR inhibitors. However, to kill cancer cells, a chemotherapy has to be associated with inhibitors of protein synthesis in CAFs. These novel drug associations that target the heterogeneous pancreatic tumour, including SOM230-LAR, or novel protein synthesis inhibitors, together with a chemotherapeutic drug should provide promising therapeutic benefits for PDAC patients.

## Materials and Methods

### Pancreatic ductal adenocarcinoma samples

Human normal (from healthy donors) and tumour (from patients with surgery-resected pancreatic cancer) paraffin-embedded samples were from the Pathology Department of Toulouse and Limoges Hospitals, France. This study was approved by the ethic committee of the Institutions. Patients’ samples were obtained after getting informed consent in accordance with the Declaration of Helsinki and stored at the ‘CRB cancer des Hôpitaux de Toulouse’ collection. According to the French law, CRB cancer collection has been declared to the ministry of higher education and research (DC 2009-989; DC-2011-1388) and obtained a transfer agreement (AC-2008-820; AC-2011-130) after approbation by ethical committees. Clinical and biological annotations of the samples have been declared to the CNIL (Comité National Informatique et Libertés).

### Human cancer-associated fibroblast isolation and cell culture

CAFs were isolated from human pancreatic tumour tissues using the outgrowth method described by Bachem *et al* (Bachem *et al*, 1998). Commercial human primary cultures of PaSCs are isolated from human pancreas (reference 3830, lot # 10295 and lot # 10473, ScienCell Research Laboratories). CAFS and PaSC were grown in Dulbecco's modified Eagle's medium F12 (DMEM/F12 Lonza) containing 10% foetal calf de-complemented serum (FCS, Life Technologies).

### CAF conditioned media

CAF conditioned media were made from the culture of 10^6^ CAFs in 5 ml of DMEM/F12 without FCS, and treated or not with 10^−7^ M SOM230. Forty-eight hours later, conditioned media were collected, centrifuged (1,000 *g*, 5 min) and filtered (0.2 μm) prior to incubation with cancer cells. When indicated, conditioned media from untreated CAFs were pre-treated for 30 min with 1 ng/ml of human IL-6 neutralizing IgA monoclonal antibody (Invivogen) prior to incubation with cancer cells.

### Membrane antibody array

Soluble factors present in PaSC and CAF conditioned media were identified and quantitatively compared (SOM230-treated versus SOM230-untreated CAFs) using Human Cytokine Antibody Array 5 (AAH-CYT-5-8, RayBiotech). This antibody array matrix can simultaneously detect 80 cytokines, chemokines and growth factors. Briefly, CM were collected, filtered, volume-normalized according to cells number and incubated with the antibody arrays overnight at 4°C. Membranes were processed according to the manufacturer's instructions. The spot density was quantified by using a ChemiDoc™ XRS System with Image Lab™ Software (Bio-Rad).

### Tumour growth in nude mice

#### Pancreatic cancer cells and CAF co-xenografting

Pancreatic cancer cells and CAFs were trypsinized, washed and resuspended in sterile PBS. A 1:3 mix of pancreatic cancer cells (10^6^) and CAFs (3 × 10^6^) were subcutaneously (Panc-1 cells) or orthotopically (intra-pancreatic) (MIA PaCa-2-GLuc cells) injected in 100 or 50 μl PBS, respectively, of 4-week-old female nude mice (NMRI-nu/nu, NMRI-Foxn1nu Janvier) (Sicard *et al*, 2013). Mice were anesthetized by inhalation of isoflurane. Treatments started 1 week after grafting when mice have been randomized in the four group treatments, with similar mean tumour volumes per group (for subcutaneous grafting experiment, 33–71 mm^3^) or similar mean plasmatic luciferase activity per group (for orthotopic grafting experiment, 181- to 196-fold increase as compared to activity measured at grafting day 0).

#### Patient-derived tumour xenografting

The tumour xenografts are derived from a surgical resection of a pancreatic tumour (moderately differentiated, stage IIA). Following excision at surgery, tumour pieces are subcutaneously implanted into nude mice. Tumour fragments are obtained from xenografts in serial passage in 4-week-old female nude mice (NMRI-nu/nu mice, NMRI-Foxn1nu) (*n* = 7, until establishment of a stable growth pattern). After removal from donor mice, tumours are cut into fragments (3–4 mm edge length) and placed in PBS. Recipient animals are anesthetized by inhalation of isoflurane and receive tumour implants subcutaneously in the flank. Forty-one days following tumour implantation, animals have then been randomized (= day 0) into experimental groups with equivalent mean group tumour volumes (100–120 mm^3^).

#### Treatments

Mice have been treated s.c. with SOM-LAR (80 mg/kg) once every 3 weeks and/or i.p. with gemcitabine (100 mg/kg, twice per week).

Tumour volumes of subcutaneous tumours were calculated as 0.523 × l^2^ × L.

All experiments were done in accordance with the principles and guidelines established by INSERM Anexplo UMS006 and were approved by the institutional and national animal care and use committees.

### Statistical analyses

Statistical analyses were performed by comparing two by two independent conditions (with homogeneous variances) using an unpaired parametric *t*-test. All values are mean ± SD of independent *n* experiments (as indicated). Differences were considered statistically significant when *P* < 0.05 (*P < 0.05; **P < 0.01; ***P < 0.001).

### For more information

Author's website: http://www.crct-inserm.fr

**The paper explained**

**Problem**

While the cancer-associated fibroblasts (CAFs) secretome is known to contribute to tumour chemoresistance, its therapeutical targeting for chemosensitization remains elusive. This study addresses the hypothesis that a pharmacotherapy aimed at inhibiting protein synthesis/secretion can reverse chemoprotection provided by CAFs.

**Results**

We developed *in vitro* and *in vivo* co-culture models where CAFs were isolated from human pancreatic ductal adenocarcinoma (PDAC) resected tumours and CAF secretome provided complete chemoprotection on pancreatic cancer cells. CAFs displayed elevated mTOR pathway activity and high protein synthesis rates, both *in situ* in human PDACs and *ex vivo* in primary cultures. The mTOR pathway regulates the specific translation of a subset of mRNAs that mainly encode in tumours pro-oncogenic proteins. Chemoprotection induced by CAF secretome dependently on the upregulation in cancer cells of the anti-apoptotic protein survivin was reversed upon mTOR inhibition in CAFs. This can be achieved using the somatostatin analogue SOM230, FDA-approved for the treatment of endocrine tumours, which activates the G protein-coupled somatostatin receptor sst1 selectively expressed in CAFs, but not in inactivated pancreatic stellate or cancer cells. SOM230 exhibited synergistic anti-tumour growth in combination with the chemotherapy gemcitabine in three different xenografting procedures in immunodeficient mice, including PDX (patient-derived xenograft).

**Impact**

Pancreatic cancer adenocarcinoma is a dismal disease resistant to chemotherapies. Our study raises the intriguing possibility that novel drug associations combining current chemotherapies with inhibitors of protein synthesis may be effective in combating tumour chemoresistance.

## Supporting Information

Supplementary Information

Review Process File
