# Supplementary material for: Pharmacological targeting of the protein synthesis mTOR/4E-BP1 pathway in cancer-associated fibroblasts abrogates pancreatic tumour chemoresistance
Source: EMBO Mol Med. 2015 Apr 1;7(6):735–53. doi: 10.15252/emmm.201404346 (PMC4459815; doi:10.15252/emmm.201404346)

Supplementary Informations

Pharmacological targeting of the protein synthesis mTOR/4E-BP1 pathway in cancer-associated fibroblasts abrogates pancreatic tumor chemoresistance

Camille Duluc[1], Siham Moatassim-Billah[1,2], Mounira Chalabi-Dchar[1], Aurélie Perraud[3], Rémi Samain[1], Florence Breibach[4], Marion Gayral[1], Pierre Cordelier[1], Marie-Bernadette Delisle[4], Marie-Pierre Bousquet-Dubouch[5], Richard Tomasini[6], Herbert Schmid[7], Muriel Mathonnet[3], Stéphane Pyronnet[1], Yvan Martineau[1], Corinne Bousquet[1,8]

Affiliations:

[1]INSERM UMR-1037, Université de Toulouse, Cancer Research Center of Toulouse (CRCT), Equipe labellisée Ligue Contre le Cancer and Laboratoire d'Excellence Toulouse Cancer (TOUCAN), Toulouse, France.

[2]Biochemistry-Immunology Laboratory, Faculty of Sciences Rabat, University Mohammed V - Agdal, Morocco.

[3]Limoges University, EA 3842 Laboratory, Medicine and Pharmacy Faculties, Limoges, France.

[4]Hôpitaux de Toulouse, Pathology Department, Toulouse, France.

[5]CNRS UMR-5089, Université de Toulouse, Institut de Pharmacologie et de Biologie structurale (IPBS), Toulouse, France.

[6]CRCM, INSERM, U1068; Paoli-Calmettes Institute; Aix-Marseille University, UM105; CNRS, UMR7258, Marseille, France.

[7]Novartis Pharmaceuticals, Bales, Switzerland.

[8]To whom correspondence should be addressed: Corinne Bousquet, INSERM UMR-1037, Centre de Recherches en Cancérologie de Toulouse (CRCT), 2 avenue Hubert Curien, Oncopôle Entrée C, CS53717, 31037 Toulouse cedex1, France, Tel : +33-5-82-74-16-53, E-mail : corinne.bousquet@inserm.fr.

Keywords: Pancreatic cancer, cancer-associated fibroblasts, chemoresistance, pharmacotherapy, protein synthesis & secretion

Disclosures: Authors have nothing to disclose

Material and Methods

Cell lines, culture and transfection

Ductal pancreatic adenocarcinoma cell lines are grown in RPMI (Panc-1), DMEM (4,5 g glucose, Lonza) (BxPC-3, Mia PaCa-2 and Capan-1), and Iscove's Modified Dulbecco's Medium (CFPAC-1), containing 10% FCS. Cells were cultured at 37°C, 5% CO2 in a humid atmosphere. All media (Lonza) were supplemented with antibiotics (5 IU / mL penicillin and streptomycin, 2.5 mg / mL of fungizon and 2.5 µL / mL plasmocin) and L-glutamine (2 mM) (Life Technologies). Mia PaCa-2-GLuc cells expressing the secreted Gaussia Luciferase were obtained by transducing the Gaussia Luciferase lentiviral vector (pLentiCMV-GLuc-IRES-EGFP, TargetingSystems, CA USA). Briefly, 5.10^4 Mia PaCa-2 cells were plated in 48-well plates and transduced overnight with the lentiviral vector at the multiplicity of infection of 5 in 250 µL of transduction medium (complete culture medium + 4 µg / mL Protamine sulfate), grown and selected for efficient transduction with the lentivector using neomycin selection (200 µg / mL). Efficient Gaussia Luciferase (GLuc) production and secretion in Mia PaCa-2-GLuc cells was checked by measuring its activity in 5 µL of culture medium using coelenterazine (0.5 µM) as a substrate.

Reagents

Antibodies used are as follows: α-SMA (Epitomics), Vimentin (Abcam); sst1 (generated in Novartis Pharma laboratory (Basel, Switzerland) (Lambertini et al, 2013; Schmid et al, 2012); GAPDH, SHP-2 and PTPε (Santa Cruz); β-Actin (Sigma-Aldrich); P-p70S6K (T389), P-Akt (S473), Akt, P-4E-BP1 (S65), 4E-BP1, XIAP, survivin, cIAP1, cIAP2, livin (Cell Signaling); Puromycin (Millipore); IL-6, Ki67 (Abcam). Drugs used are as follows: SOM230 and octreotide (10^{-7}M and 10^{-8}M, Novartis Pharma, Basel, Switzerland); RAD001 (1 µM, Calbiochem) and PP242 (10 µM, Sigma) mTOR inhibitors; survivin antisens oligonucleotide (5 µM, Calbiochem), gemcitabine (100 µg / mL, Mylan Pharma), 5-fluorouracil (100 µg / mL, Sigma), oxaliplatin (25 µg / mL, Sigma Aldrich), recombinant human IL-6 (1 ng / mL, PreproTech), pertussis toxin (PTX, 100 ng / mL, Sigma Aldrich), gallein (10 µM, R&D Systems), phosphatase inhibitor (NSC87877, 10 µM, R&D Systems), PDGFR inhibitor (5

or 10 µM, AG1296, Abcam), Recombinant PDGF (5µg/mL, R&D Systems), EGFR inhibitor (AG1478, 150 or 300 nM, Sigma Aldrich), Jak1/2 inhibitor (Ruxolitinib, 5 or 10 µM, Selleckchem), Src inhibitor (PP1, 5 µM, Calbiochem).

Immunofluorescence

CAFs and PaSCs were fixed with 4% paraformaldehyde for 20 min at 4°C. Serial sections of formalin-fixed, paraffin-embedded human tumor samples and xenografts were used. Selected proteins were assessed using the indicated antibodies. Reactions were revealed with the following fluorescent secondary antibodies: donkey anti-mouse Alexa 488 (molecular probes) and goat anti-rabbit Cy3 (Jackson ImmunoResearch), and cell nuclei stained with DAPI. Images were captured by confocal microscopy (Zeiss LSM 510). Quantifications were performed using the Image J software.

Immunohistochemistry

Serial sections of formalin-fixed, paraffin-embedded human tumor samples or xenografts were incubated with the indicated antibodies. Reactions were amplified with ImmPRESS Peroxidase Polymer Detection Reagents (VECTOR Laboratories). A solution 3-amino-9-ethylcarbazole (AEC) or 3,3-Diaminobenzidine (DAB) were used as chromogen and sections were counterstained with hematoxylin. Trichrome (Masson's) stainings were carried out according to the manufacturer's instructions (Sigma-Aldrich). Images were captured using an Eclipse E400 microscope (Nikon) and Explora Nova Morpho Expert/Mosaic Software. Quantifications were performed using the Image J software.

MTT Assay

Tumor cells were seeded in 96-well plates (20 000 cells/well). After adhesion, cells were washed and starved. The following day, cells were treated with gemcitabine and stimulated or not with CAF conditioned media as indicated. MTT, (4,5-dimethylthiazol-2-yl)-2,5-diphenyl tetrazolium (Life Technologies) was added to each well at 0.5 mg/mL for 2 hours. Hundred µl of dimethylsulfoxide

were added for 1 h to each well. Viability was estimated by measuring absorbance at 570 nm on MRX plate reader (Dynex Technologies).

Flow cytometry

Cells were seeded in flat-bottomed 6-well plates (0.5×10^6 / well). After 12 h, cells were washed and starved during 18 h. Medium was replaced with CAF conditioned medium for 12 h. Gemcitabine was added to CAF conditioned media and cells were incubated at 37°C for additional 24 h. Phosphatidylserine externalization was evaluated by flow cytometry after cell labeling with Annexin V-FITC (250 ng/mL) and propidium iodide (12.5 µg/mL; AbCys) using a FACScan (BD Biosciences) cytometer.

DEVD cleavage enzyme assay

Cell lysates were incubated for 30 min with Ac-Asp-Glu-Val-Asp-aminomethylcoumarin (Alexis). Amount of the released fluorescent product was determined by spectrometry at 351 and 430 nm for the excitation and emission wavelengths, respectively.

ELISA assay

Levels of IL-6 were quantified by using an enzyme-linked immunosorbent assay (ELISA; Peprotech) according to manufacturer's instructions. Briefly, all standards and samples were incubated in duplicate with human or mouse IL-6 antibody coated on a 96-well plate overnight at 4°C. The wells are washed and biotinylated antibody human or mouse anti-IL-6 antibody is added, respectively. After washing away unbound antibody, HRP-conjugated streptavidin is pipetted to the wells. A TMB substrate solution is added and color develops in proportion to the amount of IL-6 bound. The intensity of the color is then measured at 450nm on MRX plate reader (Dynex Technologies).

Soluble and insoluble Collagen assays

Levels of soluble and insoluble collagen produced by CAF were quantified by using collagen assays according to manufacturer's instructions (Quickzyme Biosciences). The intensity of the color

associated to the hydroxyproline content is measured at 570nm on MRX plate reader (Dynex Technologies).

Western blot

Cells were lysed in lysis buffer (20 mM Tris, pH 7.5, 150 mM NaCl, 1 mM EDTA, 1% NP40, 1 mM sodium orthovanadate, 1 mM NaF and a cocktail of protease inhibitors, Roche). Protein extract concentration was measured using Protein Assay reagent (Bio-Rad), and equal amounts of proteins were resolved by SDS-PAGE and electroblotted onto nitrocellulose membranes. Membranes were blocked in 5% powdered milk in Tris-buffered saline with 0.1% Tween 20 (TBST) for 1 h followed by incubation with primary antibodies overnight at 4°C. Membranes were then washed with TBST three times and incubated with horseradish peroxidase-coupled secondary antibody for 1 h at room temperature, washed again, and treated with enhanced chemiluminescence prior to detectionon x-ray film.

SUnSET assay

SUnSET assay was used to monitor the rate of protein synthesis. Briefly, 10 min prior cell harvesting, puromycin was added to culture medium at 1 µg/ml. CAF extracts were then processed for Western blotting using anti-puromycin antibody {Schmidt, 2009 #3437}.

CAF polysomes profiling

CAFs were seeded at 2 x 10^6 cells / plate. After 12 h, cells were washed, starved during 18 h and treated with SOM230 (10^{-7}M) for 2 days. Total RNA was extracted from CAF treated with cycloheximide (100 µg/mL, Sigma) for 15 min and separated on 10–50% sucrose gradient with centrifugation at 35000 rpm. Polysome profiles were determined by measuring absorbance at 254 nm.

Real-time quantitative RT-PCR

Total RNA was extracted with RNeasy kit (Qiagen) and equal amounts of RNAs were reverse-transcribed using Superscript One Step RT-PCR kit (Invitrogen®) and resulting cDNAs were used in

qRT-PCR using SYBR green (Applied Biosystems) according to the manufacturer's instructions. Primer sequences (Invitrogen) were as follows: SSTR1 sense 5'-CAC ATT TCT CAT GGG CTT CCT-3', reverse 5'-ACA AAC ACC ATC ACC ACC ATC 3' ; SSTR2 sense 5' –GGC ATG TTT GAC TTT GTG GTG -3', reverse 5'- GTC TCA TTC AGC CGG GAT TT -3'; SSTR3 sense 5'- TGC CTT CTT TGG GCT CTA CTT -3', reverse 5' - ATC CTC CTC CTC AGT CTT CTC C -3'; SSTR4 sense 5'- CGT GGT CGT CTT TGT GCT CT -3', reverse 5'-AAG GAT CGG CGG AAG TTG T -3'; SSTR5 sense 5'- CTG GTG TTT GCG GGA TGT T -3', reverse 5'- GAA GCT CTG GCG GAA GTT GT -3'; IL-6 sense 5'- CTG ACC CAA CCA CAA ATG CC -3', reverse 5'- GGT TCT GTG CCT GCA GCT TC 3'; GRO alpha sense 5′-TCG CTT CTC TGT GCA GCG CT-3′, reverse 5′-GTG GTT GAC ACT TAG TGG TCT C-3′; and MCP-1 sense 5'-CCC CAG TCA CCT GCT GTT AT, reverse 5'-TGG AAT CCT GAA CCC ACT TC-3'. Target gene expression was normalized by using HPRT expression as an internal control.

RNA interference

hCAFs were transfected with siRNA targeting SSTR1 (SMARTpools, Dharmacon, 100 nM), or PTPN11/SHP2 (SMARTpools, Dharmacon, 100 nM), or PTPε (SMARTpools, Dharmacon, 100 nM) or 4E-BP1 (Applied Biosystems, 50 nM, forward 50-CAAGAACGAACCCUUCCUU-3' and reverse) using DharmaFECT1 reagent (Dharmacon) or siPort NeoFx transfection reagent (Applied Biosystems), respectively, according to the manufacturer's instructions.

TUNEL

Slides are deparaffinized (xylene) and rehydrated (ethanol, PBS 1X). TUNEL staining is performed using the proTUNEL-IHC DNA Fragmentation Assay Kit (Euromedex), according to the manufacturer's instructions. Briefly, slides are first incubated with the permeabilization buffer (Proteinase K, 20 min at room temperature) and the endogenous peroxidase activity was blocked with 0.3% hydrogen peroxide in methanol. Then, slides are incubated with the TUNEL (terminal deoxynucleotidyltransferase [TdT]-mediated dUTP-biotin nick end labeling) reaction mixture (60 min, 37°C), followed by the detection step with the streptavidin / horseradish-peroxidase complex (60 min),

and then with a diaminobenzidine chromogen solution (1 min), and counterstained with Methyl Green. Slides stained with no TUNEL mixture were used as negative controls.

Table and Figure Legends

Table S1. List of PDAC human tumors used for CAF isolation.

Table S2. Quantifications (Image J) of the % of sst1- and α-SMA- positive cells **(A)**, or of sst1- and P-4E-BP1- positive cells **(B)**, or of α-SMA- and P-4E-BP1 -positive cells (**C**), or α-SMA- and IL-6 -positive cells **(D)**, performed in 3 different fields / tumour, on the indicated number of human PDACs.

Table S3. Exhaustive list of cytokines / chemokines present and quantified (Image J) on the membrane antibody array (Human Cytokine Antibody Array 5) blotted with CAF-CM. In gray are highlighted the factors whose expression is significantly downregulated in SOM230-treated CAF-CM.

Figure S1. Differential features of PaSCs vs. CAFs from human normal or tumoral pancreas.
(A) Hemalun-Eosin and Trichrome of Masson colorations, or immunohistochemistry using an anti- α-SMA antibody, in paraffin-embedded normal (upper panels) and tumoral pancreatic sections (lower panels) (representative of 3 different normal or PDAC samples). For each stain / coloration, regions in white square (low magnification) have been zoomed in the corresponding lower panel (high magnification). Scale bars are 100 μm. **(B)** Comparative time-course proliferation of CAFs and PaSCs (n=3) (From left to right, p=0.003, p=0.02, p=0.005, p=0.002, p=0.004). **(C)** Immunofluorescence confocal imaging of human pancreatic tumor using an anti- α-SMA and Ki67 antibody (one representative field of n=12 tumors). **(D)** Protein concentrations measured (Bradford method) in conditioned media (CM) from CAFs and PaSC. Results are normalized to cell number and presented as the percentage of CAFs (= 100%) (n=3) (p=0.007). *: PaSC vs. CAFs.

Figure S2. CAFs secrete pro-survival factors leading to pancreatic cancer cell resistance to the chemotherapeutic drugs 5FU and oxaliplatin.
(A-F) Pancreatic cancer Panc-1 cell viability was assessed by MTT. Panc-1, incubated in the presence of 5FU (100 μg/mL) (From left to right, p=0.003, p=0.002) **(A)** or of oxaliplatin (25 μg/mL) (From

left to right, p=0.003, p=0.033) **(B)**, were stimulated with CAF- or PaSC-CM (n=3); Panc-1, incubated in the presence of gemcitabine (100 µg/mL), were stimulated with CAF-CM previously boiled at 95°C (n=3) (From left to right, p=0.043, p=0.049) **(C)**; Panc-1, incubated in the presence of gemcitabine (100 µg/mL), were stimulated with CM from untreated or SOM230-treated CAFs with the indicated concentrations (n=3) (From left to right, p=0.027, p=0.031, p=0.022, p=0.021) **(D)**; Panc-1 were incubated in the presence of 5FU (100 µg/mL) (From left to right, p=0.007, p=0.027, p=0.008) **(E)** or of oxaliplatin (25 µg/mL) (From left to right, p=0.003, p=0.046, p=0.002) **(F)** with CM from untreated or SOM230 (10^{-7}M) -treated CAFs (n=3) **(E, F)**. Results are presented for each treatment as the percentage of the respective gemcitabine-, 5FU-, or oxaliplatin- untreated cells (=100%) (n=3). **(G, H)** Apoptosis induced by 5FU **(G)** or oxaliplatin **(H)** was assessed by Western blot using an anti-cleaved caspase 3 or -GAPDH (loading control) antibody (representative of n=3). *: effect of treatment (gemcitabine or SOM230) vs. untreated cells; #: CM-incubated vs. non-incubated gemcitabine-treated cells.

Figure S3. CAF-CM induces chemoprotection in many different pancreatic cancer cell lines, which is inhibited by CAF pre-treatment with SOM230.

(A, D) Executioner caspase (DEVDase) activity assay in Panc-1 (From left to right, p=0.008, p=0.004, p=0.007) (**A**) and BxPC-3 cells (From left to right, p=0.026, p=0.032, p=0.039) (**D**). Results are presented for each treatment (CAFs ± SOM230 CM, SOM230) as the fold of the gemcitabine-untreated cells (=1) (n=3). **(B, C)** Cell viability was assessed by MTT on Capan-1 (From left to right, p=0.005, p=0.002, p=0.004) (**B**) and BxPC-3 (From left to right, p=0.002, p=0.037, p=0.002) (**C**). Results are presented for each treatment (CAFs ± SOM230 CM, SOM230) as the percentage of the respective gemcitabine-untreated cells (=100%) (n=3). **(E, F)** Cell viability of Panc-1 (From left to right, p=0.028, p=0.023, p=0.042) **(E)** and BxPC-3 (From left to right, p=0.025, p=0.009, p=0.033, p=0.041) **(F)** was determined by MTT assay upon treatment with increasing doses of Gemcitabine for 48 h, in the presence or not of conditioned media (CM) from CAFs treated or not with SOM230 (n=3). Corresponding IC50 were calculated **(G)**. *: effect of treatment (gemcitabine or SOM230) vs. untreated cells; #: CM-incubated vs. non-incubated gemcitabine-treated cells.

Figure S4. SOM230 displays a potent inhibitory effect on CAF protein synthesis as mTOR inhibitors

(A) Total RNA levels quantified at 260 nm in untreated or SOM230-treated CAFs (+). Results are presented as the percentage of the untreated cells (=100%) (n=3). **(B)** Immunoblotting of protein extracts from CAFs treated (+) or not with SOM230 (10^{-7}M), RAD001 (20 nM) or PP242 (2,5 µM) for 48h, using an anti-Puromycin antibody (representative of n=3). **(C)** Panc-1 cell viability was assessed by MTT. Panc-1 cells were incubated in the presence of gemcitabine (100 µg/mL) with CM from untreated or SOM230-, RAD001-, or PP242-, treated CAFs. Results are presented as the percentage of the untreated cells (=100%) (n=3) (From left to right, p=0.043, p=0.027, p=0.012, p=0.015, p=0.021). *: effect of treatment (gemcitabine or SOM230 or RAD001 or PP242) vs. untreated cells; #: CM-incubated vs. non-incubated gemcitabine-treated cells.

Figure S5. CAFs express the somatostatin receptor subtype 1 sst1 - Validation of the specificity of the anti-sst1 antibody.

(A, B) Western-blot **(A)** or immunofluorescence confocal microscopy **(B)** analyses using an anti-α-SMA or anti-sst1 antibody in siCTR or sisst1-transfected CAFs (representative of n=3). In **B**, CAFs have been paraffin-embedded. **(C)** Immunofluorescence confocal microscopy analyses of α-SMA co-localization with with P-4E-BP1 in human PDAC serial slides (representative of n=15 PDAC samples).

Figure S6. Octreotide doesn't display an inhibitory effect on CAF chemoprotective features.

(A, B) Executioner caspase (DEVDase) activity assay in Panc-1 (From left to right, p=0.008, p=0.037, p=0.029) (**A**) and BxPC-3 cells (From left to right, p=0.008, p=0.042, p=0.037) (**B**). Results are presented for each treatment (CAFs ± Octreotide CM, Octreotide) as the fold of the gemcitabine-untreated cells (=1) (n=3). **(C, D)** Cell viability was assessed by MTT on Panc-1 (From left to right, p=0.033, p=0.037, p=0.026) (**C**) and BxPC-3 (From left to right, p=0.041, p=0.036, p=0.031) (**D**). Results are presented for each treatment (CAFs ± Octreotide CM, Octreotide) as the percentage of the

respective gemcitabine-untreated cells (=100%) (n=3). **(E)** Apoptosis induced by gemcitabine in Panc-1 was evaluated by Western-blot using an anti-cleaved caspase 3 or -PARP antibody (representative of n=3). *: effect of treatment (gemcitabine or SOM230) vs. untreated cells; #: CM-incubated vs. non-incubated gemcitabine-treated cells.

Figure S7. SOM230 increases *in vivo* sensitivity to gemcitabine of tumor xenograft (Mia PaCa-2-Luc cell & CAF or human PDAC resection).

(A, C, F) MIA PaCa-2-GLuc cells were injected with CAF into the pancreas of nude mice. **(B, D-E, G)** Human tumors were subcutaneously xenografted in nude mice. Mice were treated with each indicated treatment. Tumors were paraffin-embedded for immunohistochemistry analyses using an anti-Ki67 or -αSMA antibody, or Masson's Trichrome coloration. Ki67 (p=0.036) **(D)** or collagen (in Masson's Trichrome coloration) (**C.** p=0.009; **E.** From left to right, p=0.046, p=0.007) **(C-E)** quantifications were performed by counting, in five independent tumors, the mean number of positive cells per field **(D)**, or the % of positive area using the Image J software **(C, E)**. *: Treated vs. untreated xenograft. **(F-G)** Immunofluorescence confocal microscopy analyses of α-SMA (red) and sst1 (green) in tumors. Co-expression of α-SMA and sst1 is shown (merge, yellow) (one representative field of n=3).

Figure S8. SOM230 increases *in vivo* sensitivity to gemcitabine of tumor (Panc-1 cell & CAF) xenograft.

(A-B) Panc-1 cells were injected subcutaneously with or without CAF in nude mice and tumor volume measured (n=5). Tumors were excised, weighted (p=0.006) **(C)** and paraffin-embedded for immunohistochemistry analyses using an anti-Ki67, -cleaved caspase-3, or -αSMA antibody, or Tunel, or Masson's Trichrome coloration **(D)**. **(E)** Tunel (p=0.006), or **(F)** collagen (in Masson's Trichrome coloration) (From left to right p=0.005, p=0.024) and α-SMA (p=0.017) **(G)** quantifications were performed, by counting, in five independent tumours, the mean number of positive cells per field (p=0.007) **(E)** or the % of positive area using the Image J software **(F-G)**. *: Treated vs. untreated xenograft.

Figure S9. CAFs but not PaSC produce collagen - downregulation by SOM230 treatment.

(A) Immunofluorescence imaging on CAFs treated or not with SOM230 (10^{-7}M) using an anti- α-SMA or -collagen I antibody (one representative field of n=3). **(B)** Collagen deposits (indicated with an asterisk) were counted per field (in five field of n=3 independent experiments) (p=0,046). **(C)** Western blot using an anti-collagen I antibody of CM from CAF treated (+) or not with SOM230. Red Ponceau coloration was used as loading control (representative of n=3). **(D, E)** Soluble (From left to right, p=0.036, p=0.045, p=0.005, p=0.029) **(D)** and insoluble (From left to right, p=0.023, p=0.003, p=0.017, p=0.021) **(E)** collagen productions were measured in CAFs and PaSC cell extracts and CM (conditioned media) after 48 h culture (n=3). *: SOM230-treated vs. untreated cells; #: PaSC vs. CAF.

Figure S10. Soluble proteins present in CAF-conditioned media decrease survivin expression in pancreatic cancer cells, resulting in chemoresistance.

(A) Immunoblotting using anti-XIAP, anti-survivin or -GAPDH antibodies, of protein extracts from Panc-1 cells incubated with the CM from CAFs treated or not with SOM230 (representative of n=3). **(B-D)** Immunoblottings, using anti-survivin or -GAPDH (loading control) antibody, of protein extracts from Panc-1 cells incubated with the CM from CAFs treated or not with SOM230 **(B)**, or with the CM from siCTR- or si4E-BP1-transfected CAFs treated or not with SOM230 **(C)**, or with the CM from CAFs in the presence or not of the survivin antisens oligonucleotide (AS) (5 µM) **(D)** (representative of n=3). **(E, F)** Gemcitabine-treated Panc-1 cell viability and apoptosis were assessed by MTT (n=3) (From left to right, p=0.002, p=0.001, p=0.012) **(E)** or Western-blot using the anti-PARP or -GAPDH (loading control) antibody (representative of n=3), in the presence or not of CM from CAFs treated or not with SOM230, and in the presence or not of the survivin antisens oligonucleotide. Results are presented as the percentage of the untreated cells (=100%) (n=3) **(F)**. *: gemcitabine-treated vs. untreated cells; #: CM-incubated vs. non-incubated cells; §: survivin AS-treated vs. untreated cells.

Figure S11. Comparative analyses of CAF and PaSC secretome, and role of IL-6 in the resistance to others chemotherapeutics (5FU and Oxaliplatin).

(A) Membrane antibody array assay using CM from CAF or PaSC (representative of n=3). Controls are circled in blue dashed line, IL-6 in red square, GRO-alpha and MCP-1 in green square. **(B, D, F)** Anti-IL-6 (p=0.037) **(B)**, -MCP-1 (From left to right, p=0.005, p=0.004, p=0.005) **(D)** and -GRO-alpha (From left to right, p=0.006, p=0.004, p=0.005) **(F)** ELISA assays using CM from CAFs or PaSC treated or not with SOM230. **(C, E, G)** Expression of IL-6 **(C)**, MCP-1 (From left to right, p=0.031, p=0.036) **(E)** and GRO-alpha (From left to right, p=0.039, p=0.026) **(G)**, analyzed by RT-qPCR in untreated or SOM230-treated CAFs or PaSC (n=3). **(H)** Immunoblotting using an anti-IL-6 receptor or -GAPDH antibodies of protein extracts from CAFs (n=8), PaSC (n=2) and pancreatic cancer cells (representative of n=3). **(I, J)** Apoptosis induced by 5FU **(I)** or Oxaliplatin **(J)** was evaluated by Western-blot using an anti-cleaved caspase 3 or -PARP antibody (representative of n=3).

Figure S12. Correlation between IL-6 concentration and α-SMA-expressing CAFs in mouse pancreatic tumors and plasma.

(A) Plasma were collected from KC or WT mice at the indicated ages and mouse IL-6 plasmatic concentrations were quantified by ELISA (n=5 mice / group) (From left to right, p=0.0009, p=0.0005). **(B, C)** Immunohistochemistry using an anti- α-SMA or -IL-6 antibody in paraffin-embedded sections from 7 month-old KC (Pdx1-Cre; $Kras^{G12D}$) or wild type (WT) mice **(B)**, or from Panc-1 & CAF cell subcutaneously co-xenografted tumors in nude mice treated or not with gemcitabine ± SOM230-LAR from Fig. S6 **(C)** (representative of 5 different tumors). *: KC vs. WT mice. **(D, E)** Immunohistofluorescence using an anti- α-SMA or -IL-6 antibody in paraffin-embedded sections from MiaPaca-2Gluc & CAF **(D)** or human PDAC tumor resections **(E),** orthotopically xenografted in nude mice treated or not with gemcitabine or SOM230-LAR. **(F, G)** α-SMA quantifications as performed in five independent tumors from D (**F.** From left to right, p=0.007, p=0.007, p=0.005) and E (**G.** From left to right, p=0.004, p=0.005, p=0.0003), respectively.

Duluc *et al*, Table S1

Tumors	Degree of differenciation of cancer cells	Stage
T1	well-differenciated	IIB
T2	moderately differenciated	IIB
T3	moderately differenciated	III
T4	moderately differenciated	III
T5	well-differenciated	IIA
T6	well-differenciated	IB
T7	poorly differentiated	IIB
T8	moderately differenciated	III
T9	poorly differentiated	III
T10	well-differenciated	III
T11	well-differenciated	III
T12	moderately differenciated	IIB
T13	well-differenciated	IB
T14	moderately differenciated	IIB
T15	well-differenciated	IB

Duluc *et al*. Table S2

A

	% sst1+ / α-SMA+
**T1**	43
**T2**	72
**T3**	82
**T4**	46
**T5**	93
**T6**	68
**T7**	63
**T8**	58
**T9**	74
**T10**	93
**T11**	61
**T12**	51
**T13**	66
**T14**	81
**T15**	51
**T16**	63
**T17**	78
**T18**	49
**T19**	88
**T20**	63
**T21**	93
**T22**	79
**T23**	75
**T24**	84
**T25**	65
**T26**	49
**T27**	57
**T28**	48
**T29**	36
**T30**	100
**T31**	71
**T32**	25
**T33**	57
**T34**	37
**T35**	47
**T36**	100
**T37**	81
**T38**	100
**T39**	100
**T40**	80
**T41**	64
**T42**	94

B

	% sst1+ / P-4E-BP1+
**T1**	23
**T2**	46
**T3**	59
**T4**	12
**T5**	72
**T6**	25
**T7**	43
**T8**	39
**T9**	29
**T10**	63
**T11**	36
**T12**	29
**T13**	28
**T14**	57
**T15**	33

C

	% α-SMA+ / P-4E-BP1+
**T1**	21
**T2**	47
**T3**	53
**T4**	15
**T5**	74
**T6**	29
**T7**	37
**T8**	35
**T9**	22
**T10**	71
**T11**	37
**T12**	33
**T13**	23
**T14**	55
**T15**	34

D

	% α-SMA+ / IL-6+
**T1**	74
**T2**	79
**T3**	68
**T4**	89
**T5**	83
**T6**	62
**T7**	87
**T8**	81
**T9**	58
**T10**	92
**T11**	77
**T12**	74
**T13**	72
**T14**	81
**T15**	94

Duluc *et al*. Table S3

Protein	Mean		SEM		p value
	untreated	SOM230	untreated	SOM230	
ENA-78	2.05	0.66	1.18	0.32	0.09
GRO	12.89	1.55	5.72	1.00	0.03
GROα	9.44	0.51	5.47	0.27	0.04
IL3	3.92	2.29	1.80	1.54	0.19
IL6	29.13	0.39	6.13	0.10	0.00
IL7	3.61	0.32	1.09	0.12	0.01
IL8	9.94	2.08	2.58	1.54	0.01
IL10	3.66	2.29	1.24	1.67	0.20
MCP-1	17.62	0.77	15.16	0.40	0.10
MCP-3	5.97	0.56	1.99	0.20	0.01
MCSF	4.93	1.55	0.80	0.91	0.01
MIP-1β	3.95	2.82	1.82	2.16	0.30
Rantes	5.67	3.43	2.49	2.38	0.20
TNF-α	3.34	1.57	1.24	1.03	0.10
TNF-β	2.58	1.02	0.89	0.58	0.05
EGF	3.03	1.77	1.21	0.97	0.16
IGF-1	2.14	0.64	0.96	0.33	0.05
Angiogenin	8.24	0.43	7.12	0.18	0.10
Oncostatin M	6.57	2.14	1.61	1.31	0.02
Thrombopoeitin	2.96	0.76	1.24	0.42	0.04
VEGF	4.20	0.61	1.64	0.33	0.02
PDGF-BB	3.65	0.74	1.62	0.44	0.04
Leptin	3.49	0.69	1.67	0.35	0.04
BDNF	3.99	1.50	2.02	0.66	0.09
BLC	2.83	0.81	1.10	0.42	0.04
Ckβ 8-1	2.83	1.22	1.10	0.64	0.07
Eotaxin	2.43	0.95	0.94	0.54	0.06
Eotaxin-2	3.53	2.87	1.52	1.88	0.36
Eotaxin-3	2.10	0.86	1.03	0.43	0.10
FGF-4	3.31	1.19	1.48	0.72	0.07
FGF-6	2.66	0.75	1.17	0.39	0.05
FGF-7	2.85	1.03	1.11	0.59	0.05
FGF-9	3.24	1.20	1.31	0.65	0.06
Fit-3 ligand	2.91	0.58	1.39	0.26	0.04
fractalkine	3.03	0.64	1.32	0.22	0.03
GCP-2	4.96	0.78	0.51	0.34	0.00
GDNF	4.41	2.83	2.01	1.82	0.23
HGF	3.74	1.92	1.30	1.05	0.10
IGFBP-1	3.77	2.14	1.11	1.47	0.14
IGFBP-2	3.87	2.43	1.15	1.53	0.17
IGFBP-3	2.92	1.14	1.23	0.55	0.07
IGFBP-4	3.27	0.48	0.98	0.19	0.01
IL16	2.69	1.20	1.11	0.73	0.09
IP-10	2.67	1.24	1.10	0.65	0.09

LIF	3.86	0.55	2.18	0.23	0.05
LIGHT	3.04	1.13	1.52	0.68	0.09
MCP-4	2.53	0.45	0.79	0.18	0.01
MIF	3.27	1.49	1.29	0.86	0.09
MIP-3α	2.94	0.74	0.54	0.33	0.00
NAP-2	5.17	3.82	1.66	2.33	0.27
NT-3	2.85	1.39	1.08	0.54	0.08
NT-4	2.35	0.84	1.11	0.43	0.07
Osteopontin	2.34	0.86	0.90	0.39	0.05
Osteoprotegerin	3.04	0.73	1.02	0.43	0.02
PARC	2.24	0.98	1.07	0.56	0.11
PIGF	2.93	1.90	1.38	1.20	0.23
TGF-β2	4.33	3.88	2.25	2.04	0.42
TGF-β3	2.35	0.78	1.12	0.46	0.07
TIMP-1	8.06	2.57	6.00	1.87	0.14
TIMP-2	11.66	1.41	8.81	0.63	0.09

Duluc *et al.* Figure S1

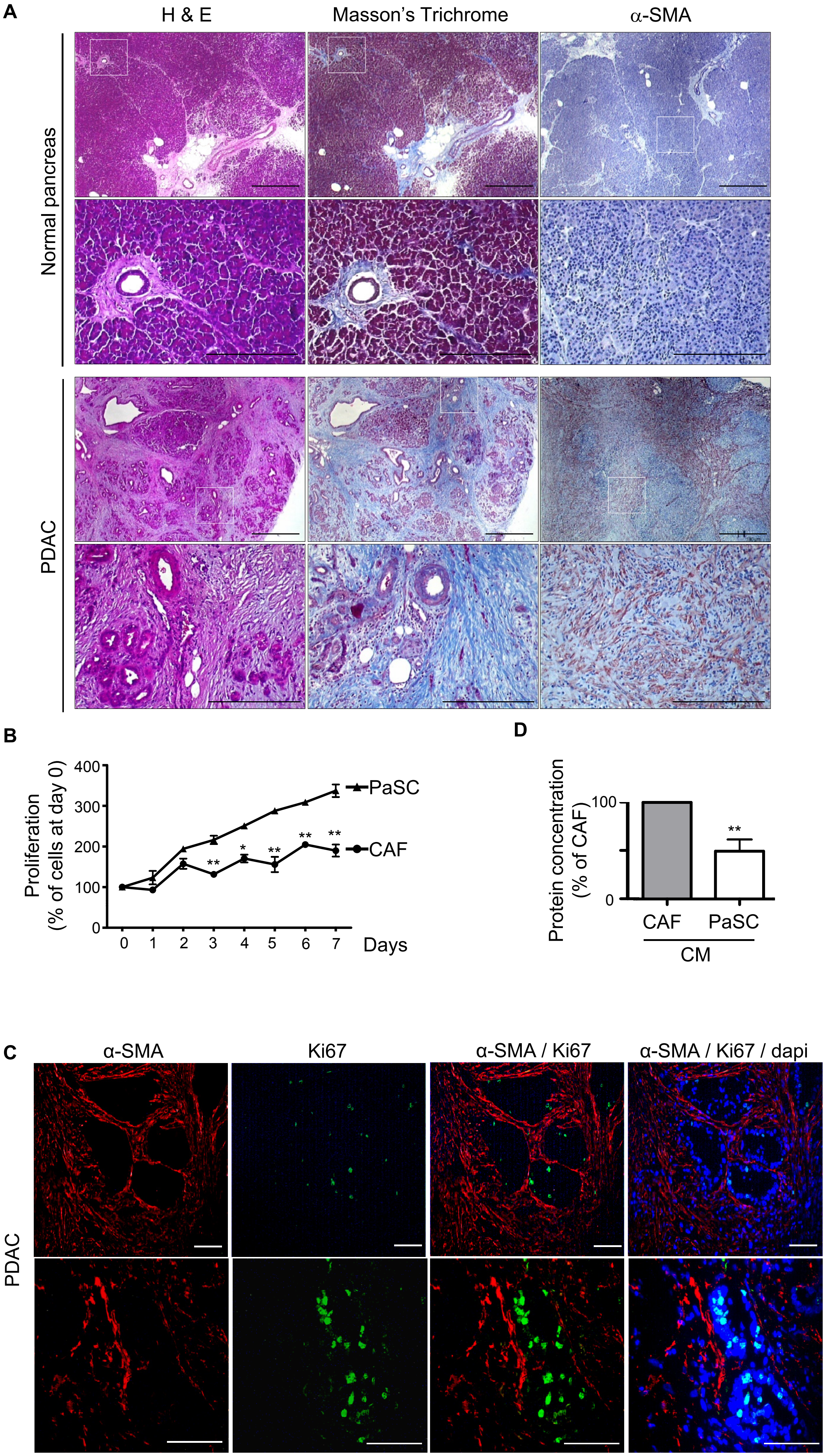

Duluc *et al.* Figure S2

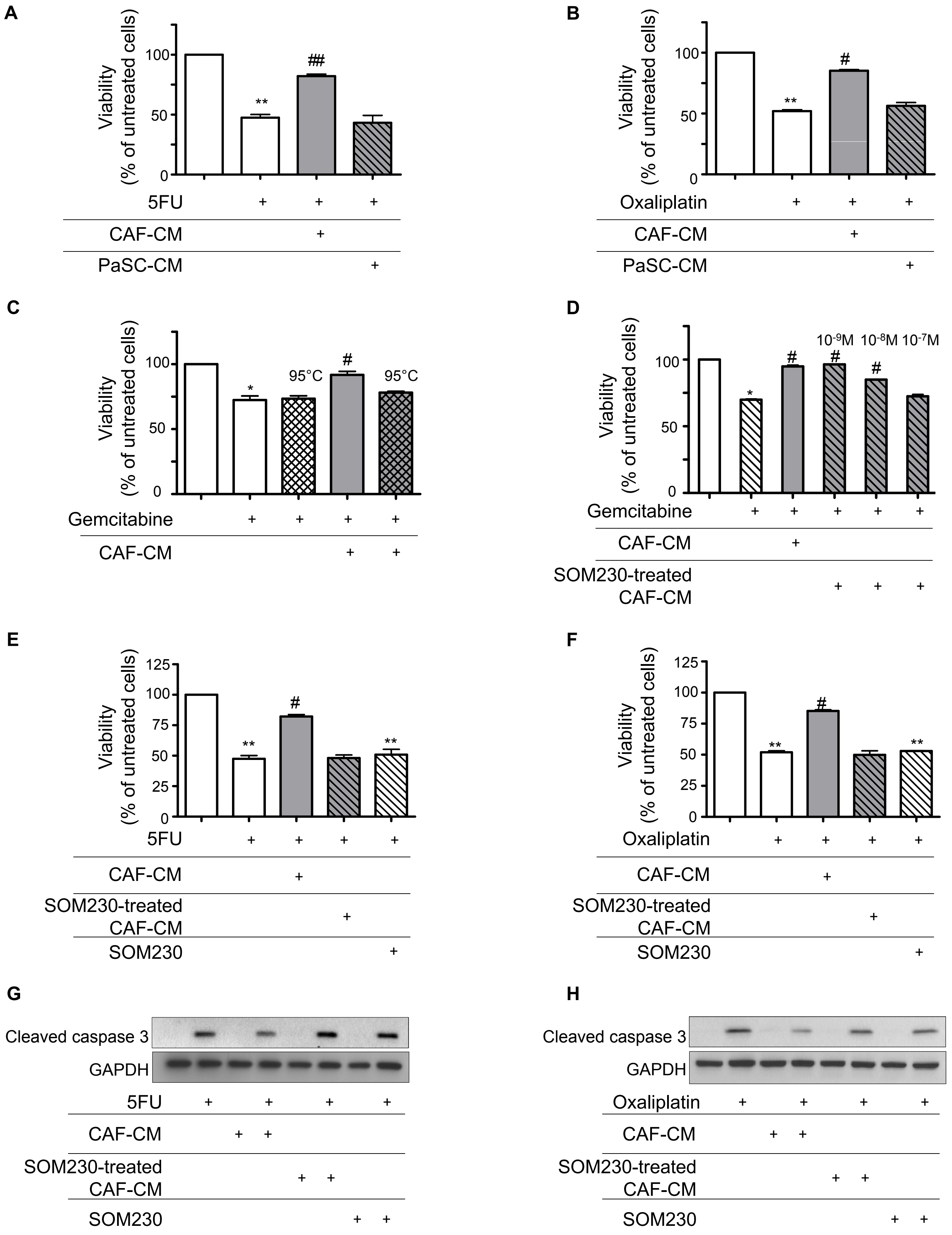

Duluc *et al.* Figure S3

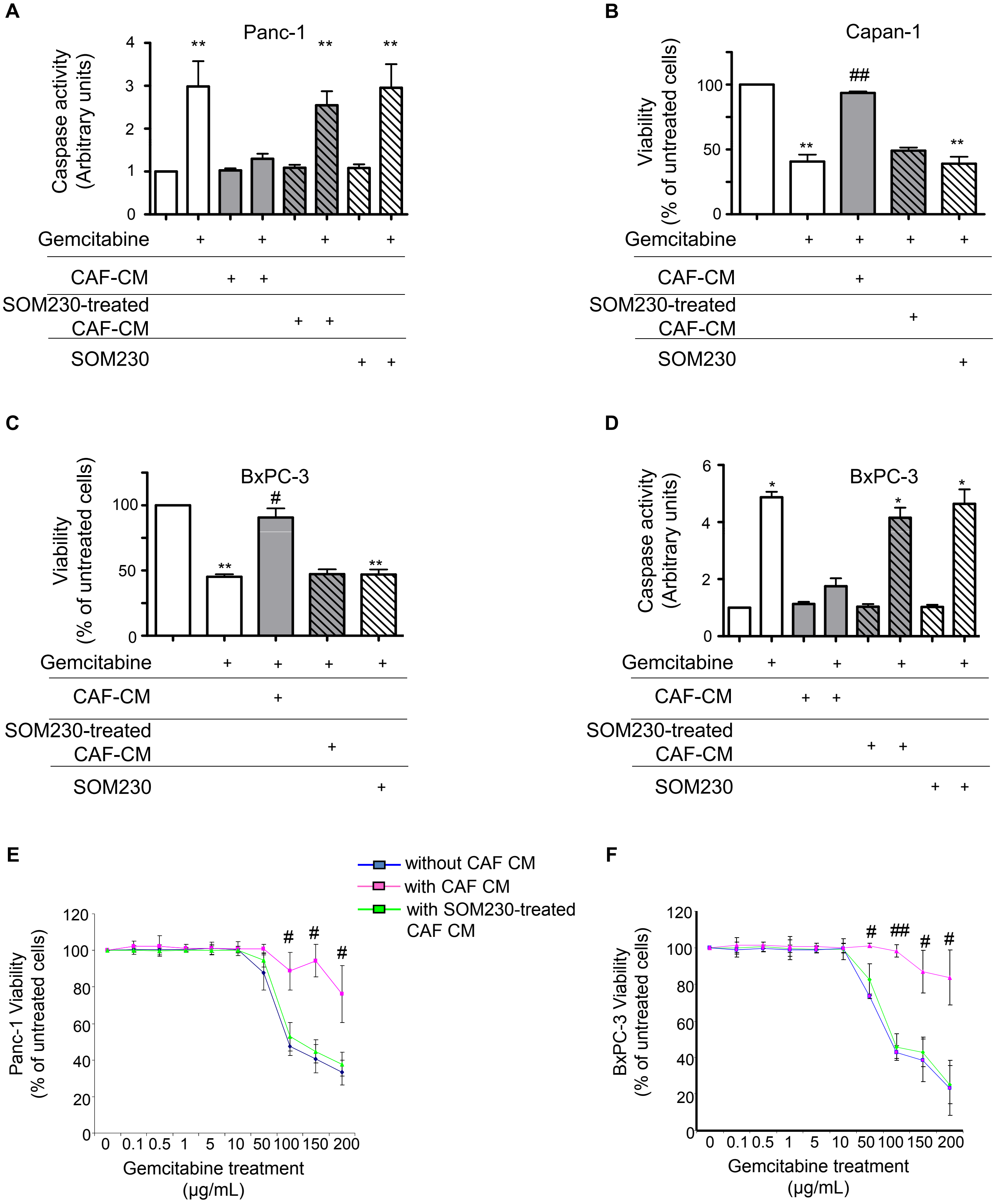

G

Cell lines	Panc-1	BxPC-3
IC50 Gemcitabine (µg/mL, 48h)	108 ± 6	83 ± 6
IC50 Gemcitabine (µg/mL, 48h) + CAF CM	> 200	> 200
IC50 Gemcitabine (µg/mL, 48h) + SOM230-treated CAF CM	111 ± 4	87 ± 7

Duluc *et al.* Figure S4

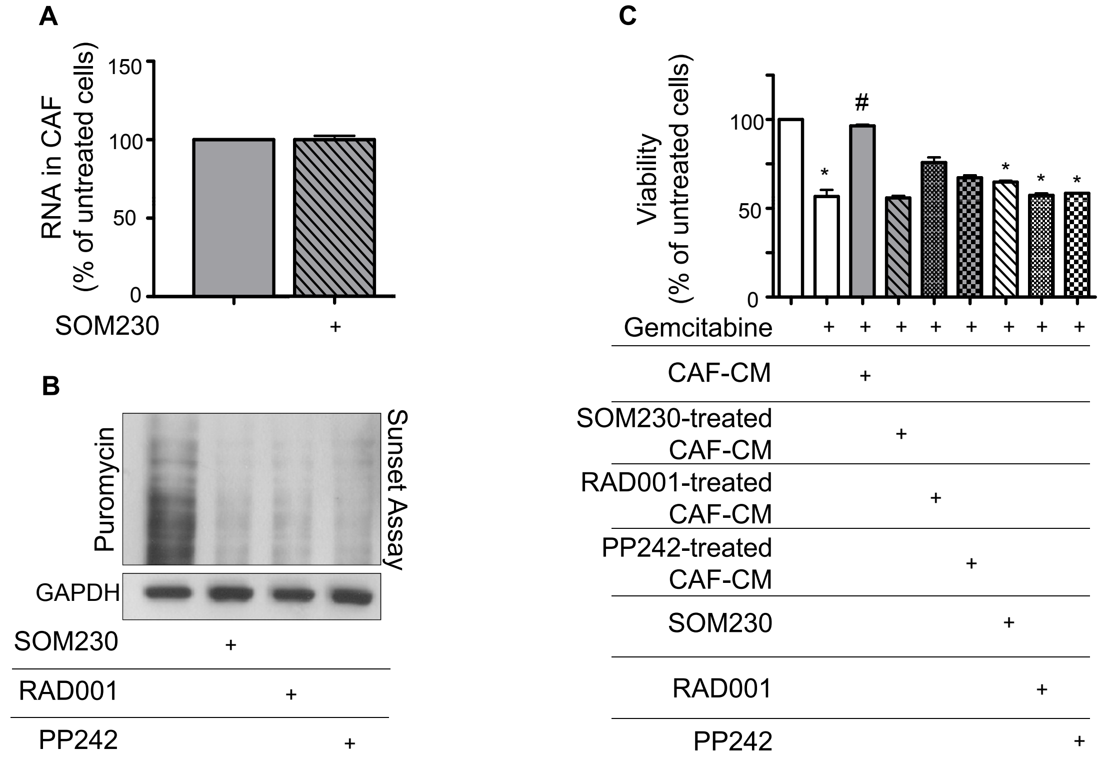

Duluc *et al.* Figure S5

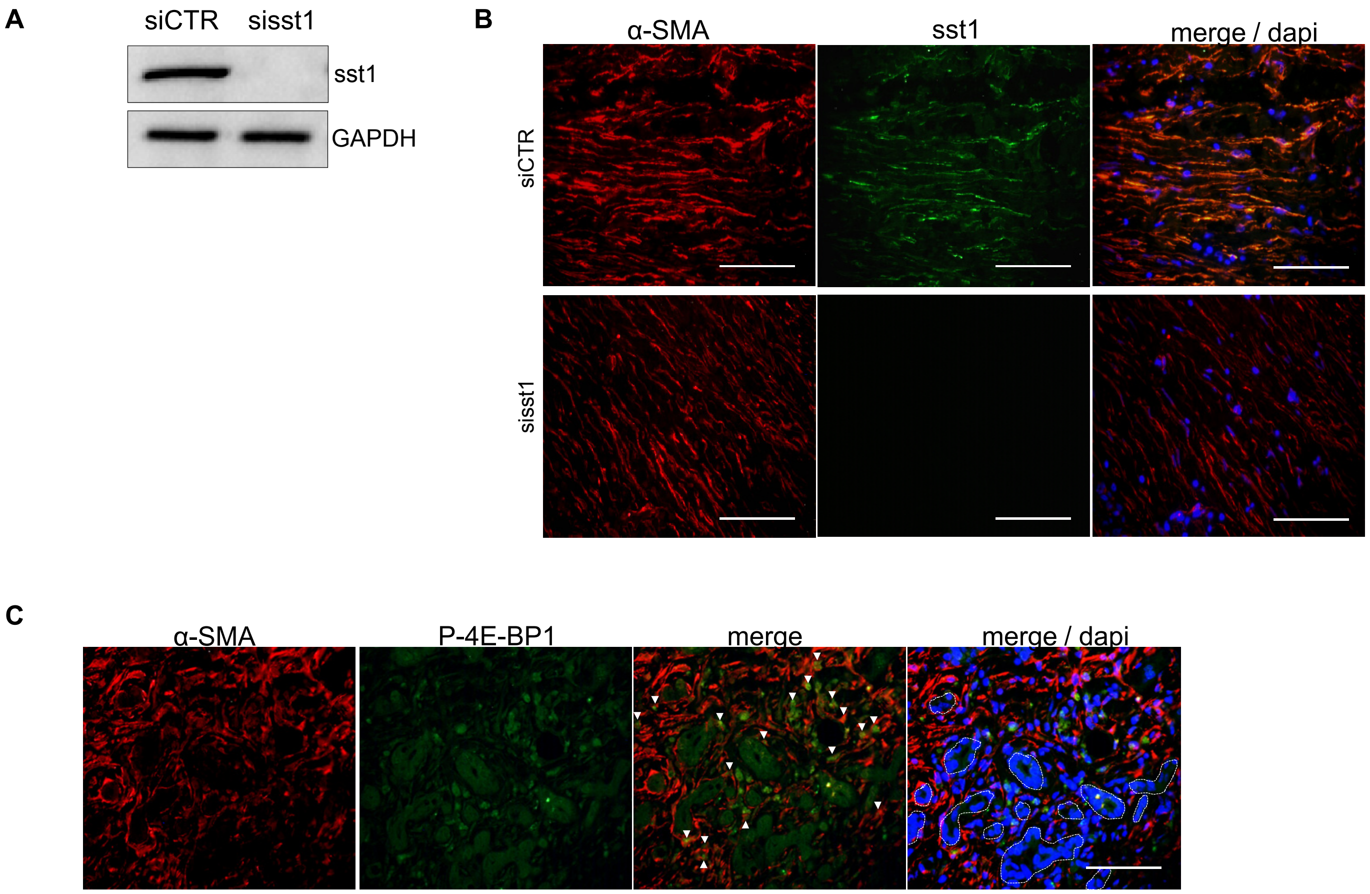

Duluc *et al.* Figure S6

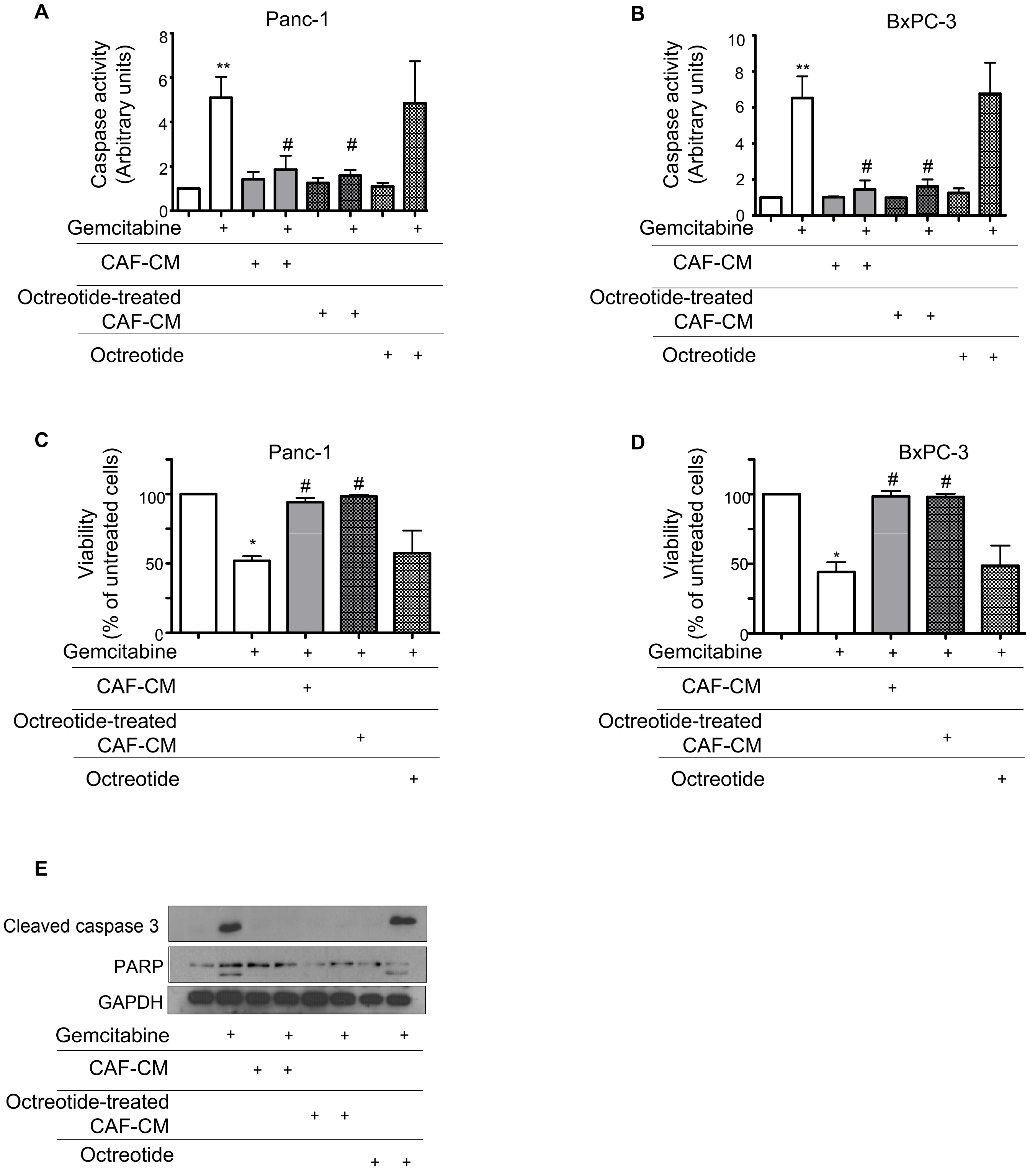

Duluc *et al.* Figure S7

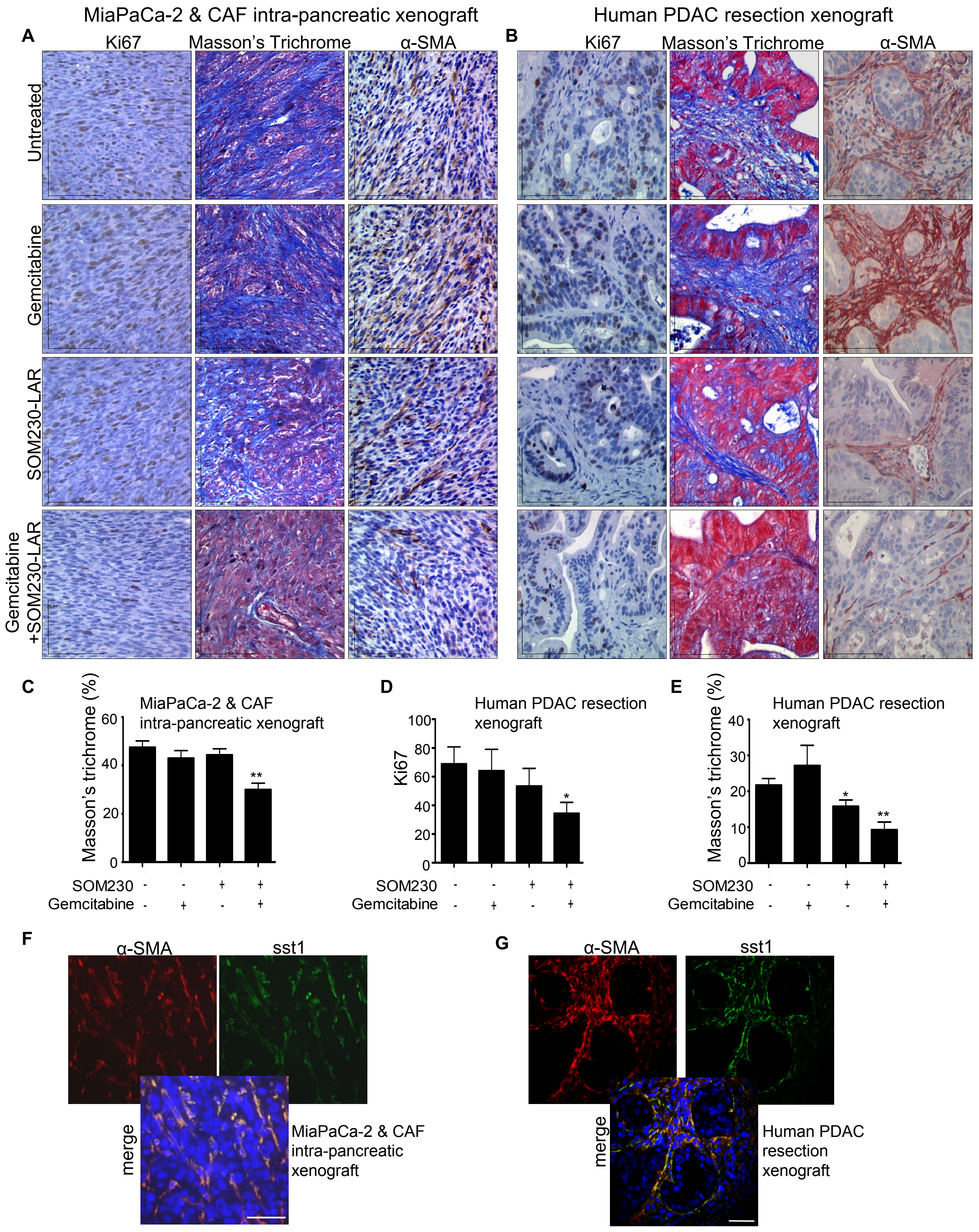

Duluc *et al.* Figure S8

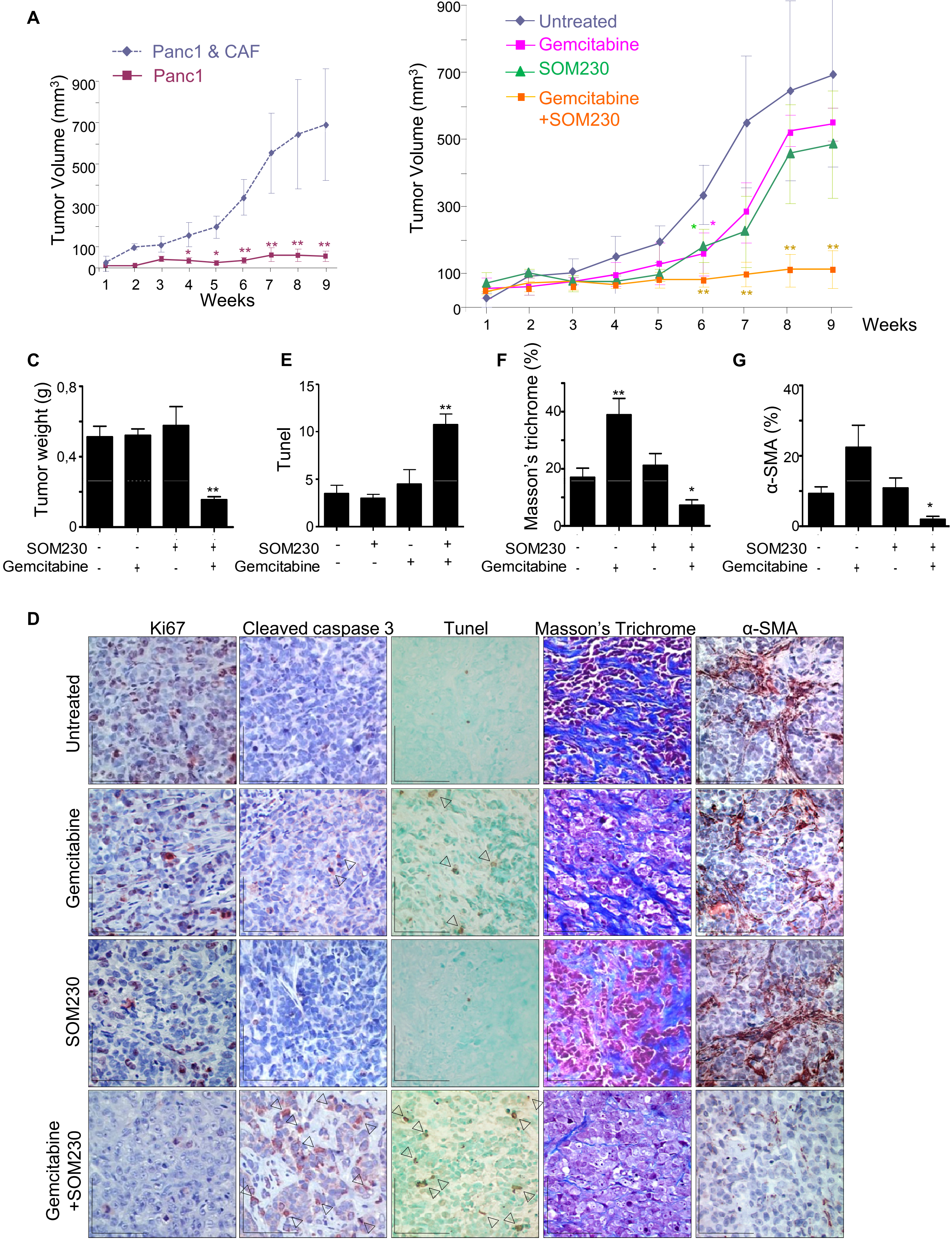

Duluc *et al.* Figure S9

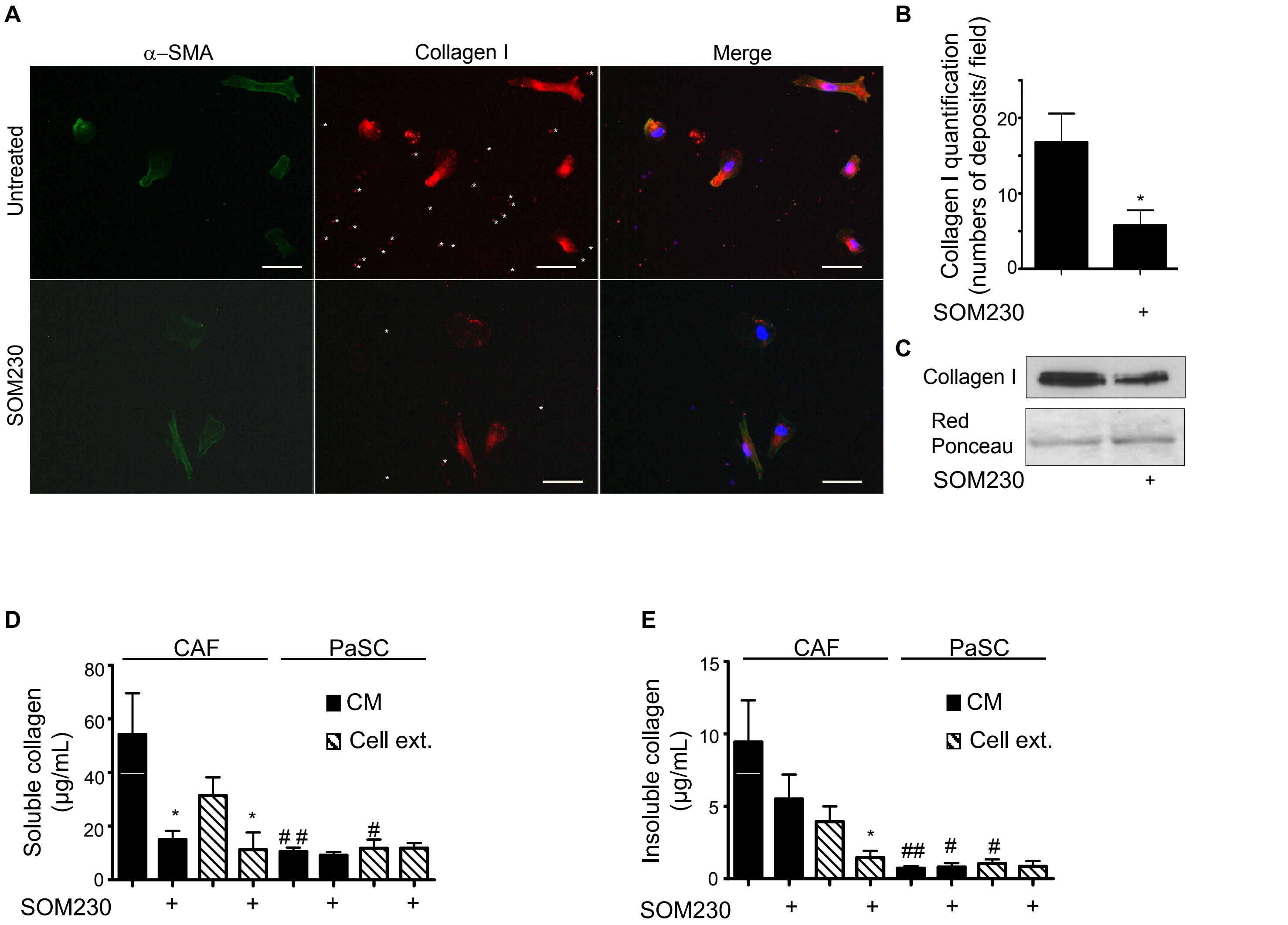

Duluc *et al.* Figure S10

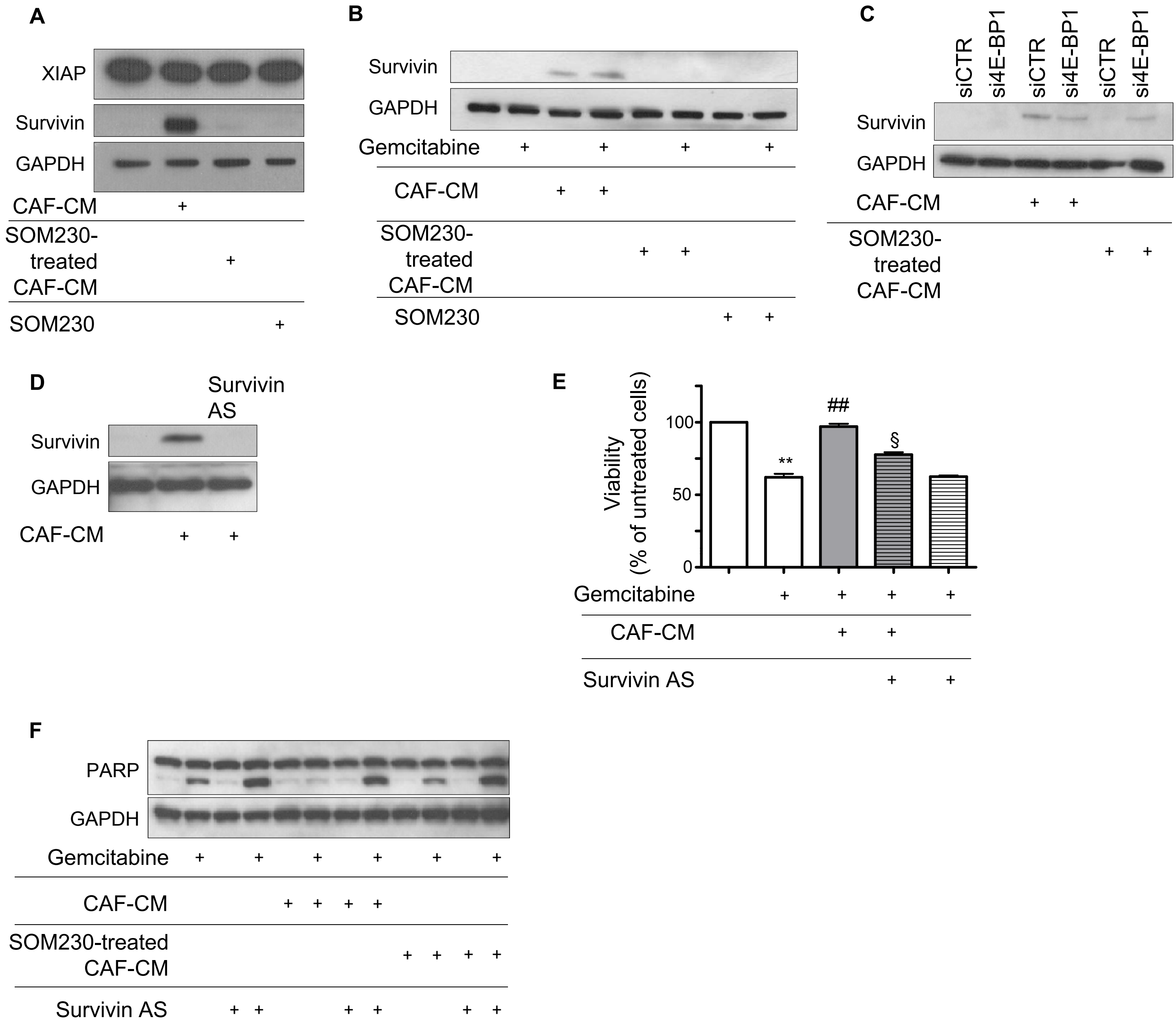

Duluc *et al.* Figure S11

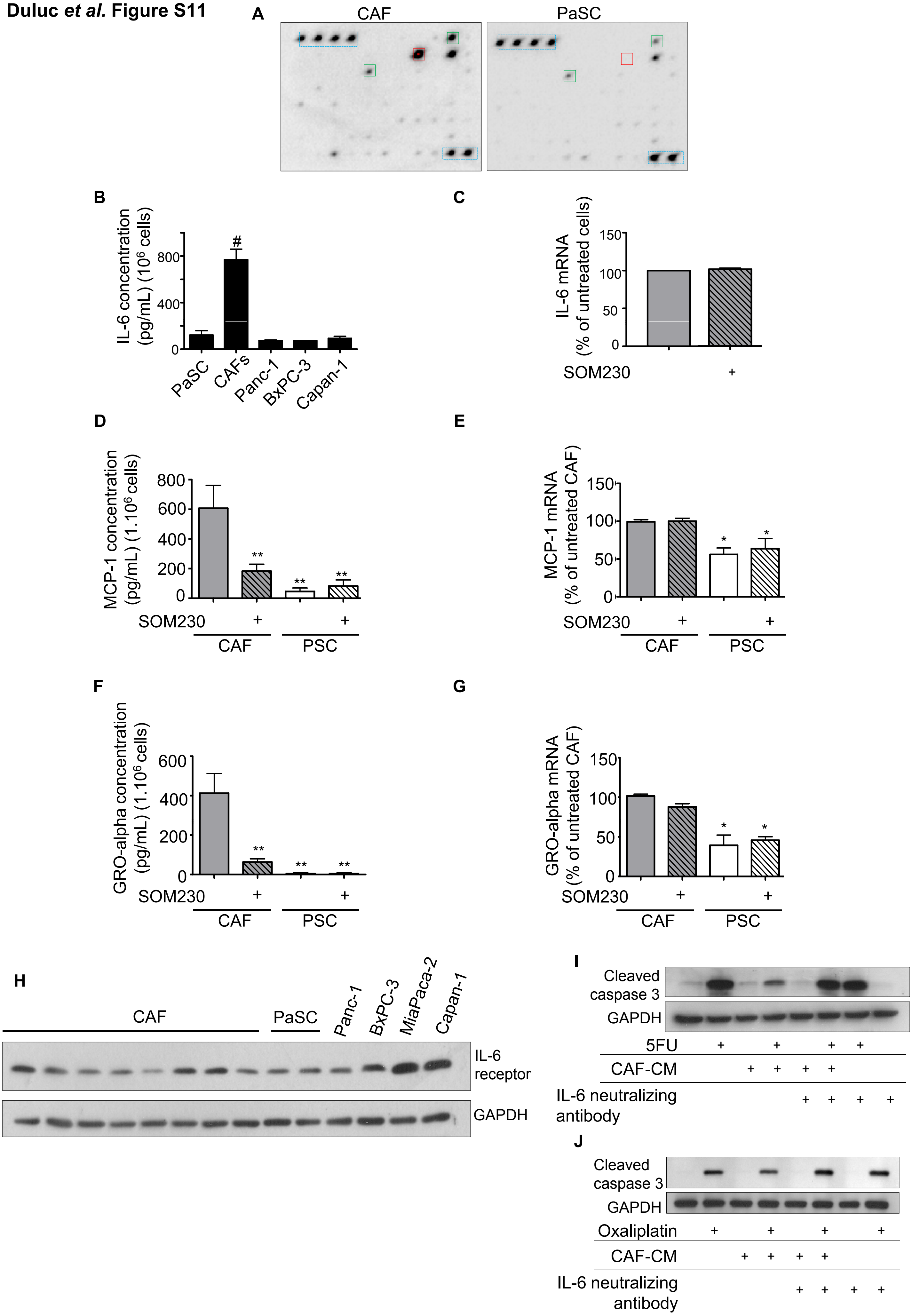

Duluc *et al.* Figure S12

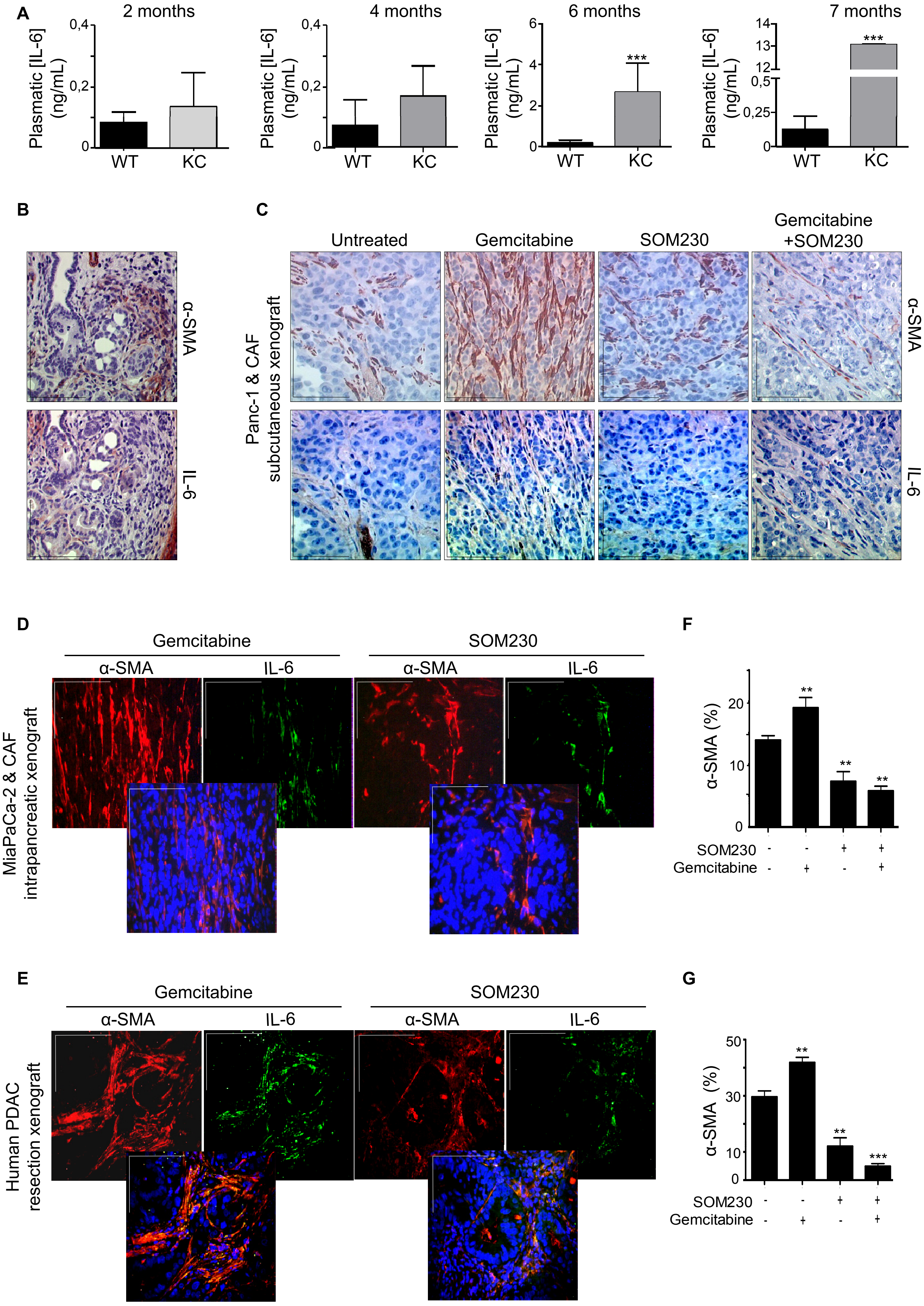

Supplement: Supplementary file 1 [file emmm0007-0735-sd1.pdf]
