# Supplementary material for: Pharmacological targeting of the protein synthesis mTOR/4E-BP1 pathway in cancer-associated fibroblasts abrogates pancreatic tumour chemoresistance
Source: EMBO Mol Med. 2015 Apr 1;7(6):735–53. doi: 10.15252/emmm.201404346 (PMC4459815; doi:10.15252/emmm.201404346)
Supplement: Supplementary file 2 [file emmm0007-0735-sd2.pdf]

EMBO Molecular Medicine

Pharmacological targeting of the protein synthesis mTOR/4E-BP1 pathway in cancer-associated fibroblasts abrogates pancreatic tumour chemoresistance

Camille Duluc, Siham Moatassim-Billah, Mounira Chalabi-Dchar, Aurélie Perraud, Rémi Samain, Florence Breibach, Marion Gayral, Pierre Cordelier, Marie-Bernadette Delisle, Marie-Pierre Bousquet-Dubouch, Richard Tomasini, Herbert Schmid, Muriel Mathonnet, Stéphane Pyronnet, Yvan Martineau and Corinne Bousquet

Corresponding author: Corinne Bousquet, INSERM U1037

Review timeline:

Submission date:	16 June 2014
Editorial Decision:	18 July 2014
Revision received:	26 November 2014F
Editorial Decision:	19 December 2014
Revision received:	20 February 2015
Accepted:	26 February 2015

Transaction Report:

(Note: With the exception of the correction of typographical or spelling errors that could be a source of ambiguity, letters and reports are not edited. The original formatting of letters and referee reports may not be reflected in this compilation.)

Editor: Roberto Buccione

1st Editorial Decision 18 July 2014

Thank you for the submission of your manuscript to EMBO Molecular Medicine. We have now heard back from the three Reviewers whom we asked to evaluate your manuscript.

You will see that the Reviewers are mostly supportive of you work, although all they do express a number of partly overlapping concerns that prevent us from considering publication at this time. I will not dwell into much detail, as the evaluations are detailed and self-explanatory. I would like, however, to highlight a few main points.

Reviewer 1 is of the opinion that the regulation of mTOR by somatostatin receptor activation requires further mechanistic analysis. S/he would also like to see, however, further experimentation in a GEMM.

Reviewer 2 is also positive but similarly to Reviewer 1, would like to improved mechanistic analysis. This Reviewer also requires clarification on a number of issues including specificity of the somatostatin analogue, further validation of the relevance of sst1 with additional means and other additional important items.

Reviewer 3 again similarly to Reviewer 1, would like to understand how somatostatin receptor activation regulates mTOR and suggests verifying whether CAF have activated a senescence programme. This Reviewer also lists other items for your action.

After Reviewer cross-commenting, I have agreed with the Reviewers that, although further experimentation on a pancreatic cancer GEMM would add value to the manuscript, this will not an absolute requirement, provided all other issues are thoroughly dealt with, including further experimentation where necessary.

Considered all the above, while publication of the paper cannot be considered at this stage, we would be pleased to consider a revised submission, with the understanding that the Reviewers' concerns must be addressed as outlined above, with additional experimental data where appropriate and that acceptance of the manuscript will entail a second round of review.

I look forward to seeing a revised form of your manuscript as soon as possible.

***** Reviewer's comments *****

Referee #1 (Comments on Novelty/Model System):

This is an interesting study but premature for publication. There are number of issues with the experimental in vivo model and the mechanisms mediating the regulation mTOR by sst1 activation that should be addressed to determine the impact of the study in the biology of pancreatic cancer.

Referee #1 (Remarks):

The manuscript by Dulue and colleagues provides evidence for a novel role of the somatostatin receptor 1 in the regulation of pancreatic cancer associated fibroblast function. Specifically, they demonstrate that activation of this receptor using a synthetic somatostatin analog decreases IL6 secretion by blocking mTOR/4EBP1 secretory pathway. The study is conceptually very interesting, and the data well presented. However, it lacks critical experimental data to support the authors' conclusion and biological value of these findings. First, the in vivo data has been done using subcutaneous model in immunodeficient animals, this model does not resemble the features of the human disease. Moreover, it has been shown that in the presence of an active immune system fibroblast plays a protective role. It is important that the authors repeat the in vivo experiments in a GEMM model of pancreatic cancer (e.g. KPC). Second, the mechanism regulation of mTOR is not clearly defined. The authors should define the downstream molecules mediating the inactivation of mTOR/4eBP1 pathway. Finally, the study will benefit from having an expanded analysis of the expression of the sst1 receptor in clinical specimens. Either by IHC or qPCR the authors should examine the levels of sst1, IL6 and mTOR activity in a sizeable cohort of patients.

Referee #2 (Comments on Novelty/Model System):

The model system used is appropriate and useful

Referee #2 (Remarks):

Review for: Pharmacological targeting of the protein synthesis mTOR/4E-BP1 pathway...
Duluc et al
Duluc et al present an interesting and intriguing set of studies that suggest cancer associated fibroblasts (CAFs) have a distinct secretory profile that directly promotes chemoresistance in pancreatic cancer. The contribution of CAFs to the tumor microenvironment is unquestioned and an area of intense study by many groups, this is especially relevant to pancreatic cancer, which is a desmoplastic disease. CAFs are central to the biology of pancreatic tumors where they have been associated with deposition of ECM, angiogenesis, metastasis, the cancer stem niche and immune evasion. Strategies to pharmacologically modify the activity of CAFs are of great interest. The present study suggests that CAFs express sst1, a receptor for somatostatin, and also the pharmacologic target for second generation somatostatin analog SOM230 (Pasireotide). SOM230 is used for the treatment of neuroendocrine tumors including PNET where it functions to inhibit secretion of autocrine and paracrine growth factors. The general thesis of the study is that inhibition of protein secretion from CAFs is a strategy to enhance the activity of chemotherapy for the treatment of pancreatic ductal adenocarcinoma.

General Strengths of the study
1) The observation that conditioned media from CAFs but not pancreatic stellate cells promotes chemoresistance.
2) Use of primary CAFs and pancreatic stellate cells.
3) Characterization of protein secretion by pancreatic stellate cells and CAFs.
4) Demonstration that SOM230 or siRNA of 4E-BP1 can abrogate the protective effect of CAF CM.
5) Use of multiple pancreatic cancer cell lines
6) Use of a PDX model.
Limitations that should be addressed:
1) SOM230 is not specific for sst1 in fact it is reported to have higher affinity for sst6, sst3, & sst2. What is the expression level of these sst receptors in the CAFs, pancreatic stellate cells and pancreatic tumor cells? Documentation at the level of RNA at least should be provided.
2) To further validate the function of sst1 in the phenotype of CAFs additional pharmacologic inhibitors could be employed however loss of function experiments using RNAi or other genetic methods should be explored.
3) The effect of CAF CM on the IC50 of gemcitabine should be shown; this is most cleanly shown by performing a gemcitabine dose response curve.
4) The requirement for CAFs for Panc1 tumor growth (e.g., Fig 4A, Fig S6) is odd. There are many publications that show robust Panc1 tumor growth without addition of CAFs. Further, isn't the tumor microenvironment enough to activate the CAF phenotype? Is there something unique about primary CAFs from human tumors?
5) The IHC shown in Figure 4E/G and Figure 7A/C are not convincing, these should be replaced with more robust results.
6) The surviving data in general is unconvincing. Are other IAPs similarly regulated?
7) In the context of the cytokines evaluated in Figure 6A, what is the cytokine spectrum of pancreatic stellate cells?
8) Collagen expression by CAFs is likely extremely relevant to tumor cell response to cellular stress. Fig. S7 attempts to quantify collagen by looking at immunoreactive collagen in vitro. This is not ideal. Collagen should be evaluated by evaluating detergent soluble and insoluble collagen which can be quantified more acutely.
9) The statements on page 4 in the first full paragraph that CAFs constitute a barrier to drug delivery and antifibrotic therapy by targeting CAFs offers new hope for PDAC therapy should be tempered and discussed in light of the fact that Hedgehog inhibition has failed in the clinic and further the two recent papers that suggest CAFs are also important in controlling the growth of PDAC (see Rhim et al Ca Cell 2014 and Ozdemir Ca Cell 2014).
10) The text should be edited for clarity.

Minor questions:
1) What is the expression of S100A4 in CAFs and pancreatic stellate cells?
2) Why use luciferase imaging for subcu tumors (Fig. 4A)?
3) The dose and schedule for gemcitabine and SOM230 in the in vivo experiments should be provided in the figure legends
4) Do pancreatic tumor cells express IL-6R?

Referee #3 (Remarks):

Title: Pharmacological targeting of the protein synthesis mTOR/4E-BP1 pathway in cancer-associated fibroblasts abrogates pancreatic tumor chemoresistance.
Authors: Duluc C et al

Summary: In the present report experiments are shown whereby pancreatic cancer associated fibroblasts (CAFs) promote the resistance of pancreatic carcinoma cells to treatment with the chemotherapy drug gemcitabine. This resistance was mediated via paracrine-acting factors (proteins) secreted by CAFs. Prominent among these therapy resistance-promoting factors was IL6. The somatostatin analog SOM230 was shown to suppress the pro-resistance influence of CAFs toward pancreatic cancer cells. This effect was mediated via ssr1 expressed on CAFs which acted

via mTOR/4E-BP1 to suppress the translation of IL6 (and potentially other secreted factors) and abrogate the influence of CAFs on tumor cell chemotherapy resistance.

Comments:

1. Overall, the manuscript is very well written. The introduction is clear, the figures are informative and relevant literature is cited. There are a few grammatical errors but these are quite minor.
2. There are several novel and noteworthy findings in the manuscript that include the finding that CAFs mediate pancreatic tumor cell resistance to chemotherapy, that pancreatic CAFs express the sst1 receptor; that the SOM230 analog suppresses CAF-mediated chemotherapy resistance; that SOM230 suppresses protein translation via mTOR/4E-BP1. The potential clinical relevance is high.
3. Though it is stated that SOM230 suppresses mTOR/4E-BP1-mediated translation, the exact mechanism by which it does so is not detailed. Some evidence by which the signaling from sst1 to 4E-BP1 activity would strengthen the conclusions.
4. While the data demonstrate that SOM230 modulates protein translation, a transcriptional mechanism controlling the expression of CAF genes is not ruled out. The investigators should show transcript levels (e.g. qRT-PCR or Northern or RNAseq) for several of the CAF genes compared with control cells (with and without SOM230) to confirm that a transcriptional mechanism (e.g. via another regulator such as NFkB - known to modulate IL6 expression) is not operative here.
5. The molecular program of the CAF secreted factors appears somewhat analogous to that observed with cellular senescence, the senescence associated secretory program (SASP). It would be useful to demonstrate that these cells are not senescence using Senescence-Associated Beta-Galactosidase assays, for example.
6. The expression data for sst1 in CAF cells is convincing, but the in vivo data is less convincing. It appears that 6 cases of human pancreatic cancer were evaluated. It would be useful to show in a quantitative fashion what percentage of cells in each case express sst1 and provide some data indicating the variation across cases. Also, six cases is marginal for such conclusions and it would be useful to have additional cases to support the clinical importance of this finding. Further, the a-SMA positive cells expressing sst1 appear to be a minority. It would be useful to discuss this subpopulation and provide some explanation as to the variability of sst1 expression

1st Revision - authors' response 26 November 2014

Our point-by-point response:

Reviewer 1:
This is an interesting study but premature for publication. There are number of issues with the experimental in vivo model and the mechanisms mediating the regulation mTOR by sst1 activation that should be addressed to determine the impact of the study in the biology of pancreatic cancer.

Comment 1:
The manuscript by Dulue and colleagues provides evidence for a novel role of the somatostatin receptor 1 in the regulation of pancreatic cancer associated fibroblast function. Specifically, they demonstrate that activation of this receptor using a synthetic somatostatin analog decreases IL6 secretion by blocking mTOR/4EBP1 secretory pathway. The study is conceptually very interesting, and the data well presented. However, it lacks critical experimental data to support the authors' conclusion and biological value of these findings.
First, the in vivo data has been done using subcutaneous model in immunodeficient animals, this model does not resemble the features of the human disease.

Our response:
In our manuscript, we use three different xenografting procedures in immunodeficient mice: 1/ orthotopic (intrapancreatic), or 2/ subcutaneous, co-xenografting of human pancreatic cancer cells and human primocultures of CAFs; and 3/ subcutaneous xenografting of human pancreatic tumor (PDX, Patient-Derived tumor Xenografting). These 3 models reproducibly demonstrate that combining the SOM230 treatment with the chemotherapeutic drug gemcitabine provides a

therapeutic benefit over each single treatment because SOM230, through its indirect action on CAFs, enables the re-sensitization of chemoresistant pancreatic cancer cells to gemcitabine's cytotoxicity. In these 3 models, our results demonstrate that the fibrotic stroma provided by the presence of CAFs and subsequent chemoprotection induced on pancreatic cancer cells mimics what occurs in human tumors, in contrast to xenografted models with pancreatic cancer cells alone (Pérez-Mancera PA, Gastroenterology 2012).

Comment 2:
Moreover, it has been shown that in the presence of an active immune system fibroblast plays a protective role. It is important that the authors repeat the in vivo experiments in a GEMM model of pancreatic cancer (e.g. KPC).

Our response:
We are aware that our results demonstrating the synergistic therapeutic interest of a chemotherapy (gemcitabine) and a pharmacotherapy (sst1 analog SOM230) will have to be, in a near future, validated on an immune competent model (such as the transgenic KPC model). Nevertheless our present manuscript aimed at demonstrating the novel proof of concept that protein synthesis is a promising therapeutic target in human CAFs. Our results show that protein synthesis targeting can be pharmacologically achieved using a somatostatin analog targeting the somatostatin receptor sst1 that is specifically expressed in human pancreatic CAFs, but not in normal pancreatic fibroblasts.

As mentioned by Reviewer 1, a recent paper has unexpectedly demonstrated in a mouse transgenic model developing a spontaneous PDAC (intra-pancreatic mutation of Kras and deletion of TGFR2) that depletion of α-SMA-positive and proliferating cells accelerates cancer progression, instead of stopping it, by inducing immunosuppression (Özdemir, Cancer Cell 2014 June 16). This study however presents the major drawback to target α -SMA-positive and proliferating cells which, in our opinion, 1/ is not specific to CAFs since other cell types are targeted by this strategy, including for example smooth muscle cells lining vessels whose function during tumor progression might be impacted in this model; 2/ concerns only proliferative CAFs (only proliferating cells are targeted in this system) which probably represents only a subpopulation of CAFs but perhaps not the sub-population that exhibits the highest secretory features. In our hands, in vitro primocultures of CAFs proliferate not as rapidly as normal pancreatic fibroblasts (doubling-time of 2-days vs. 6-days, respectively) (Fig. S1B) and IF using the Ki67 antibody on human PDAC tumors quasi-exclusively stain cancer cells, and not αSMA-positive cells (Fig. S1C). Whether there is a difference of proliferation between human and mouse CAFs present in human pancreatic tumors and in PDAC mouse models, respectively, will have to be explored.

Comment 3:
Second, the mechanism regulation of mTOR is not clearly defined. The authors should define the downstream molecules mediating the inactivation of mTOR/4eBP1 pathway.

Our response:
As recommended by Reviewers 1 & 3, we have explored molecular mechanisms underlying SOM230-mediated inhibition of mTOR/4E-BP1.
1/ Figs. 2C-D show that p-AKT, p-S6 and p-4E-BP1 are decreased (hypo-phosphorylation) upon SOM230 treatment;
2/ New Fig. 3I shows that in CAFs where sst1 expression is knock-down (using a siRNA targeting sst1), SOM230 is not able anymore to decrease p-AKT, p-S6 and p-4E-BP1, as well as protein synthesis (as measured in the SUnSET assay);
3/ sst1 is a GPCR coupled to Gαi; we have therefore pre-treated CAF with PTX (pertussis toxin) to inhibit $G_{\alpha}i$, and showed that it reverses SOM230 inhibitory effect on phosphorylation of AKT, S6, and 4E-BP1 (new Fig. 4A), indicating that SOM230 inhibitory effect on the PI3K/mTOR pathway is mediated through a $G_{\alpha}i$ protein. An inhibitor of $G_{\beta\gamma}$ did not have any effect on SOM230 action, indicating that, by opposition to GPCR activating PI3K, the ligand SOM230 / GPCR sst1 system does not inhibit PI3K through regulation of $G_{\beta\gamma}$ (new Fig. 4B).
4/ It has been previously demonstrated that sst1 induces the activation of phosphotyrosine phosphatases, including the SH2-containing SHP-2 and PTPε, this successive activation also involving the activity of Src (Arena S, Mol Endocrinol 2007). We have therefore investigated the

involvement of these effectors on SOM230 inhibitory effect on the PI3K/mTOR pathway in CAF (new Figs. 4C-F):

- CAF pre-treatment with the phosphatase inhibitor NSC87877 (NSC inhibits SHP-1, SHP-2 and PTPε) reverses SOM230 inhibitory effect on the PI3K/mTOR pathway activation (new Fig. 4C);
- CAF pre-treatment with the Src inhibitor (PP1) does not affect SOM230 inhibitory effect on the PI3K/mTOR pathway (new Fig. 4D);
- Extinction of SHP-2, but not of PTPε, expression in CAFs (transfected with a siRNA targeting SHP-2 or PTPε, respectively) also reverses SOM230 inhibitory effect on the PI3K/mTOR pathway activation (new Figs. 4E-F);

5/ We searched for upstream signals that may induce basal activation of the PI3K/mTOR pathway in CAFs.

- Because PDGF is a well-known signal that activates CAFs, CAFs were treated with a PDGFR inhibitor (AG1296) which efficiently inhibited basal activation of the PI3K/mTOR pathway, in contrast to the EGFR inhibitor (AG1478) (new Fig. 4G). CAF treatment with a JAK1/2 inhibitor (Ruxolitinib) also inhibited the activation of this pathway, consistent with our result using a IL-6 blocking antibody (Fig. 6I). These results indicate that the PI3K/mTOR pathway is activated in CAFs by the autocrine secretion of PDGF and JAK2-dependent cytokines including IL-6.
- Interestingly, addition of recombinant PDGF to CAF further enhances activation of this pathway (new Fig. 4H). SOM230 inhibits PDGF-induced activation of the PI3K/mTOR pathway by dephosphorylating PDGF-induced phosphorylation of Akt, S6 and 4E-BP1, which is reversed by the phosphotyrosine phosphatase inhibitor NSC87877. These results demonstrate that SOM230 inhibits basal and PDGF-induced activation of the PI3K/mTOR pathway in CAFs through activation of phosphotyrosine phosphatases including SHP-2.

Comment 4:
Finally, the study will benefit from having an expanded analysis of the expression of the sst1 receptor in clinical specimens. Either by IHC or qPCR the authors should examine the levels of sst1, IL6 and mTOR activity in a sizeable cohort of patients.

Our response:
As suggested by Reviewer 1, we have explored the expression of sst1, IL-6 and mTOR activity (by looking at p-4E-BP1) on additional human pancreatic tumors (n=15). To more specifically study in CAFs the expression or activation of those proteins, we have performed IF using both α-SMA and sst1 antibodies, or α-SMA and IL-6 antibodies, or α-SMA and p-4E-BP1 antibodies and confocal microscopy. We have quantified the % of α-SMA-positive cells that are also sst1-positive, or IL-6 positive, or p-4E-BP1-positive. The results are shown in new Figs. 3F-H and 7C. IF and confocal microscopy analyses for α-SMA and IL-6 have also been performed on tumors resulting from the orthotopic co- xenografting of CAFs+MiaPaCa-2 cells, and on Patient-Derived tumor Xenografts (PDX) where the co-expression in cells is shown (new Figs. 7A-B and S11D-E).

IF (confocal microscopy) on human pancreatic tumors reveals that α-SMA-positive cells that express sst1 represent 69% ± 27 of α-SMA-positive cells, highlighting an heterogeneity that will need to be further explored. In these tumors, α-SMA-positive cells that express phospho-4E-BP1 represent 39% ± 18 of α-SMA-positive cells, highlighting an activated status of the mTOR pathway in these cells *in vivo*. Finally, 78% ± 10 of α-SMA-positive cells of these human tumors also express IL-6 (Fig. 7C).

Reviewer 2:
The model system used is appropriate and useful
Duluc et al present an interesting and intriguing set of studies that suggest cancer associated fibroblasts (CAFs) have a distinct secretory profile that directly promotes chemoresistance in pancreatic cancer. The contribution of CAFs to the tumor microenvironment is unquestioned and an area of intense study by many groups, this is especially relevant to pancreatic cancer, which is a desmoplastic disease. CAFs are central to the biology of pancreatic tumors where they have been associated with deposition of ECM, angiogenesis, metastasis, the cancer stem niche and immune

evasion. Strategies to pharmacologically modify the activity of CAFs are of great interest. The present study suggests that CAFs express sst1, a receptor for somatostatin, and also the pharmacologic target for second generation somatostatin analog SOM230 (Pasireotide). SOM230 is used for the treatment of neuroendocrine tumors including PNET where it functions to inhibit secretion of autocrine and paracrine growth factors. The general thesis of the study is that inhibition of protein secretion from CAFs is a strategy to enhance the activity of chemotherapy for the treatment of pancreatic ductal adenocarcinoma.

General Strengths of the study
1) The observation that conditioned media from CAFs but not pancreatic stellate cells promotes chemoresistance.
2) Use of primary CAFs and pancreatic stellate cells.
3) Characterization of protein secretion by pancreatic stellate cells and CAFs.
4) Demonstration that SOM230 or siRNA of 4E-BP1 can abrogate the protective effect of CAF CM.
5) Use of multiple pancreatic cancer cell lines
6) Use of a PDX model.

Comment 1: SOM230 is not specific for sst1 in fact it is reported to have higher affinity for sst6, sst3, & sst2. What is the expression level of these sst receptors in the CAFs, pancreatic stellate cells and pancreatic tumor cells? Documentation at the level of RNA at least should be provided.

Our response:
We have observed by RT-qPCR that pancreatic cancer cells and PaSC don't express any ssts mRNA (sst1-sst5), whereas CAF express only sst1 mRNA (Fig. 3A and data not shown). As positive controls, we have used the human BON neuroendocrine cells (known to express ssts) {Lupp, 2013 #3521}{Xiao, 2012 #3523}. This result has been included in the text of our manuscript. Consistently, only CAF (n=7), but not PaSC (n=2) (Fig. 3B) or pancreatic cancer cells (n=5) (Fig. 3C), express the sst1 protein (Western-blot).

Comment 2: To further validate the function of sst1 in the phenotype of CAFs additional pharmacologic inhibitors could be employed however loss of function experiments using RNAi or other genetic methods should be explored.

Our response:
In Figs. 3I-J, we show that abrogating sst1 expression in CAF completely reverses SOM230 inhibitory effect on 1/ protein synthesis in CAF (Fig. 3I), 2/ chemoprotection provided by CAF conditioned media, restoring pancreatic cancer cell sensitivity to gemcitabine (Fig. 3J).
We have performed additional experiments investigating the effect on another somatostatin analog (octreotide) which has a nanomolar range affinity for sst2 and sst5, but does not activate sst1. As shown in new Fig. S5, CAF treatment with octreotide (10^{-8} M) is not able to reverse the chemoprotective features provided by their conditioned media on Panc-1 or BxPC-3 pancreatic cancer cells treated with gemcitabine, as measured using a caspase-3 activity assay (Figs. S5AB), survival MTT assay (Figs. S5C-D), or caspase-3 cleavage by Western-blot (Figs. S5E).
All together those results demonstrate that only the somatostatin sst1 receptor is expressed in CAFs and transduces SOM230 effects on these cells.

Comment 3:
The effect of CAF CM on the IC50 of gemcitabine should be shown; this is most cleanly shown by performing a gemcitabine dose response curve.

Our response:
We have performed dose-responses using gemcitabine (0-200 μg/mL) on Panc-1 and BxPC-3 cells, incubated or not with conditioned media from CAF treated or not with SOM230. Corresponding IC50 have been included in new Figs. S3E-G.

Comment 4:
The requirement for CAFs for Panc1 tumor growth (e.g., Fig 4A, Fig S6) is odd. There are many publications that show robust Panc1 tumor growth without addition of CAFs. Further, isn't the tumor microenvironment enough to activate the CAF phenotype? Is there something unique about primary CAFs from human tumors?

Our response:
Sub-cutaneous or orthotopic xenografting of Panc-1 or MiaPaCa-2 cells alone (without addition of CAF) has indeed been shown to enable tumor growth in many studies. However, to our experience, at least 5.10^6 - to - 10^7 pancreatic cancer cells need to be xenografted to have efficient tumor growth after 3-5 weeks. Our experiments have consisted in xenografting 10^6 pancreatic cancer cells only which is not sufficient to promote tumor growth unless CAFs ($3.10^{6)}$ are co-xenografted.
Xenografting human pancreatic cancer cells (alone) in nude mice indeed allows growth of tumors that are very cellular and less fibrotic than when CAFs are co-xenografted; some host-derived αSMA-positive cells are recruited but whether those mouse cells are sst1-positive in this model is currently under investigation. For our present manuscript, we have therefore preferred to perform our experiments similarly *in vitro* and *in vivo* using human-derived cells.

Comment 5: The IHC shown in Figure 4E/G and Figure 7A/C are not convincing, these should be replaced with more robust results.

Our response:
IHC to explore cleavage of caspase-3 have been replaced by IF in new Figs. 5E-H of our novel manuscript.
In new Figs. 7A-C, IHC have also been replaced by IF using both αSMA and IL-6 antibodies, to more specifically address in CAF the expression of those proteins. We have quantified the % of αSMA-positive cells that are also IL-6 positive (78% ± 3) in human pancreatic tumors (Fig. 7C).

Comment 6:
The surviving data in general is unconvincing. Are other IAPs similarly regulated?

Our response:
Those data have been placed in supplementary data (Fig. S9). Although the survivin expression (Western-blot) is not detected with a high intensity, survivin expression is reproducibly upregulated by CAF conditioned media (Figs. S9A-D), whereas CAF treatment with SOM230 abolished (in a 4E-BP1-dependent manner) this increase of survivin expression induced by their conditioned media (Figs. S9B-C). Furthermore, invalidation of survivin expression in pancreatic cancer cells reversed the chemoprotective effect induced by CAF conditioned media (partially on cell survival, Figs. S9D,E, but completely on PARP cleavage, Fig. S9D,F), demonstrating the critical role of survivin to mediate in pancreatic cancer cells chemoprotection induced by CAF conditioned media.
As suggested by Reviewer 2, the expression of other factors of the IAPs family has been investigated (XIAP, cIAP1, cIAP2, livin). As indicated in the novel version of our manuscript, "whereas the IAP family member XIAP, but not survivin or other IAPs (cIAP1, cIAP2, livin) (not detected), is highly expressed in pancreatic cancer cells, cell treatment with CAF-CM dramatically increased survivin, but not XIAP (or other IAPs, not detected), expression (Fig. S9A), suggesting a role for survivin (but not XIAP) in mediating CAF chemoprotective features." Accordingly, XIAP expression (Western-blot) has been added as new Fig. S9A.

Comment 7:
In the context of the cytokines evaluated in Figure 6A, what is the cytokine spectrum of pancreatic stellate cells?

Our response:
We have blotted a membrane antibody array with conditioned media from PaSC or CAFs, as shown in new Fig. S10. The expression of IL-6, Groα and MCP-1 are upregulated in CAF conditioned media as compared to PaSC, which has been confirmed by ELISA (Fig. 6B, new Fig. S10D,F). SOM230 dramatically decreases expression of IL-6, Groα and MCP-1 protein (ELISA), but did not have any effect at the mRNA expression (Figs. S10C, E-G), confirming a regulatory effect of this pharmacotherapy at the mRNA translation level.

Comment 8:
Collagen expression by CAFs is likely extremely relevant to tumor cell response to cellular stress.

Fig. S7 attempts to quantify collagen by looking at immunoreactive collagen in vitro. This is not ideal. Collagen should be evaluated by evaluating detergent soluble and insoluble collagen which can be quantified more acutely.

Our response:
We have now performed assays to quantify the soluble and unsoluble forms of collagen in conditioned media and cell extracts from PaSC and CAF, treated or not with SOM230 (new Fig. S8). Both forms of collagen present increased concentrations in CAFs as compared to PaSC, and in conditioned media as compared to cell extracts. Additionally, CAF treatment with SOM230 significantly decreased those concentrations of collagen (new Figs. S8D-E).

Comment 9:
The statements on page 4 in the first full paragraph that CAFs constitute a barrier to drug delivery and antifibrotic therapy by targeting CAFs offers new hope for PDAC therapy should be tempered and discussed in light of the fact that Hedgehog inhibition has failed in the clinic and further the two recent papers that suggest CAFs are also important in controlling the growth of PDAC (see Rhim et al Ca Cell 2014 and Ozdemir Ca Cell 2014).

Our response:
We have now tempered our discussion by taking into account those studies.
As discussed in the response addressed to Reviewer 1, a recent paper has indeed unexpectedly demonstrated in a transgenic PDAC model (intra-pancreatic mutation of Kras and invalidation of TGFR2) that depletion of α -SMA-positive and proliferating cells accelerates cancer progression, instead of stopping it, by inducing immunosuppression (Özdemir, Cancer Cell 2014 June 16). This study however presents the major drawback to target α-SMA-positive and proliferating cells which, in our opinion, 1/ is not specific to CAF since other cell types are targeted by this strategy, including for example smooth muscle cells lining vessels whose function during tumor progression might be impacted in this model; 2/ concerns only proliferative CAF (only proliferating cells are targeted in this system) which probably represents only a sub-population of CAF but perhaps not the sub-population that exhibits the highest secretory features. In our hands, in vitro primocultures of human CAF proliferate not as rapidly as normal pancreatic fibroblasts (doublingtime
of 2-days vs. 6-days, respectively) (Fig. S1B) and IF using the Ki67 antibody on human PDAC tumors quasi-exclusively stain cancer cells, and not αSMA-positive cells (Fig. S1C). Whether there is a difference of proliferation between human and mouse CAFs present in human pancreatic tumors and in PDAC mouse models, respectively, will have to be explored.

Comment 10:
The text should be edited for clarity.

Our response:
We have make efforts to edit the text for better clarity. If Reviewer 2 finds it necessary, we are willing to externalize our manuscript editing for english proofreading.

Minor questions:
1) What is the expression of S100A4 in CAFs and pancreatic stellate cells?

Our response: A Western blot for S100A4 (FSP-1) shows expression in PaSC, CAFs, as well as pancreatic cancer cells, indicating that this protein is not a good marker for CAF in pancreatic tumors since it is also expressed in the other cell components (Fig. below). Therefore, we have preferred not using it as a CAF marker for our study.

Figure :

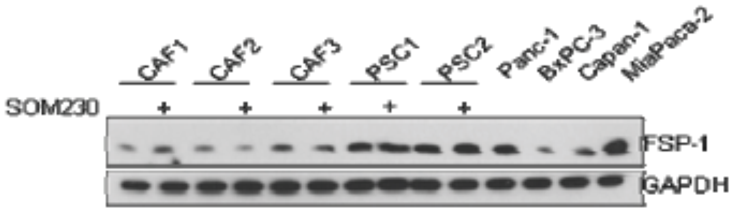

2) Why use luciferase imaging for subcu tumors (Fig. 4A)?

Our answer: We have used luciferase reporter just for intra-pancreatic tumors (new Fig. 5), but not for subcutaneous tumors (new Fig. S7). Indeed, secreted luciferase quantification is proportional to intrapancreatic tumor growth (new Fig. 5). Growth of sub-cutaneous xenografts has been measured using a caliper (new Fig. S7).

3) The dose and schedule for gemcitabine and SOM230 in the in vivo experiments should be provided in the figure legends

Our response: A detailed description of the procedure has now been added in the Figure legend.

4) Do pancreatic tumor cells express IL-6R?

Our response: new Fig. S10H shows that IL-6R is expressed in CAFs, PaSC and in pancreatic cancer
cells.

Reviewer 3:
Summary: In the present report experiments are shown whereby pancreatic cancer associated fibroblasts (CAFs) promote the resistance of pancreatic carcinoma cells to treatment with the chemotherapy drug gemcitabine. This resistance was mediated via paracrine-acting factors (proteins) secreted by CAFs. Prominent among these therapy resistance-promoting factors was IL6. The somatostatin analog SOM230 was shown to suppress the pro-resistance influence of CAFs toward pancreatic cancer cells. This effect was mediated via ssr1 expressed on CAFs which acted via mTOR/4E-BP1 to suppress the translation of IL6 (and potentially other secreted factors) and abrogate the influence of CAFs on tumor cell chemotherapy resistance.

Comments:
1. Overall, the manuscript is very well written. The introduction is clear, the figures are informative and relevant literature is cited. There are a few grammatical errors but these are quite minor.
2. There are several novel and noteworthy findings in the manuscript that include the finding that CAFs mediate pancreatic tumor cell resistance to chemotherapy, that pancreatic CAFs express the sst1 receptor; that the SOM230 analog suppresses CAF-mediated chemotherapy resistance; that SOM230 suppresses protein translation via mTOR/4E-BP1. The potential clinical relevance is high.
3. Though it is stated that SOM230 suppresses mTOR/4E-BP1-mediated translation, the exact mechanism by which it does so is not detailed. Some evidence by which the signaling from sst1 to 4E-BP1 activity would strengthen the conclusions.

Our response:
As answered to Reviewer 1, we have explored molecular mechanisms underlying SOM230-mediated inhibition of mTOR/4E-BP1.
1/ Figs. 2C-D show that p-AKT, p-S6K and p-4E-BP1 are decreased (hypo-phosphorylation) upon SOM230 treatment;
2/ New Fig. 3I shows that in CAFs where sst1 expression is knock-down (using a siRNA targeting

sst1), SOM230 is not able anymore to decrease p-AKT, p-S6 and p-4E-BP1, as well as protein synthesis (as measured in the SUnSET assay);
3/ sst1 is a GPCR coupled to Gαi; we have therefore pre-treated CAF with PTX (pertussis toxin) to inhibit $G_{\alpha}i$, and showed that it reverses SOM230 inhibitory effect on phosphorylation of AKT, S6, and 4E-BP1 (new Fig. 4A), indicating that SOM230 inhibitory effect on the PI3K/mTOR pathway is mediated through a $G_{\alpha}i$ protein; an inhibitor of $G_{\beta\gamma}$ did not have any effect on SOM230 action, indicating that, by opposition to GPCR activating PI3K, the ligand SOM230 / GPCR sst1 system does not inhibit PI3K through regulation of $G_{\beta\gamma}$ (new Fig. 4B).
4/ It has been previously demonstrated that sst1 induces the activation of phosphotyrosine phosphatases, including the SH2-containing SHP-2 and PTPε, this successive activation also involving the activity of Src (Arena S, Mol Endocrinol 2007). We have therefore investigated the involvement of these effectors on SOM230 inhibitory effect on the PI3K/mTOR pathway in CAF (new Figs. 4C-F):

- CAF pre-treatment with the phosphatase inhibitor NSC87877 (NSC inhibits SHP-1, SHP-2 and PTP ε) reverses SOM230 inhibitory effect on the PI3K/mTOR pathway activation (new Fig. 4C);
- CAF pre-treatment with the Src inhibitor (PP1) does not affect SOM230 inhibitory effect on the PI3K/mTOR pathway (new Fig. 4D);
- Extinction of SHP-2, but not of PTP ε, expression in CAFs (transfected with a siRNA targeting SHP-2 or PTP ε, respectively) also reverses SOM230 inhibitory effect on the PI3K/mTOR pathway activation (new Figs. 4E-F);

5/ We searched for upstream signals that may induce basal activation of the PI3K/mTOR pathway in CAFs.

- Because PDGF is a well-known signal that activates CAFs, CAFs were treated with a PDGFR inhibitor (AG1296) which efficiently inhibited basal activation of the PI3K/mTOR pathway, in contrast to the EGFR inhibitor (AG1478) (new Fig. 4G). CAF treatment with a JAK2 inhibitor (Ruxolitinib) also inhibited the activation of this pathway, consistent with our result using a IL-6 blocking antibody (Fig. 6I). These results indicate that the PI3K/mTOR pathway is activated in CAFs by the autocrine secretion of PDGF and JAK2-dependent cytokines including IL-6.
- Interestingly, addition of recombinant PDGF to CAF further enhances activation of this pathway (new Fig. 4H). SOM230 inhibits PDGF-induced activation of the PI3K/mTOR pathway by dephosphorylating PDGF-induced phosphorylation of Akt, S6 and 4E-BP1, which is reversed by the phosphotyrosine phosphatase inhibitor NSC87877. These results demonstrate that SOM230 inhibits basal and PDGF-induced activation of the PI3K/mTOR pathway in CAFs through activation of phosphotyrosine phosphatases including SHP-2.

4. While the data demonstrate that SOM230 modulates protein translation, a transcriptional mechanism controlling the expression of CAF genes is not ruled out. The investigators should show transcript levels (e.g. qRT-PCR or Northern or RNAseq) for several of the CAF genes compared with control cells (with and without SOM230) to confirm that a transcriptional mechanism (e.g. via another regulator such as NFkB - known to modulate IL6 expression) is not operative here.

Our response:
As answered to Reviewer 2, we have blotted a membrane antibody array with conditioned media from PaSC or CAFs, as shown on new Fig. S10. The expression of IL-6, Groα and MCP-1 are upregulated in CAF conditioned media as compared to PaSC, which has been confirmed by ELISA (Fig. 6B & new Figs. S10D,F). SOM230 dramatically decreases expression of IL-6, Groα and MCP-1 protein (ELISA), but did not have any effect at the mRNA expression (Figs. S10C, EG), confirming a regulatory effect of this pharmacotherapy at the mRNA translation level.

5. The molecular program of the CAF secreted factors appears somewhat analogous to that observed with cellular senescence, the senescence associated secretory program (SASP). It would be useful to demonstrate that these cells are not senescence using Senescence-Associated Beta-Galactosidase assays, for example.

Our response:
Senescence has been explored using the beta-galactosidase activity marker by IF (data not shown) and Western blot (see Fig. below). As shown, untreated or SOM230-treated CAF don't

express beta-galactosidase nor p21, whereas stressed H2O2-treated CAF express those senescence markers. These results indicate that the secretory features of pancreatic tumorderived CAFs are not senescence-related.

Figure :

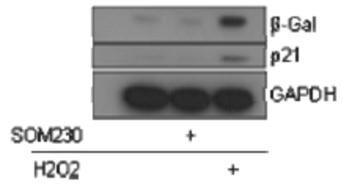

6. The expression data for sst1 in CAF cells is convincing, but the in vivo data is less convincing. It appears that 6 cases of human pancreatic cancer were evaluated. It would be useful to show in a quantitative fashion what percentage of cells in each case express sst1 and provide some data indicating the variation across cases. Also, six cases is marginal for such conclusions and it would be useful to have additional cases to support the clinical importance of this finding. Further, the a-SMA positive cells expressing sst1 appear to be a minority. It would be useful to discuss this subpopulation and provide some explanation as to the variability of sst1 expression

Our response:
As answered to Reviewer 1, we have explored the expression of sst1, IL-6 and mTOR activity (by looking at p-4E-BP1) on additional human pancreatic tumors (n=15). To more specifically study in CAFs the expression or activation of those proteins, we have performed IF using both α-SMA and sst1 antibodies, or α-SMA and IL-6 antibodies, or α-SMA and p-4E-BP1 antibodies and confocal microscopy. We have quantified the % of α-SMA-positive cells that are also sst1-positive, or IL-6 positive, or p-4E-BP1-positive. The results are shown in new Figs. 3F-H and 7C. IF and confocal microscopy analyses for α-SMA and IL-6 have also been performed on tumors resulting from the orthotopic co-xenografting of CAFs+MiaPaCa-2 cells, and on Patient-Derived tumor Xenografts (PDX) where the co-expression in cells is shown (new Figs. 7A-B and S11D-E).

IF (confocal microscopy) on human pancreatic tumors reveals that α-SMA-positive cells that express sst1 represent 69% ± 27 of α-SMA-positive cells, highlighting an heterogeneity that will need to be further explored. In these tumors, α-SMA-positive cells that express phospho-4E-BP1 represent 39% ± 18 of α-SMA-positive cells, highlighting an activated status of the mTOR pathway in these cells *in vivo*. Finally, 78% ± 10 of α-SMA-positive cells of these human tumors also express IL-6 (Fig. 7C).

2nd Editorial Decision 19 December 2014

Thank you for the submission of your revised manuscript to EMBO Molecular Medicine. We have now received the enclosed reports from the Reviewers that were asked to re-assess it. As you will see while Reviewers 1 and 2 are now globally supportive, Reviewer 3 remains critical.

Reviewer 1 would like you to include the discussion on the lack of testing on an immunocompetent model in the manuscript as well.

Reviewer 3 lists a number of serious concerns, some arising from your new experiments, and expresses numerous doubts on the conclusiveness of some of your claims.

After discussion with my colleagues, we have decided to invite a final revision, although we will not be asking you to provide further experimentation at this point, unless you should have further data that might strengthen your claims, in which case I would strongly suggest including them in your

manuscript. Apart from Reviewer 3's request to extend your experimentation to the GEMM, I would ask you to address and discuss each of the well-taken points s/he raises, with appropriate clarifications introduced in the main text.

Specifically, I would encourage you to 1) send me a rebuttal on the points raised by Reviewer 3 and 2) provide additional supporting data if available and/or amend your text as mentioned above and in general to better discuss the limitations of the study I am willing to make an Editorial decision on your final, revised version, provided the issues raised are dealt with as suggested.

Please also carefully consider the following final Editorial amendments/requests to be include in your revision:

1) As per our Author Guidelines, the description of all reported data that includes statistical testing must state the name of the statistical test used to generate error bars and P values, the number (n) of independent experiments underlying each data point (not replicate measures of one sample), and the actual P value for each test (not merely 'significant' or 'P < 0.05').

2) The manuscript must include a statement in the Materials and Methods identifying the institutional and/or licensing committee approving the experiments, including any relevant details (like how many animals were used, of which gender, at what age, which strains, if genetically modified, on which background, housing details, etc). We encourage authors to follow the ARRIVE guidelines for reporting studies involving animals. Please see the EQUATOR website for details: http://www.equator-network.org/reporting-guidelines/improving-bioscience-research-reporting-the-arrive-guidelines-for-reporting-animal-research/

3) We are now encouraging the publication of source data, particularly for electrophoretic gels and blots, with the aim of making primary data more accessible and transparent to the reader. Would you be willing to provide a PDF file per figure that contains the original, uncropped and unprocessed scans of all or at least the key gels used in the manuscript? The PDF files should be labeled with the appropriate figure/panel number, and should have molecular weight markers; further annotation may be useful but is not essential. The PDF files will be published online with the article as supplementary "Source Data" files. If you have any questions regarding this just contact me.

4) Every published paper now includes a 'Synopsis' to further enhance discoverability. Synopses are displayed on the journal webpage and are freely accessible to all readers. They include a short standfirst (to be written by the editor) as well as 2-5 one sentence bullet points that summarise the paper (to be written by the author). Please provide the short list of bullet points that summarise the key NEW findings. The bullet points should be designed to be complementary to the abstract - i.e. not repeat the same text. We encourage inclusion of key acronyms and quantitative information. Please use the passive voice. Please attach these in a separate file or send them by email, we will incorporate them accordingly.

5) "The Paper "Explained". All EMBO Molecular Medicine articles are accompanied by a summary to emphasize the major findings in the paper and their medical implications for the non-specialist reader. Please provide a draft summary of your article highlighting the medical issue you are addressing, the results obtained and their clinical impact. This may be edited to ensure that readers understand the significance and context of the research. Please refer to any of our published articles for an example.

6) I warmly encourage you to improve the style of your narrative. I find the writing a bit verbose and with too many modifiers; also, the verbs and prepositions are not always in concurrence. This would make reading easier and would ultimately benefit your work. Some examples: "Elevated mRNA translation into secreted proteins in..." could be simply "The secretome of.." and "totally overcome" could be simply "reduced" etc. Also, there are too many emphatic adverbs and adjectives, e.g. "extremely", "highly".

7) Please upload separate manuscript (word doc) and figure files (one per figure).

I look forward to seeing a revised form of your manuscript.

***** Reviewer's comments *****

Referee #1 (Comments on Novelty/Model System):

The authors have been very responsive to the reviewers' critiques and the manuscript has sufficiently improved since the initial submission. Thus, I recommend it for publication in the present format.

Referee #1 (Remarks):

The manuscript has improved, the new data has helped support the main conclusions of the study. However, it is a pity that the authors did not test the agent in a immunocompetent animal model. The discussion should include comments on this following the same line to what the authors wrote in their response. Also, the manuscript should included a cartoon illustrating the model supported by the data.

Referee #2 (Comments on Novelty/Model System):

The model systems include primary CAFs isolated from human
PDA, PDX and cell line xenograft tumors, orthotopic and subcu. The inclusion of a GEMM or syngenic model would dramatically strengthen the story but is not required in my opinion.

Referee #2 (Remarks):

Review for: Pharmacological targeting of the protein synthesis mTOR/4E-BP1 pathway...
Duluc et al Revised submission.
My impression of this study after reviewing the original submission was that it was an innovative study with potential for significant impact. The revised version has been strengthened in many areas. In particular they have provided evidence that CAFs express Sst1 but do not express other ssts that have affinity for SOM230 (comment 1). Further, new Figs 3I and J provide strong evidence that sst1 is required for the effect of SOM230 (comment 2). New immunofluorescence shown in new Figs 5E-H and 7A-C are significant improvements over the prior images (comment 5) and support the thesis of the study. The addition of XIAP expression analysis to Fig. S9 is useful and highlights the potential function of survivin in tumor cells after exposure to CAF-CM (comment 6). The addition of the membrane antibody array in Fig. S10 is effective (comment 7). The quantification of collagen expression in CAFs after treatment with SOM230 is useful (comment 8), although Fig S8A is not very effective as the deposits of collagen marked by asterisks are difficult to see (this could be removed from the final version, not a critical issue). They have also discussed relevant recent literature (comment 9). I thank the authors for also addressing my minor concerns effectively. Overall the revised version is much improved and the study has strong potential to provide impact on the field.

Referee #3 (Comments on Novelty/Model System):

There are no ethical issues. The genetically engineered model system of pancreatic cancer is not exploited to a sufficient extent to support the conclusions from the xenograft studies.

Referee #3 (Remarks):

Title: Pharmacological targeting of the protein synthesis mTOR/4E-BP1 pathway in cancer-associated fibroblasts abrogates pancreatic tumor chemoresistance.

Authors: Duluc C et al

Summary: This is a revised manuscript that describes a mechanism by which pancreatic cancer associated fibroblasts (CAFs) promote the resistance of pancreatic carcinoma cells to treatment with the chemotherapeutic agents. Resistance is shown to be mediated via paracrine-acting factors secreted by CAFs, specifically IL6. The somatostatin analog SOM230 was shown to suppress the pro-resistance influence of CAFs toward pancreatic cancer cells which is mediated via ssr1 expressed on CAFs which acts via mTOR/4E-BP1 to suppress the translation of IL6 and abrogate the influence of CAFs on tumor cell chemotherapy resistance.

Comments:

1. The investigators have provided detailed responses to the queries from the initial review. These are thoughtful and supported by new data.

2. However, there remain a number of moderate weaknesses which continue to reduce the impact of the overall results as several experiments are partial in terms of the molecular and clinical responses and the study would be further strengthened by addressing them. These are as follows:

a. The authors claim that the sst1 agonist SOM230 is acting exclusively via CAFs as pancreatic cancer cells do not express sst1. However, no data are shown from direct human pancreatic cancers demonstrating sst1 expression in cancer-associated stroma and lack of expression in tumor cells. Further, this idea is not supported by recent literature showing that a highly specific antibody to sst1 shows robust sst1 expression in 100% of pancreatic adenocarcinomas (see XX). This results calls into question the pancreatic carcinoma cell lines that are used in these studies that do not express sst1, if indeed sst1 is expressed consistently in human pancreatic cancer in vivo.

b. The in vivo IHC/IF studies of serial human PDAC are very informative where 69% of SMA-positive stromal cells also express sst1 and 39% of SMA-positive cells are positive for 4E-BP1. However, the key data showing that the SMA-positive, sst1 positive cells are those with 4EBP1 expression is not provided, nor is IL6 expression shown to occur in those cells with sst1 expression.

c. Data are shown indicating that PDGF and PDGFR represent key signaling factors contributing to baseline PI3K/mTOR activation. However, PDGF nor PDGFR staining is not shown for the in vivo PDAC samples, again leaving this line of inquiry unresolved.
Also not shown is PDGFR expression in CAFs in vitro and PDGF production/secretion by CAFs at baseline compared to PaSCs. It is stated that PDGF is a 'well known signal that activates CAFs'-but no reference supporting this statement in pancreatic cancer (or other cancer) is provided.

d. It is unclear why SOM230 treatment did not diminish the xenografted tumor growth since they hypothesize that this diminishes the 'basal' PI3K/mTOR activity of autocrine PDGF-driven CAF secretory activity, one would expect tumor growth to be substantially diminished by SOM230 treatment alone (Fig 5A). It would be useful to understand the investigators' hypothesis for this result.

e. The genetically-engineered mouse model (Pdx1-Cre; KrasG12D/+,) of pancreatic cancer is very underutilized in the studies and confirming that the findings observed in the more artificial xenograft systems also occur in the GEM model would greatly strengthen the study. The IL6 results support the conclusions. However, not evaluated are many of the other observations. For example, demonstrating sst1 expression is present in CAFs in vivo would be informative as would showing that these CAFs also express PDGFR and have active mTOR, etc. Also, it is not clear why the SOM230 and gemcitabine studies were not conducted in this model?

Minor Points:
a. 'Cancerogenesis' is not a commonly used word. It would be useful to have the manuscript carefully edited for common grammar/syntax.

b. 15 cases of PDAC remains a small number with which to draw strong conclusions regarding expression (tumor/stroma) of various biomolecules.

c. Methods for the GEM work do not appear to be included.

2nd Revision - authors' response 20 February 2015

Comments to the Editor:

- To introduce the limitations of our study (not performed on an immune mouse model), we have added the paragraph below in our discussion:
"Three different xenografting procedures, i.e. orthotopic (intrapancreatic) or subcutaneous co-xenografting of human pancreatic cancer cells and human CAF primary cell cultures, and subcutaneous xenografting of human pancreatic tumours (PDX, Patient-Derived tumour Xenografting) were undertaken in immunodeficient mice. These three strategies reproducibly demonstrated that combining the SOM230 treatment with the chemotherapeutic drug gemcitabine provided a therapeutic benefit over each single treatment. Through its indirect action on CAFs, SOM230 enabled the re-sensitization of chemoresistant pancreatic cancer cells to gemcitabine cytotoxicity. In these three models, our results demonstrate that the fibrotic stroma, and subsequent pancreatic cancer cell chemoprotection, provided by the presence of CAFs, mimicked that which normally occurs in human tumours, in contrast to xenografted models which only have pancreatic cancer cells (Perez-Mancera et al, 2012). However the therapeutic benefit provided by our drug association will have to be validated on immune competent model(s) of PDAC, especially as CAFs also impact on immune cell function (Mace et al, 2013). Nonetheless, our results strongly support protein synthesis as a novel therapeutic target in human CAFs."

- Because Referee 3 has raised some concerns regarding the putative expression of sst1 in pancreatic cancer cells (looking at human pancreatic tumor samples), we have edited / added the following Figures:

o Figs. S5A-B address the specificity of the signal provided by the anti-sst1 antibody used in our study, both in Western-blot and immunofluorescence experiments, using protein extracts (A) or formalinfixed paraffin-embedded samples (B), from CAFs transfected with a non-targeting siRNA (siCTR) or a siRNA targeting sst1 expression (sisst1);

o novel Fig. 3E: co-stain of sst1 with cytokeratin-19 (expressed only in epithelial (cancer) cells) shows that sst1 does not co-localize with cytokeratin-19 (n=42 human PDAC samples), precluding sst1 expression in pancreatic cancer cells;

o novel Figs. 3E-G (pictures chosen on serial slides of human PDACs), Tables S2A-B: co-stains of sst1 with either cytokeratin-19 (E), or with α-SMA (F), or with P-4E-BP1 (G), show that:

- sst1 is not expressed in epithelial cancer cells (see point above), but is expressed
in 69% ± 19 of α-SMA-positive cells (quantified in n=42 PDACs, T1-T42); in PDAC stroma, all sst1-positive cells expressed a-SMA (Fig. 3F);
- a significant proportion of sst1-positive (40% ± 16) cells (quantified in n=15 PDACs, T1-T15) also yielded positive staining for the phosphorylated (inhibited) form of 4E-BP1, indicating PI3K/mTOR pathway activation in sst1-expressing cells (Figs. 3G, Table S2B);

o Tables S2B-C: co-stains of sst1 with P-4E-BP1 (40% ± 16, quantified in n=15 PDACs, T1-T15), and of α-SMA with P-4E-BP1 (39% ± 17, quantified in n=15 PDACs, T1-T15), are correlated (r=0.96), further indicating PI3K/mTOR pathway activation in sst1-expressing α-SMA-positive cells;

o Because the quantifications using the anti-sst1 antibody have been performed by confocal immunofluorescence microscopy (co-stains sst1/α-SMA, sst1/P-4E-BP1), and the specificity of the anti-sst1 antibody validated by immunofluorescence, we suggest to remove Fig. 3E (previous
manuscript version, illustrating sst1 expression by immunohistochemistry).

- Grammar/synthax (Abstract, Synopsis, Introduction, Results, Discussion) has been edited by a native englishspeaker.
- Your Editorial Assistance has asked us to improve contrast / resolution of some of our gels. Accordingly we have inserted novel exposure for Figs. 1F, 2C, 2I, 3B, 6E. We have also inserted a novel blot (cleaved caspase-3) in support of PARP cleavage experiment in Fig. 2I.

Referee's point-by-point response:

Referee 1
Referee #1 (Comments on Novelty/Model System):
The authors have been very responsive to the reviewers' critiques and the manuscript has sufficiently improved since the initial submission. Thus, I recommend it for publication in the present format.

Referee #1 (Remarks):
The manuscript has improved, the new data has helped support the main conclusions of the study. However, it is a pity that the authors did not test the agent in a immunocompetent animal model. The discussion should include comments on this following the same line to what the authors wrote in their response. Also, the manuscript should included a cartoon illustrating the model supported by the data.

- This paragraph has been added in the first page of our discussion:
"Three different xenografting procedures, i.e. orthotopic (intrapancreatic) or subcutaneous co-xenografting ofhuman pancreatic cancer cells and human CAF primary cell cultures, and subcutaneous xenografting of human pancreatic tumours (PDX, Patient-Derived tumour Xenografting) were undertaken in immunodeficient mice. These three strategies reproducibly demonstrated that combining the SOM230 treatment with the chemotherapeutic drug gemcitabine provided a therapeutic benefit over each single treatment. Through its indirect action on CAFs, SOM230 enabled the re-sensitization of chemoresistant pancreatic cancer cells to gemcitabine cytotoxicity. In these three models, our results demonstrate that the fibrotic stroma, and subsequent pancreatic cancer cell chemoprotection, provided by the presence of CAFs, mimicked that which normally occurs in human tumours, in contrast to xenografted models which only have pancreatic cancer cells (Perez-Mancera et al, 2012). However the therapeutic benefit provided by our drug association will have to be validated on immune competent model(s) of PDAC, especially as CAFs also impact on immune cell function (Mace et al, 2013). Nonetheless, our results strongly support protein synthesis as a novel therapeutic target in human CAFs."

- A cartoon is presented in Fig. 8 of the novel version of our manuscript (previously placed as Fig. S12).

Referee 3
Referee #3 (Comments on Novelty/Model System):
There are no ethical issues. The genetically engineered model system of pancreatic cancer is not exploited to a sufficient extent to support the conclusions from the xenograft studies.

Referee #3 (Remarks):
Title: Pharmacological targeting of the protein synthesis mTOR/4E-BP1 pathway in cancer-associated fibroblasts abrogates pancreatic tumor chemoresistance.

Authors: Duluc C et al

Summary: This is a revised manuscript that describes a mechanism by which pancreatic cancer associated fibroblasts (CAFs) promote the resistance of pancreatic carcinoma cells to treatment with the chemotherapeutic agents. Resistance is shown to be mediated via paracrine-acting factors secreted by CAFs, specifically IL6. The somatostatin analog SOM230 was shown to suppress the pro-resistance influence of CAFs toward pancreatic cancer cells which is mediated via ssr1 expressed on CAFs which acts via mTOR/4EBP1 to suppress the translation of IL6 and abrogate the influence of CAFs on tumor cell chemotherapy resistance.

Comments:

1. The investigators have provided detailed responses to the queries from the initial review. These are thoughtful and supported by new data.

2. However, there remain a number of moderate weaknesses which continue to reduce the impact of the overall results as several experiments are partial in terms of the molecular and clinical responses and the study would be further strengthened by addressing them. These are as follows:

a. The authors claim that the sst1 agonist SOM230 is acting exclusively via CAFs as pancreatic cancer cells do not express sst1. However, no data are shown from direct human pancreatic cancers demonstrating sst1 expression in cancer-associated stroma and lack of expression in tumor cells. Further, this idea is not supported by recent literature showing that a highly specific antibody to sst1 shows robust sst1 expression in 100% of pancreatic adenocarcinomas (see XX). This results calls into question the pancreatic carcinoma cell lines that are used in these studies that do not express sst1, if indeed sst1 is expressed consistently in human pancreatic cancer in vivo.

b. The in vivo IHC/IF studies of serial human PDAC are very informative where 69% of SMA-positive stromal cells also express sst1 and 39% of SMA-positive cells are positive for 4E-BP1. However, the key data showing that the SMA-positive, sst1 positive cells are those with 4EBP1 expression is not provided, nor is IL6 expression shown to occur in those cells with sst1 expression.

c. Data are shown indicating that PDGF and PDGFR represent key signaling factors contributing to baseline PI3K/mTOR activation. However, PDGF nor PDGFR staining is not shown for the in vivo PDAC samples, again leaving this line of inquiry unresolved.
Also not shown is PDGFR expression in CAFs in vitro and PDGF production/secretion by CAFs at baseline compared to PaSCs. It is stated that PDGF is a 'well known signal that activates CAFs'-but no reference supporting this statement in pancreatic cancer (or other cancer) is provided.

d. It is unclear why SOM230 treatment did not diminish the xenografted tumor growth since they hypothesize that this diminishes the 'basal' PI3K/mTOR activity of autocrine PDGF-driven CAF secretory activity, one would expect tumor growth to be substantially diminished by SOM230 treatment alone (Fig 5A). It would be useful to understand the investigators' hypothesis for this result.

e. The genetically-engineered mouse model (Pdx1-Cre; KrasG12D/+,) of pancreatic cancer is very underutilized in the studies and confirming that the findings observed in the more artificial xenograft systems also occur in the GEM model would greatly strengthen the study. The IL6 results support the conclusions. However, not evaluated are many of the other observations. For example, demonstrating sst1 expression is present in CAFs in vivo would be informative as would showing that these CAFs also express PDGFR and have active mTOR, etc. Also, it is not clear why the SOM230 and gemcitabine studies were not conducted in this model?

Minor Points:
a. 'Cancerogenesis' is not a commonly used word. It would be useful to have the manuscript carefully edited for common grammar/syntax.
b. 15 cases of PDAC remains a small number with which to draw strong conclusions regarding expression (tumor/stroma) of various biomolecules.
c. Methods for the GEM work do not appear to be included.

Points 2a-b.
Immunofluorescence confocal microscopy analyses using the sst1 antibody show a sst1 staining only in α-SMApositive cells, but not in human pancreatic cancer cells, as previously shown in previous Fig. 3F (human PDAC samples where the white dashed line delineates the tumor gland) and S6G (PDX xenografted model).

3F:

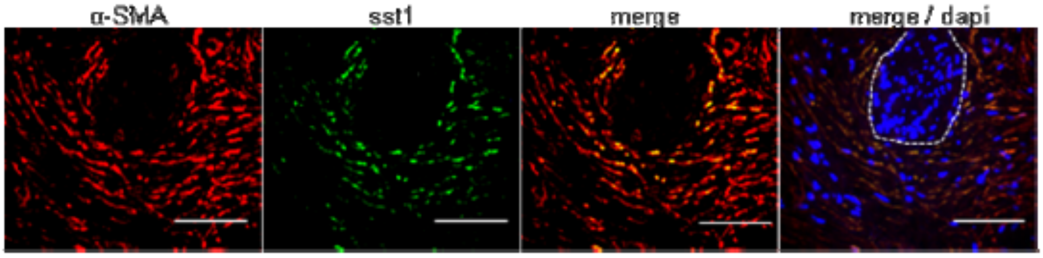

S6G:

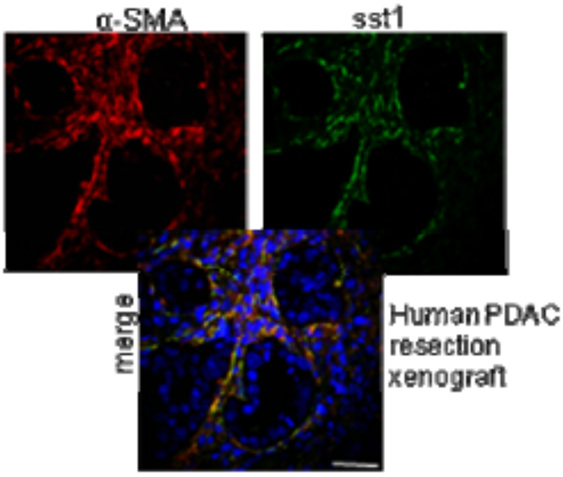

To further strengthen these data, we have:

- added additional data (novel Figs. S5A-B) where we validate the specificity of our anti-sst1 antibody by Western-blot and immunofluorescence using protein extracts (A) or formalin-fixed paraffin-embedded samples (B), from CAFs transfected with a non-targeting siRNA (siCTR) or a siRNA targeting sst1 expression (sisst1);

- added additional immunofluorescence experiments:

o novel Fig. 3E: co-stain of sst1 with cytokeratin-19 (expressed only in epithelial (cancer) cells) shows that sst1 does not co-localize with cytokeratin-19 (n=42 human PDAC samples), precluding sst1 expression in pancreatic cancer cells;

o novel Figs. 3E-G (pictures chosen on serial slides of human PDACs), Tables S2A-B: co-stains of sst1 with either cytokeratin-19 (E), or with α-SMA (F), or with P-4E-BP1 (G), show that:

- sst1 is not expressed in epithelial cancer cells (see point above), but is expressed
  in 69% ± 19 of α-SMA-positive cells (quantified in n=42 PDACs, T1-T42); in PDAC stroma, all sst1-positive cells expressed a-SMA (Fig. 3F);
- a significant proportion of sst1-positive (40% ± 16) cells (quantified in n=15 PDACs, T1-T15) also yielded positive staining for the phosphorylated (inhibited) form of 4E-BP1, indicating PI3K/mTOR pathway activation in sst1-expressing cells (Figs. 3G, Table S2B);

© EMBO

o Tables S2B-C: co-stains of sst1 with P-4E-BP1 (40% ± 16, quantified in n=15 PDACs, T1-T15), and of α-SMA with P-4E-BP1 (39% ± 17, quantified in n=15 PDACs, T1-T15), are correlated (r=0.96), further indicating PI3K/mTOR pathway activation in sst1-expressing α-SMA-positive cells;

Point 2c.

The originality of our paper is to have unraveled elevated intrinsic protein synthesis rates in CAFs through high activation of the PI3K/mTOR pathway whose pharmacological targeting can be achieved using the somatostatin SOM230 analogue and provides potent chemosensitization in pancreatic cancer. Mechanisms underlying SOM230 inhibitory effect on intrinsic elevated mRNA translation in CAFs have been dissected and involve the phosphotyrosine phosphatase SHP-2, as demonstrated using pharmacological and RNA interference strategies (Figs. 4A-F).

The aim of this manuscript was not to unravel which signal(s) are responsible for the high intrinsic activation of the PI3K/mTOR pathway in CAFs. Nevertheless, our data indeed show that inhibitors of PDGFR and of Jak1/2 (Fig. 4G), as well as the IL-6 neutralizing antibody (Fig. 6I), decrease the activation of the PI3K/mTOR pathway in CAFs, revealing potential mediators of such intrinsic pathway activation, which will have to be further dissected.

PDGF is a well-known activator of PaSCs and PDGFR are reportedly expressed in these cells (Bachem MG, Gastroenterology 2005), where PDGF stimulates cell migration at least through activation of PI3K (McCarroll JA, Biochem Pharmacol 2004). Our results indeed show that PDGF activates in CAFs PI3K (Akt phosphorylation) but also the mTOR targets (S6 and 4E-BP1 phosphorylation) (Fig. 4I). Our intention was here to show that SOM230 is also able to decrease PDGF-activated PI3K/mTOR pathway, involving a phosphotyrosine phosphatase activity (Fig. 4I). Our aim was not here to demonstrate that PDGF is the main regulator of the observed elevated intrinsic PI3K/mTOR activity in CAFs. We aim to address Referee 3's experiment requests on PDGF/PDGFR expression in CAFs and in human PDACs in a future manuscript.

Point 2d.
The following paragraph below has been included in the discussion:
"In orthotopic co-xenograft cancer cell+CAF and PDX (Patient-Derived tumour sub-cutaneous Xenograft) experiments (Figs. 5 and S7), SOM230 treatment alone diminished CAF activation but was not able to affect cancer cell proliferation or apoptosis. In PDX experiments, SOM230 significantly decreased tumour progression, probably due to a reduction in the rate of collagen deposition by CAFs (Masson's trichrome staining). No expression of sst1 was observed in any of the five pancreatic cancer cell lines (Panc-1, BxPC-3, Capan-1, MiaPaCa-2, CFPAC-1), or in pancreatic cancer cells from forty-two human PDACs, as assessed by Western-blot or immunofluorescence analyses, respectively, using an anti-sst1 antibody, the specificity of which had been validated here. Therefore, our results show that the inhibitory effect of SOM230 on CAF secretions is insufficient to affect tumour growth, but is sufficient to provide potent chemosensitization, bypassing pancreatic cancer cell resistance to gemcitabine. Our hypothesis for these differences is that pancreatic cancer cells may be able to adapt in vivo to the decrease in CAF-derived growth signals induced by SOM230 treatment, but not to the absence of CAF-derived chemoprotective factors. Therefore, these results emphasize the role of CAFs as critical chemoprotective cell partners to pancreatic cancer cells, and further strengthen the idea that, to kill cancer cells, a chemotherapy must be combined with a targeted therapy (such as the SOM230 pharmacotherapy)."

Point 2e.
As previously said, the therapeutic synergistic benefit provided by our drug association (gemcitabine + SOM230) will have to be validated on immune competent model(s) of PDAC, such as the genetically engineered KPC mouse model which presents intra-pancreatic mutations of Kras and p53. Mutated Kras only mice may not be useful for such experiments because spontaneous tumors appear irregularly and at late stages (Hingorani SR, Cancer Cell 2013).

Accordingly, the paragraph below has been included in the discussion:

"Three different xenografting procedures in immunodeficient mice, i.e. orthotopic (intrapancreatic)

or subcutaneous co-xenografting of human pancreatic cancer cells and human primocultures of CAFs, and subcutaneous xenografting of human pancreatic tumor (PDX, Patient-Derived tumor Xenografting) were used. These three strategies reproducibly demonstrate that combining the SOM230 treatment with the chemotherapeutic drug gemcitabine provides a therapeutic benefit over each single treatment. Through its indirect action on CAFs, SOM230 enables the re-sensitization of chemoresistant pancreatic cancer cells to gemcitabine's cytotoxicity. In these three models, our results demonstrate that the fibrotic stroma provided by the presence of CAFs and subsequent chemoprotection induced on pancreatic cancer cells mimics what occurs in human tumors, in contrast to xenografted models with pancreatic cancer cells alone (Perez-Mancera et al, 2012). Although the therapeutic synergistic benefit provided by our drug association will have to be validated on immune competent model(s) of PDAC, especially as CAFs also impact on immune cell function (Mace et al, 2013), our results introduce the novel proof of concept that protein synthesis is a promising therapeutic target in human CAFs."

Minor points:
a. "Cancerogenesis" has been replaced with "tumorigenesis". Grammar/synthax has been edited by a native English speaker.

b. 42 cases of human PDAC have now been used for sst1 / cytokeratin-19, and for sst1 / α-SMA (with quantifications), immunofluorescent co-stain experiments.

c. Methods for the conditional genetically engineered mouse model (GEM presenting an intra-pancreatic mutation of Kras) have been included.

3rd Editorial Decision 26 February 2015

Dear Dr. Bousquet,

We are pleased to inform you that your manuscript is accepted for publication and is now being sent to our publisher to be included in the next available issue of EMBO Molecular Medicine.
