# Supplementary figures and images for: Pharmacological targeting of the protein synthesis mTOR/4E-BP1 pathway in cancer-associated fibroblasts abrogates pancreatic tumour chemoresistance
Source: EMBO Mol Med. 2015 Apr 1;7(6):735–53. doi: 10.15252/emmm.201404346 (PMC4459815; doi:10.15252/emmm.201404346)

Duluc et al. Figure 1 (high resolution)

F

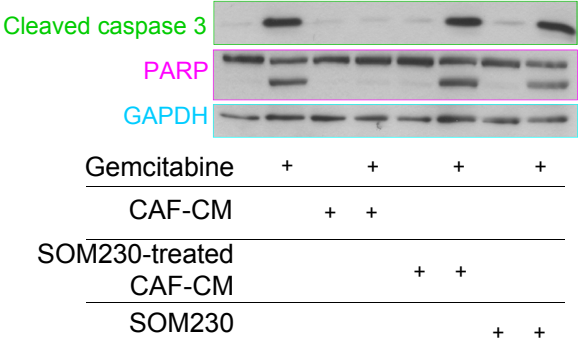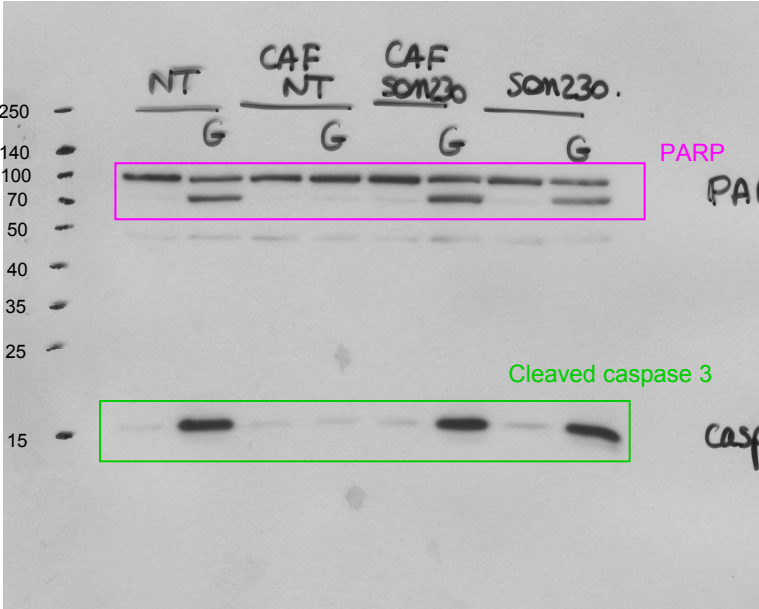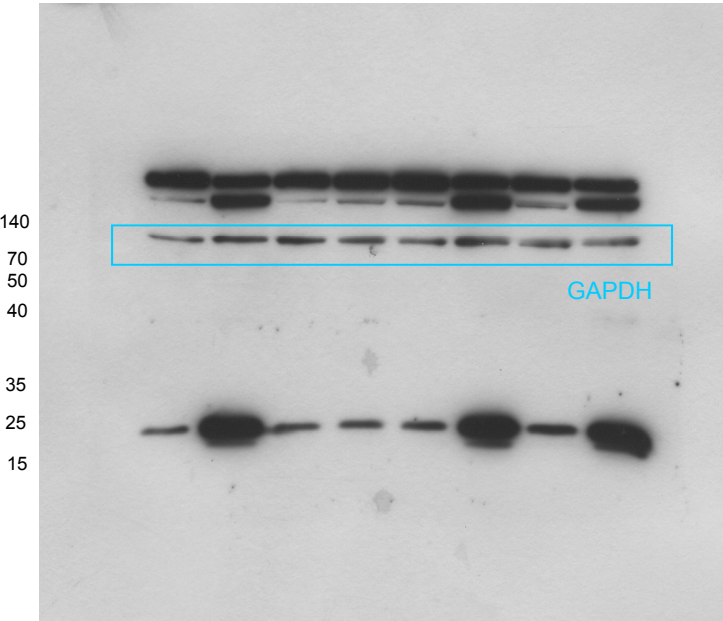

Supplement: Supplementary file 3 [file emmm0007-0735-sd3.pdf]

Duluc et al. Figure 2

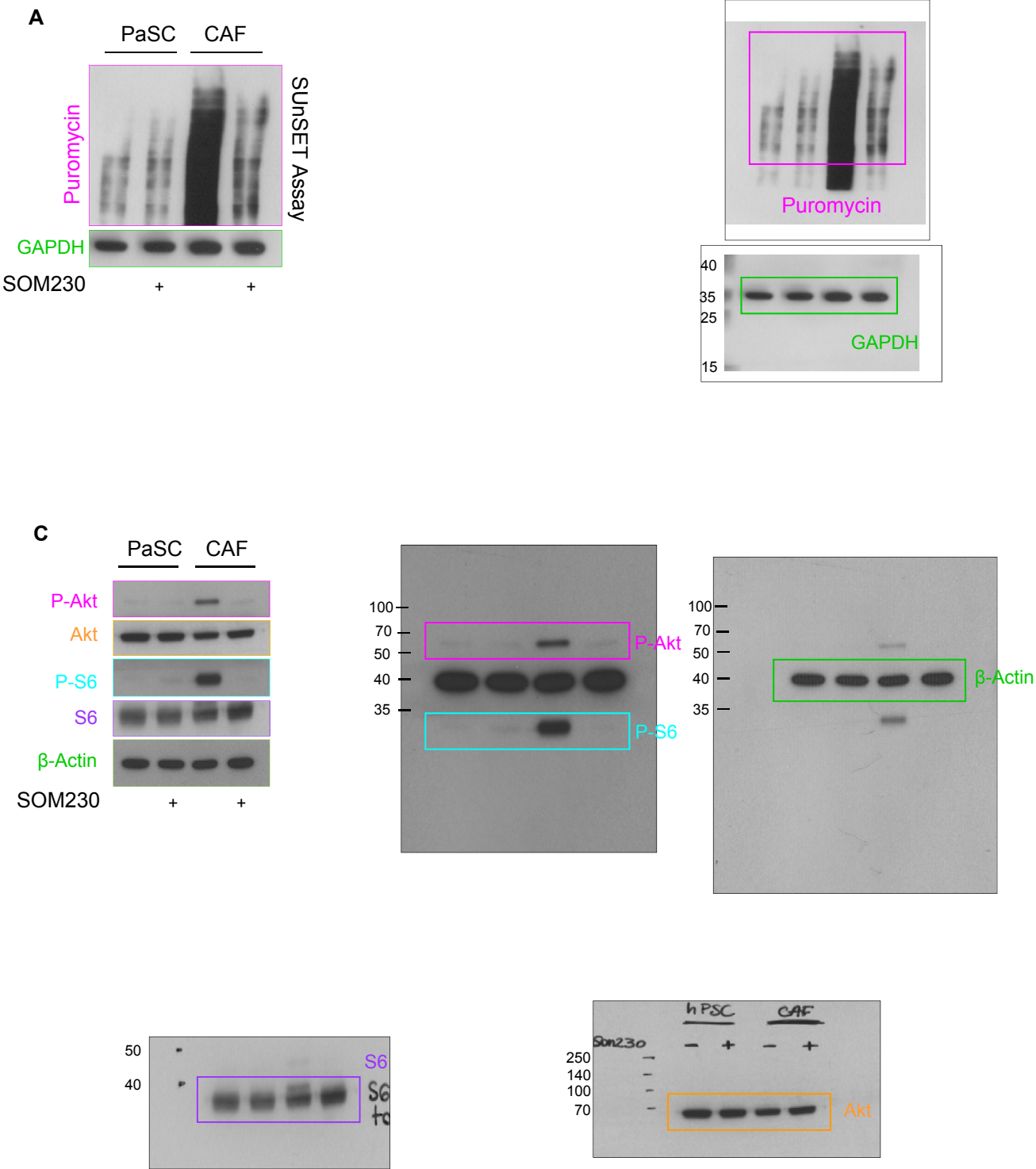

Duluc *et al.* Figure 2

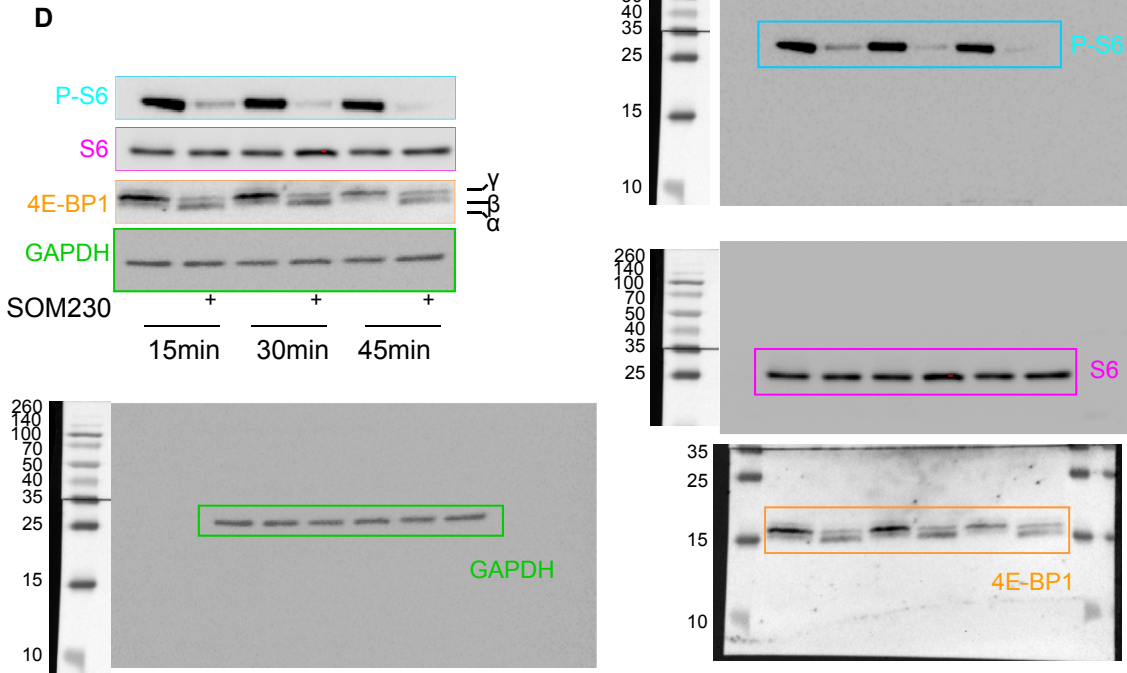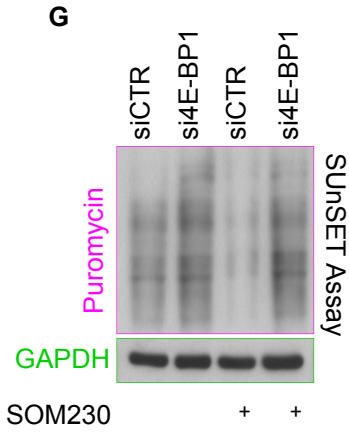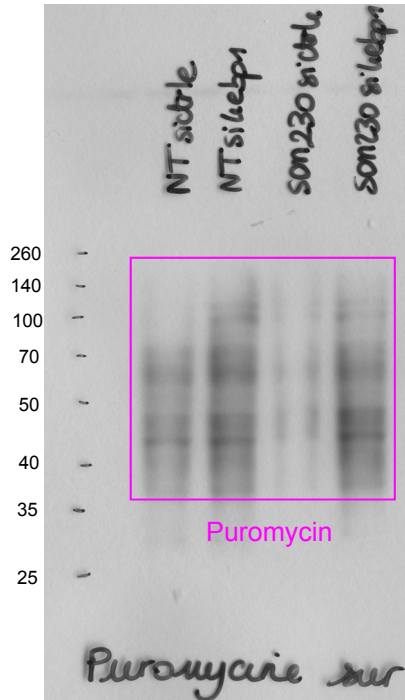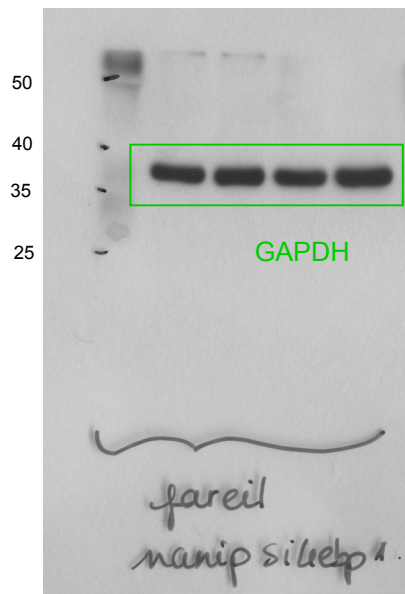

1

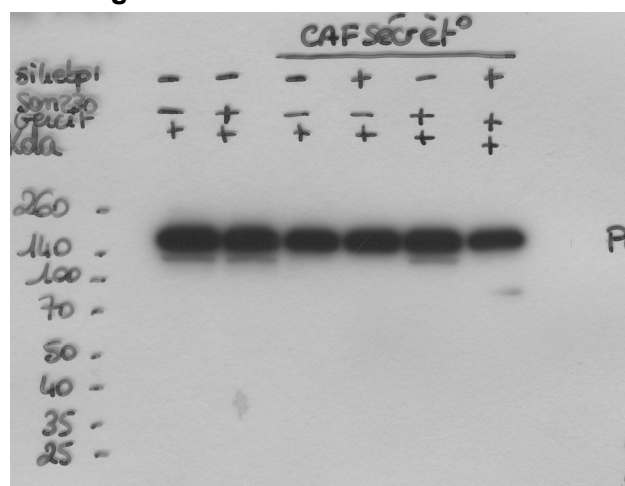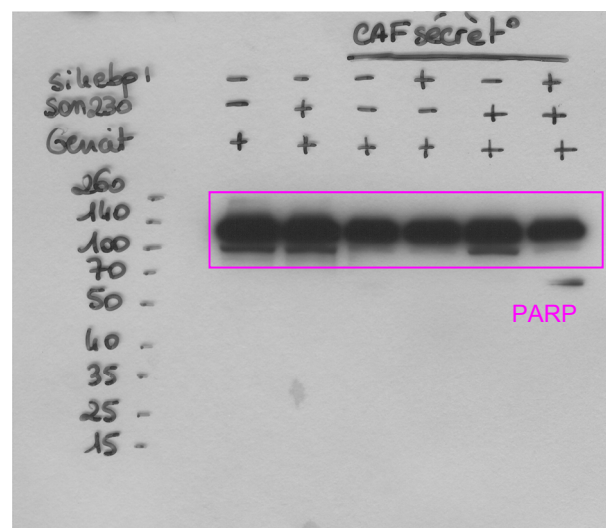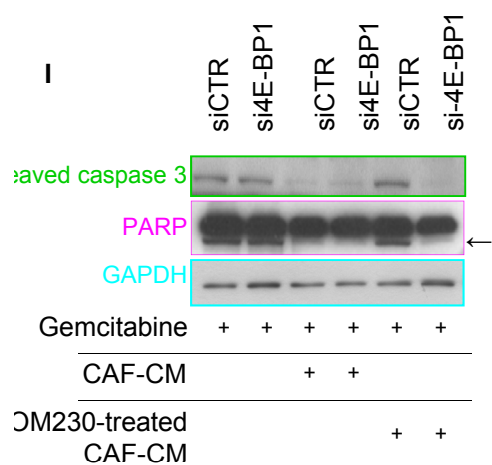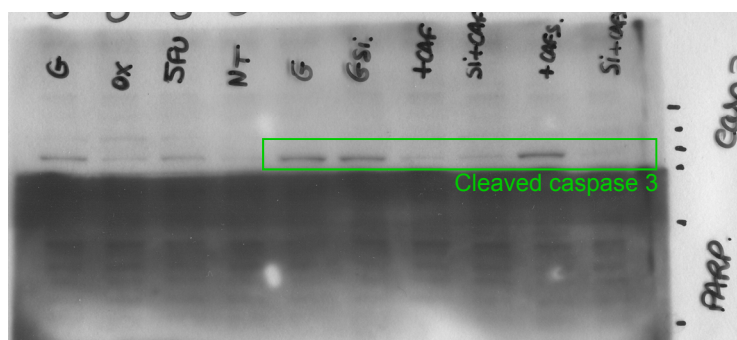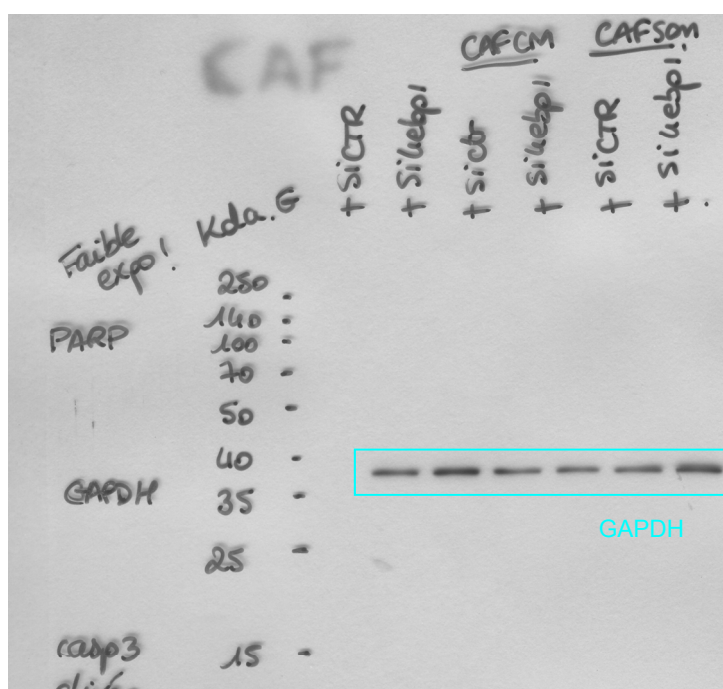

Supplement: Supplementary file 4 [file emmm0007-0735-sd4.pdf]

Duluc et al. Figure 3

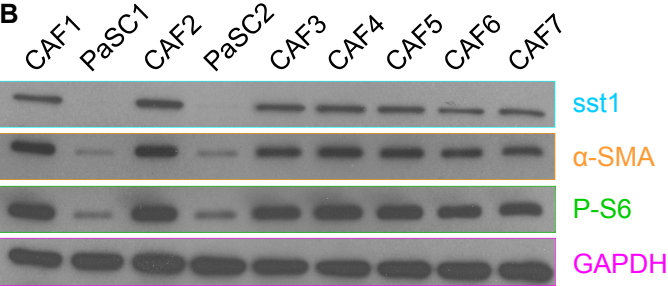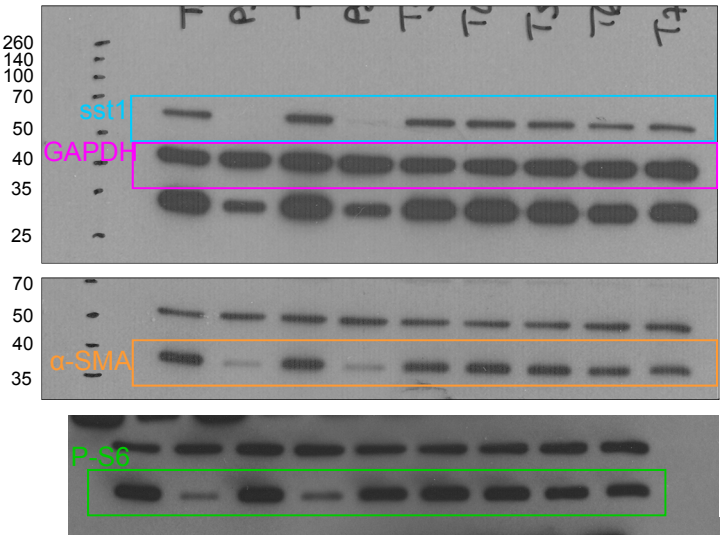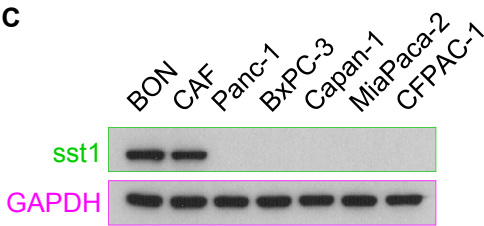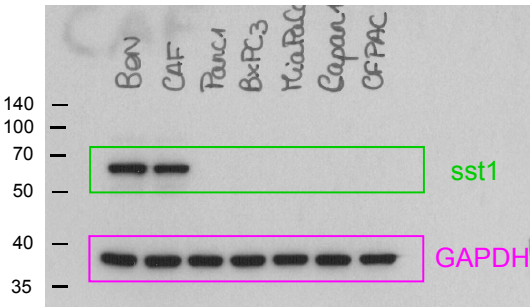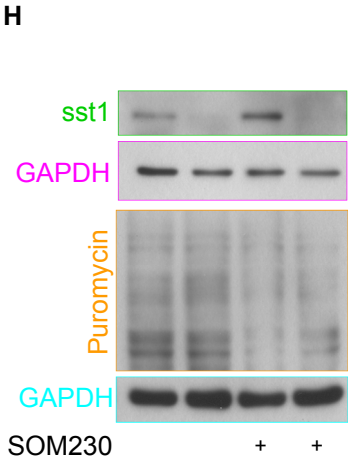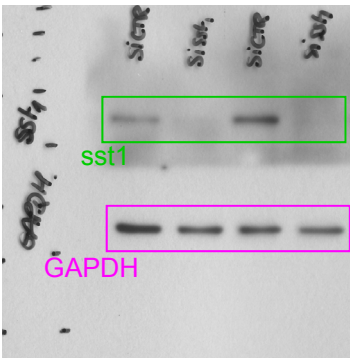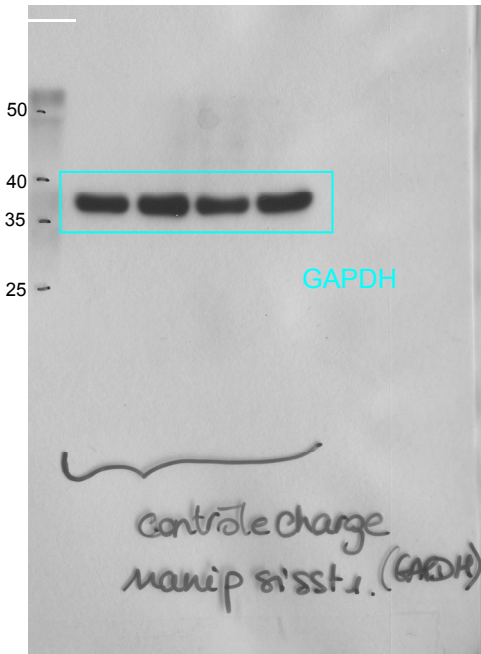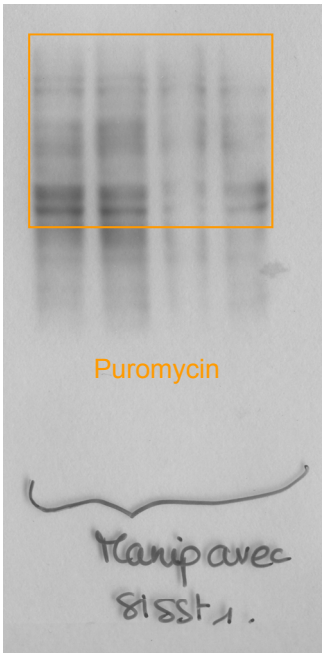

Duluc *et al.* Figure 3

I

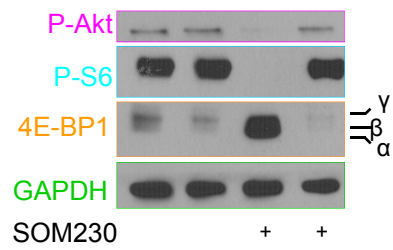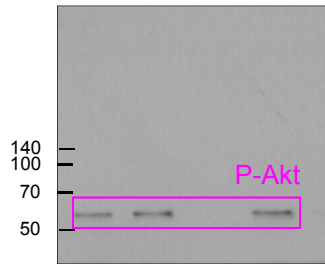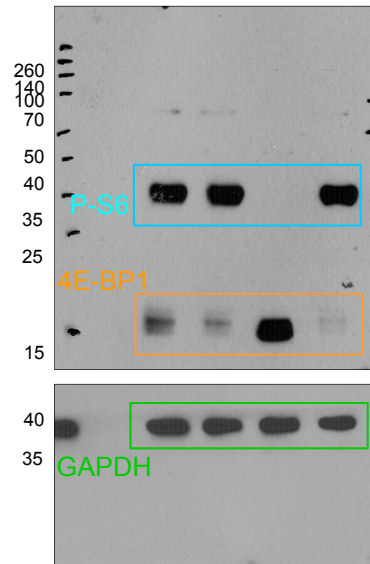

Supplement: Supplementary file 5 [file emmm0007-0735-sd5.pdf]

Duluc et al. Figure 4

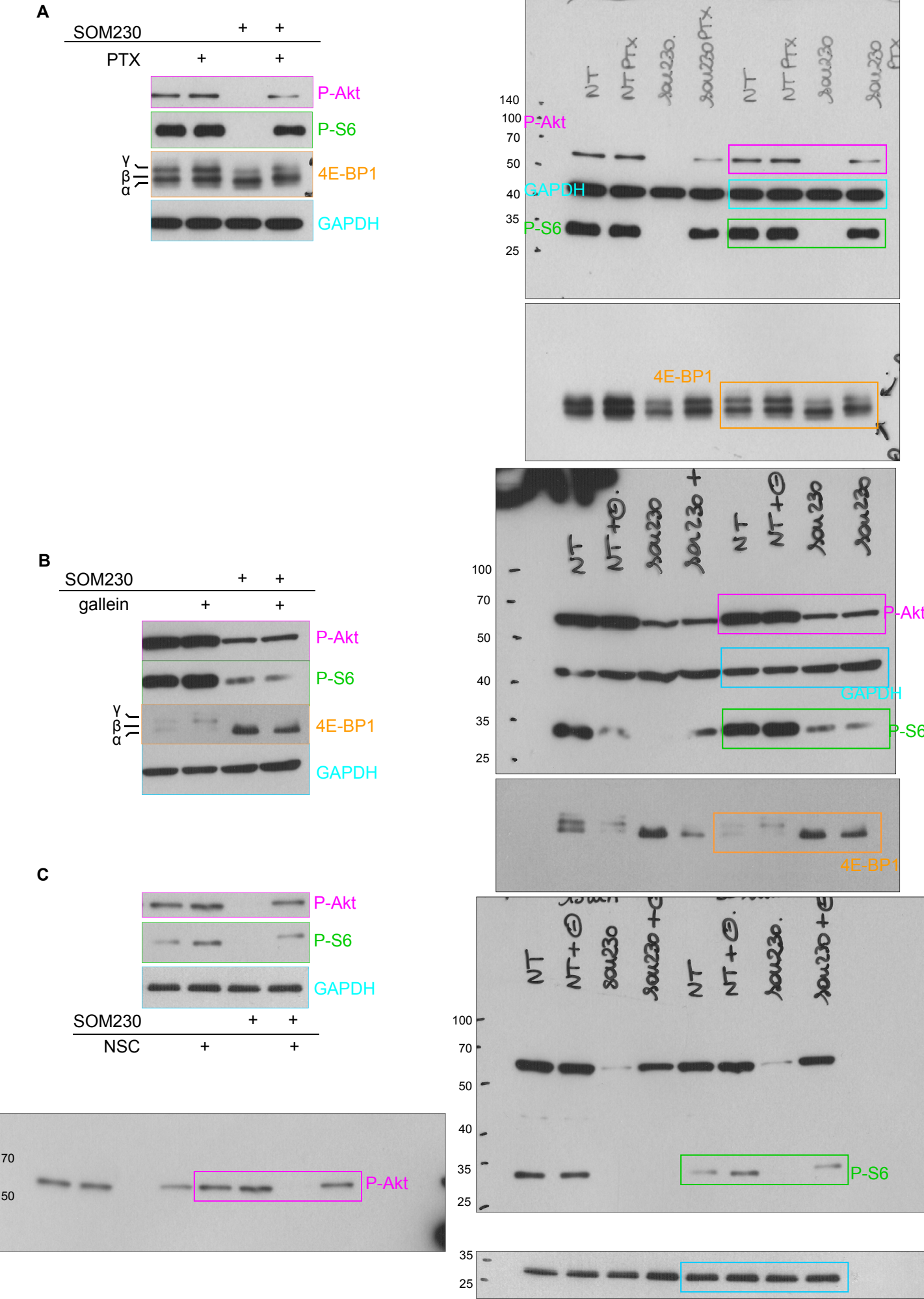

Duluc et al. Figure 4

D

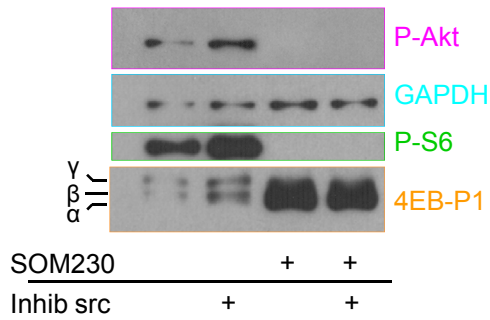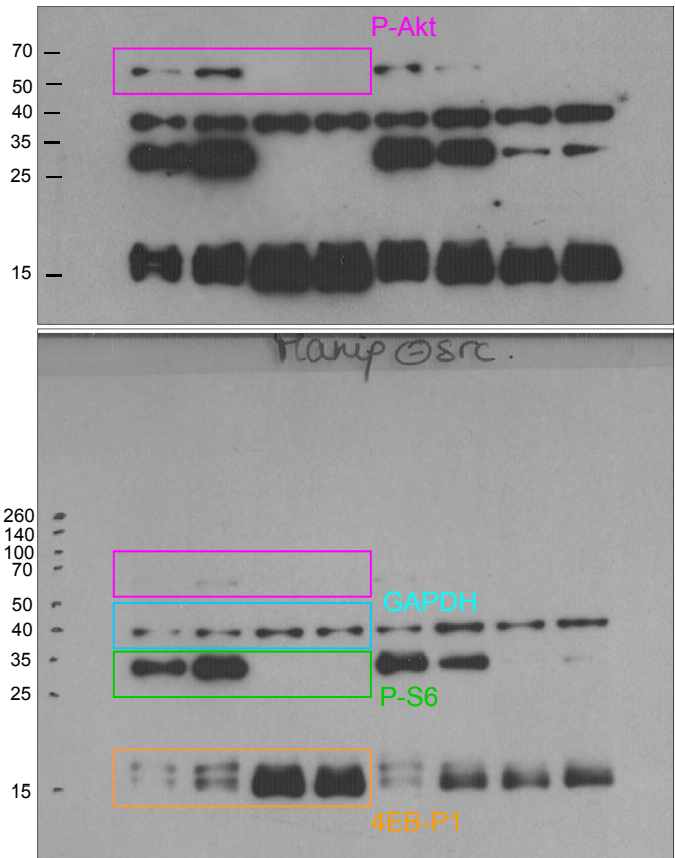

E

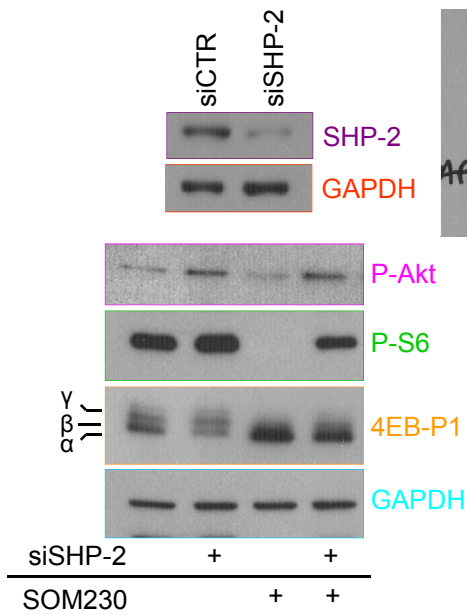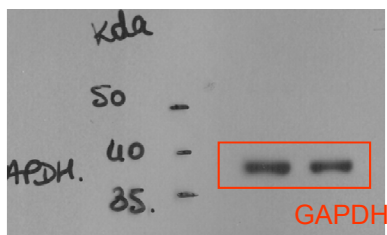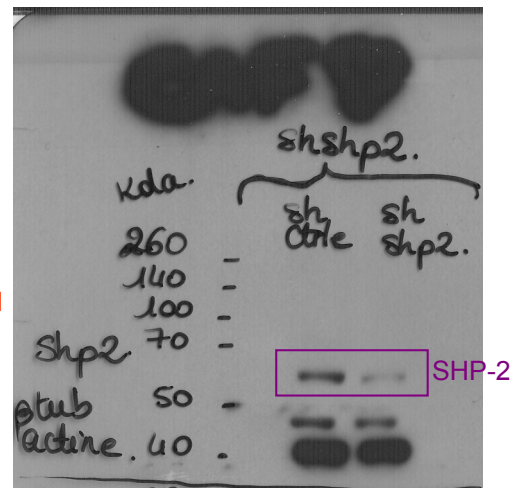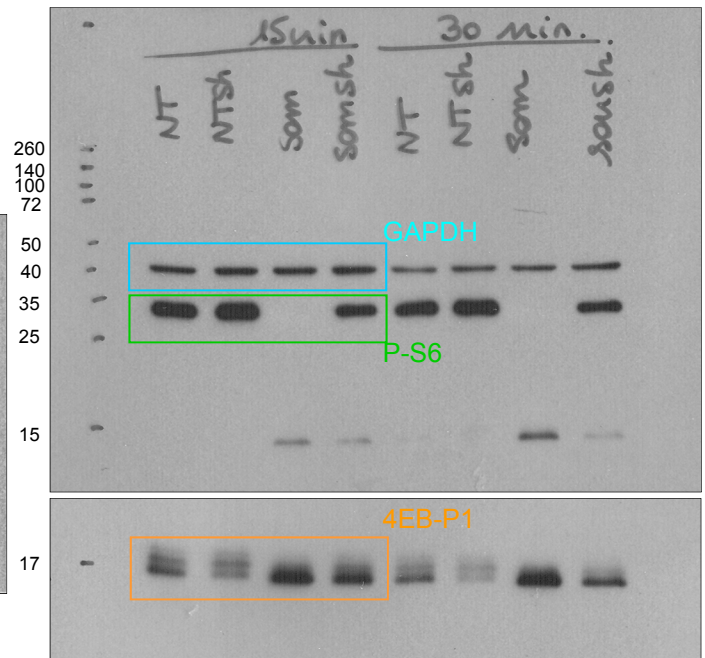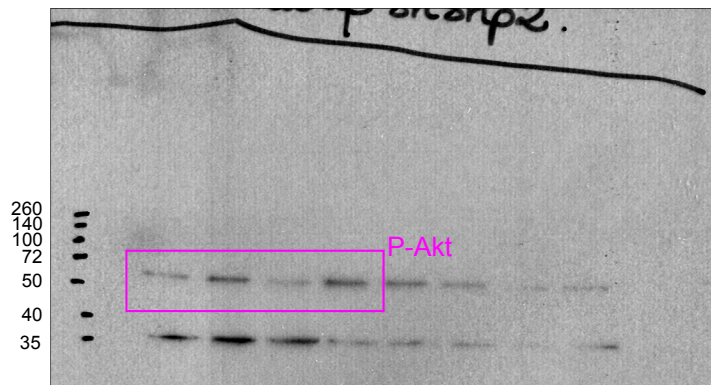

Duluc et al. Figure 4

F

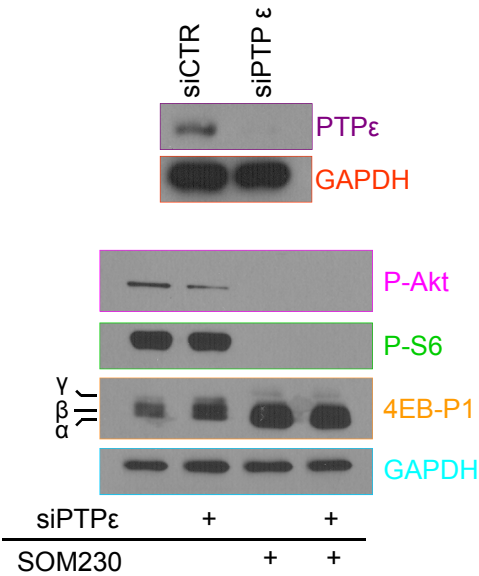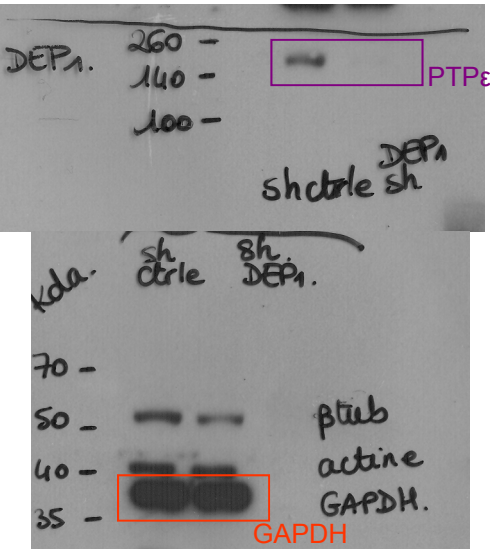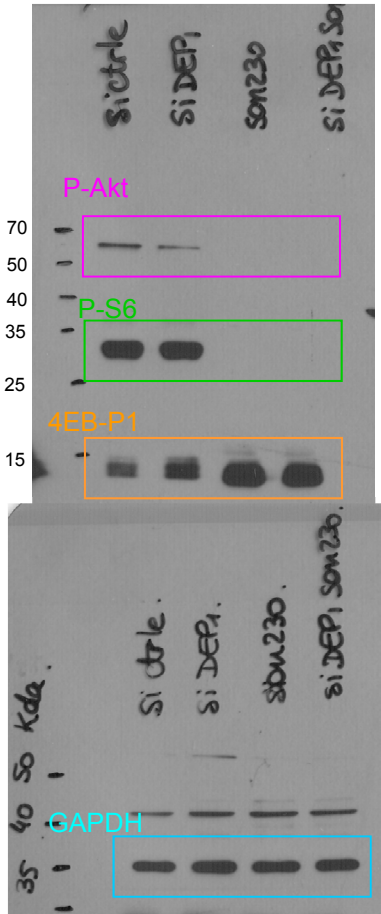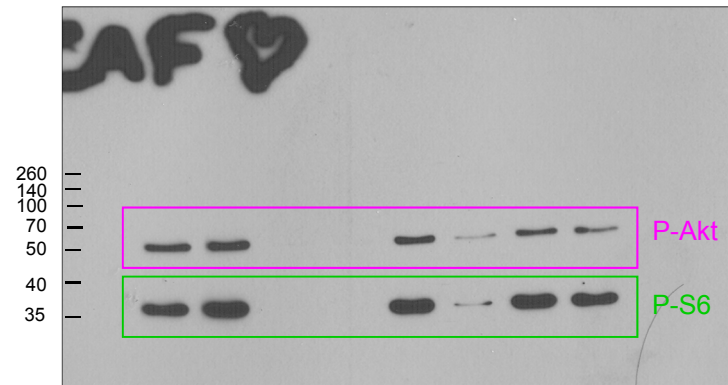

G

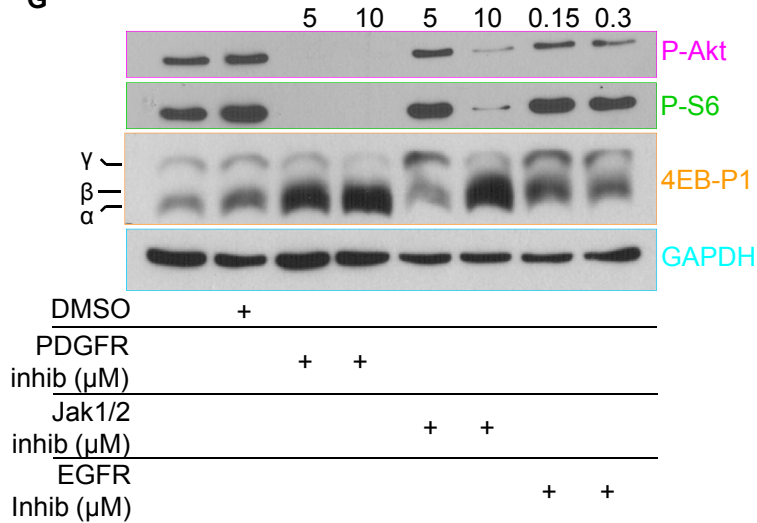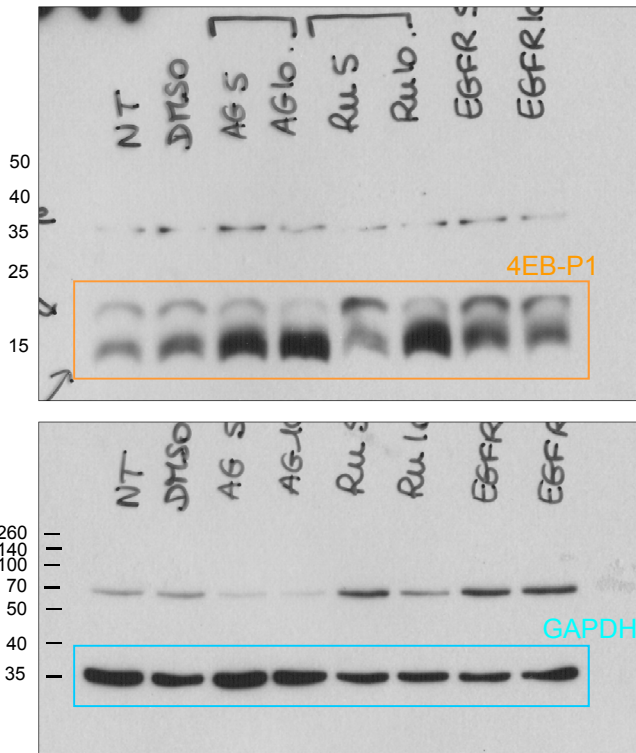

H

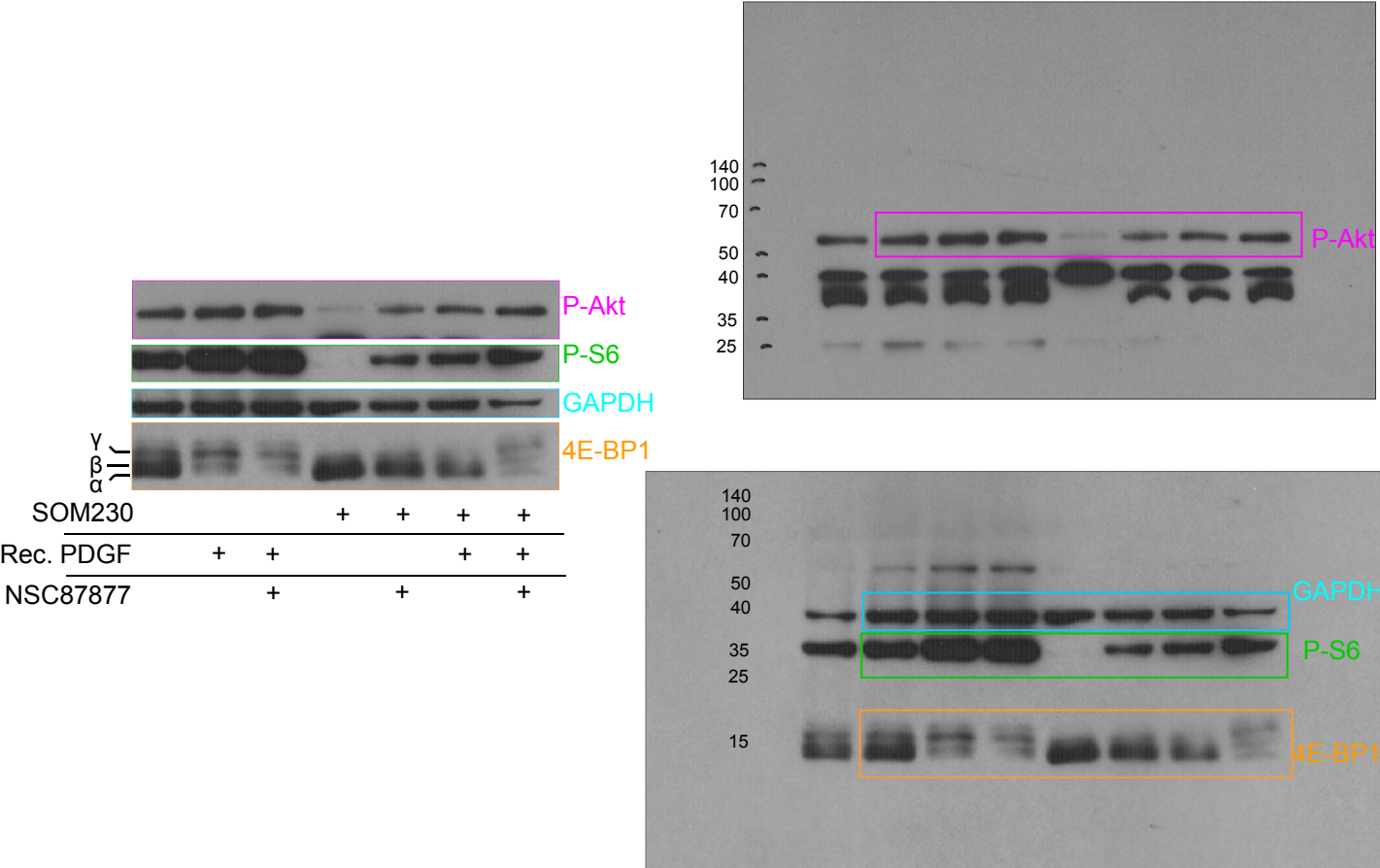

Supplement: Supplementary file 6 [file emmm0007-0735-sd6.pdf]

Duluc et al. Figure 6

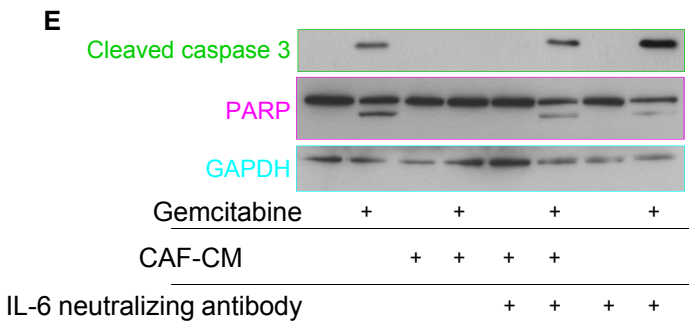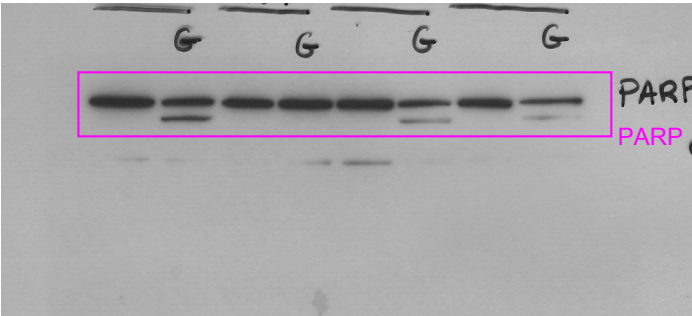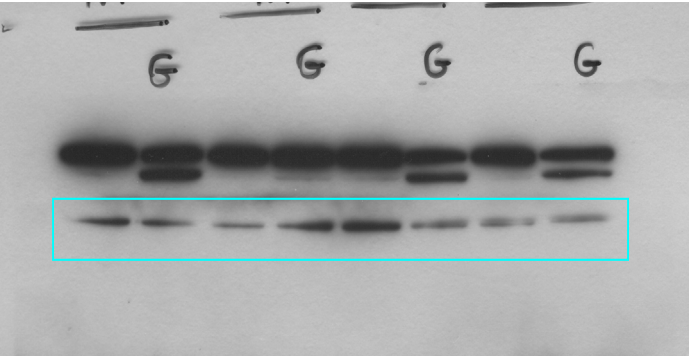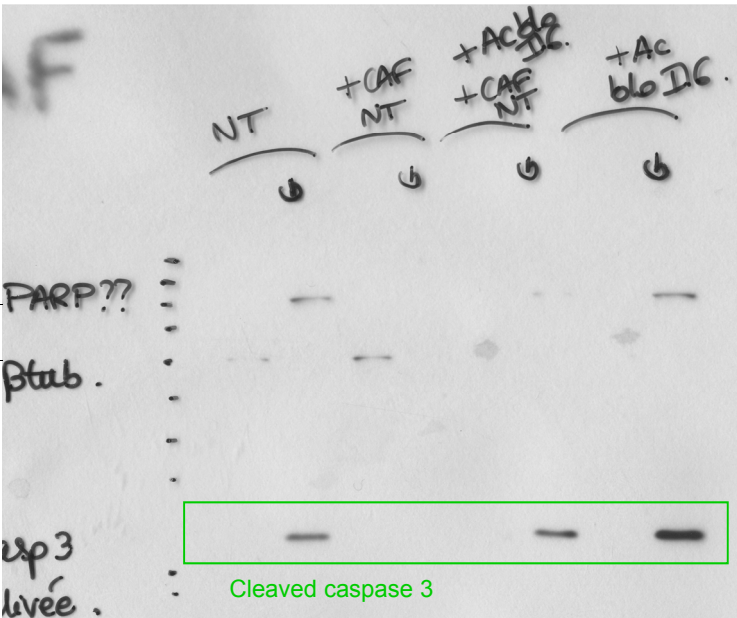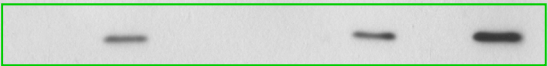

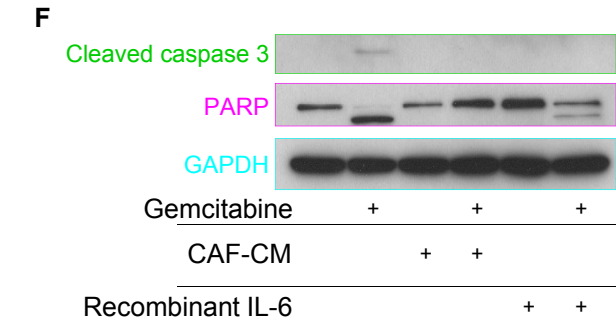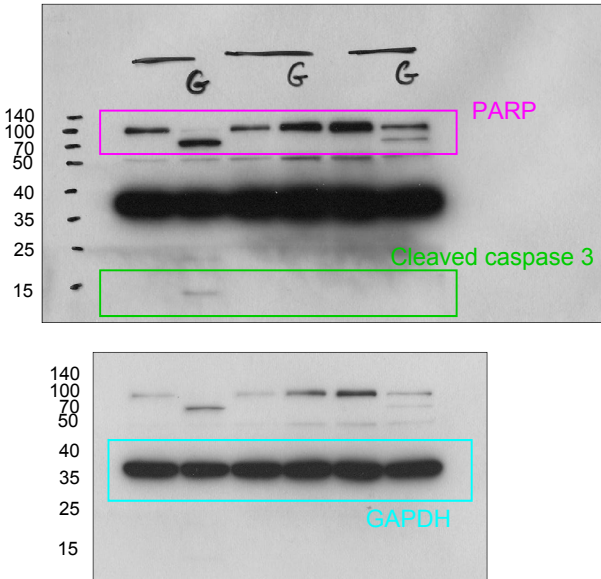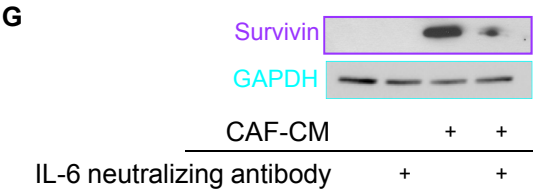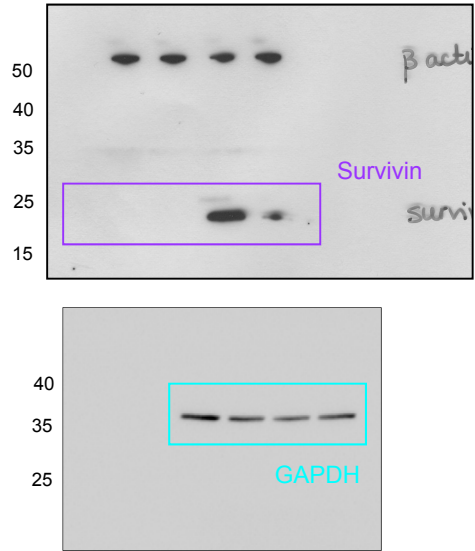

H

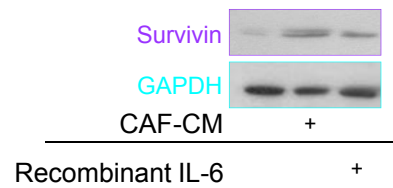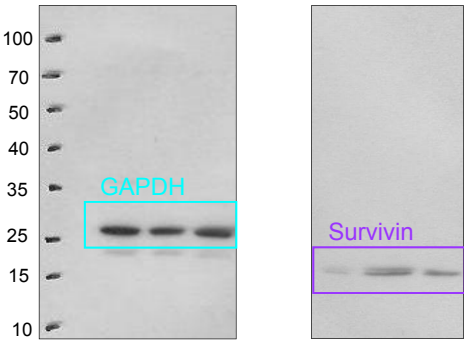

I

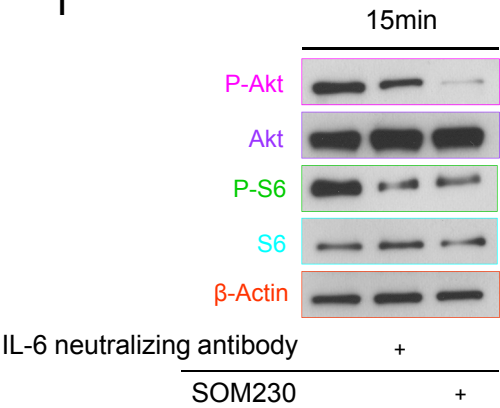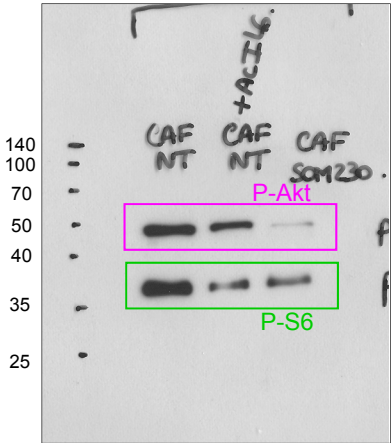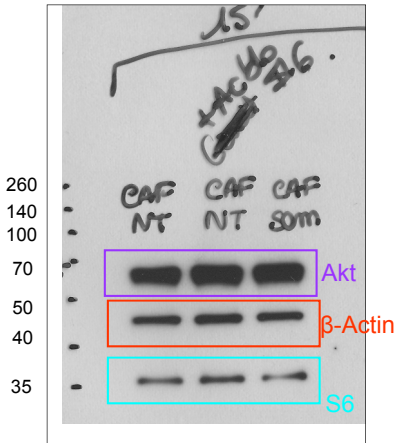

Supplement: Supplementary file 7 [file emmm0007-0735-sd7.pdf]
